# Enhanced Stibine Oxide Lewis Basicity Overcomes Steric Frustration

**DOI:** 10.1021/acs.organomet.5c00212

**Published:** 2025-09-09

**Authors:** Addis Getahun, John S. Wenger, Timothy C. Johnstone

**Affiliations:** Department of Chemistry and Biochemistry, 8787 University of California Santa Cruz, Santa Cruz, California 95064, United States

## Abstract

The characteristic electronic structure of the phosphoryl group in phosphine oxides confers great stability on the P^+^–O^–^ bond, in part because of back-bonding from O-based lone pairs into the P–C antibonding orbitals. The partial nature of this donation allows the O atom in the phosphoryl unit to exhibit Lewis basicity. This backbonding weakens as the atomic number of the pnictogen increases, which results in a significant enhancement in basicity for the heavier stiboryl congener. Here, we compare the ability of R_3_PnO (Pn = P, As, Sb) species to bind to main-group Lewis acids. As the steric bulk of the R group increases, R_3_PO and R_3_AsO lose this capacity; Dipp_3_PO and Dipp_3_AsO (where Dipp = 2,6-diisopropylphenyl) are unable to bind even the very strong Lewis acid B­(C_6_F_5_)_3_. In contrast, the enhanced basicity of the stibine oxides allows them to overcome this steric hindrance and form adducts, even in the case of the very hindered Dipp_3_SbO·B­(C_6_F_5_)_3_.

## Graphical abstract

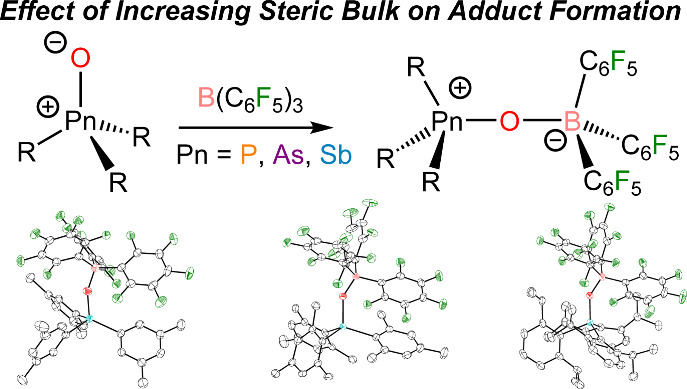

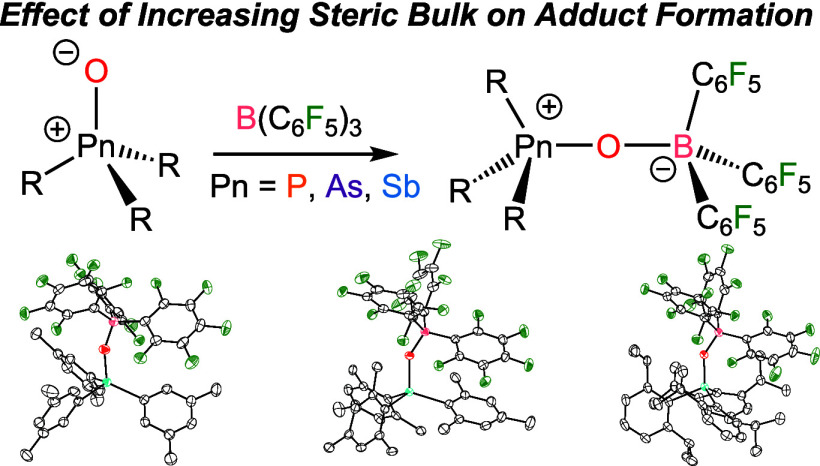

## Introduction

The electronic structure of the phosphoryl bond confers exceptional stability on phosphine oxides, allowing their formation to serve as the thermodynamic driving force in transformations such as the Wittig reaction and Staudinger reduction. The P–O bonding interaction in R_3_PO molecules has been debated, but a currently well-accepted model invokes a σ bond formed with the p_*z*_ orbital of the O atom and backbonding from the p_*x*_ and p_*y*_ orbitals of the O atom into P–C σ* orbitals.^1^ This backbonding strengthens the P–O bond but a significant amount of charge remains concentrated on the O atom, allowing phosphine oxides to serve as Lewis bases.^2^ This Lewis basicity has been exploited to form metal–ligand complexes,^3^ particularly with hard metal ions such as those of the Ln­(III) series.^4^ Phosphine oxides also readily interact with main-group Lewis acids and spectroscopic studies confirm that a variety of phosphine oxides form stable adducts with the strong Lewis acid B­(C_6_F_5_)_3_.^5,6^ Indeed, Et_3_PO serves as the most common reporter base in the widely used Guttman–Beckett assay that is used to measure the strengths of Lewis acids, including B­(C_6_F_5_)_3_.^5–8^ Newer methods continue to be developed, with fluorescent phosphine oxides being used to optically measure Lewis acid strength.^9^ It is noteworthy that some phosphines, such as ^t^Bu_3_P, that are too sterically encumbered to coordinate to B­(C_6_F_5_)_3_,^10^ are able to form stable adducts with this acid once oxidized to the phosphine oxide.^11^

Arsine oxides exhibit greater Lewis basicity than their phosphine oxide congeners, which has been ascribed to the lesser extent of donation from the O-based lone pairs into the As–C σ* orbitals.^12^ Although the energies of the O-based lone pairs and As–C σ* orbitals approach each other more closely and the σ*-based orbitals gain a more pnictogen character, the lengthening of the Pn–O bond and the greater diffuseness of the Pn-based orbitals ultimately result in less favorable back-bonding. It was recognized early in the systematic investigation of the chemistry of arsine oxides that this enhanced Lewis basicity could be exploited to form stable complexes with transition metals.^13^ The increase in donicity can productively modulate the properties of the resulting species, such as the luminescence of lanthanide complexes.^14–16^ Similar to Ph_3_PO,^6^ Ph_3_AsO forms a stable adduct with strong main-group Lewis acids like B­(C_6_F_5_)_3_.^17^

We recently reported the synthesis and isolation of the first monomeric stibine oxides, Dipp_3_SbO and Mes_3_SbO.^12,18–20^ Our computational data suggested that stibine oxides would exhibit an even lesser degree of back-bonding from the O-based lone pairs into the Pn–C antibonding orbitals. We observed that this variation was reflected in experimentally determined Bro̷nsted basicities, which vary by well over one million-fold across the series Dipp_3_PnO (Pn = P, As, Sb).^21^ An exploratory reaction between Dipp_3_SbO and BF_3_·OEt_2_ resulted not in simple acid–base adduct formation but rather in an unexpected O^2–^/2F^–^ exchange and clean formation of Dipp_3_SbF_2_.^12^ It has been previously reported that reaction of B­(C_6_F_5_)_3_ with polymeric (Ph_3_SbO)_*n*_ results in disassociation of the stibine oxide units and affords the monomeric adduct Ph_3_SbO·B­(C_6_F_5_)_3_ (Scheme 1).^17^ We have previously observed that Dipp_3_SbO was too sterically encumbered to react with a series of unencumbered group 14 Lewis acids but that reactions proceeded readily with Mes_3_SbO.^18^ Here, we demonstrate that the variation in the electronic structure of R_3_PnO species as the pnictogen increases in atomic number confers an exceptional enhancement in Lewis basicity on the stibine oxides. Although Ph_3_PnO species form Ph_3_PnO·B­(C_6_F_5_)_3_ adducts for Pn = P, As, and Sb,^6,17^ we demonstrate here that isolable Mes_3_PnO·B­(C_6_F_5_)_3_ adducts only form for Pn = As and Sb; the steric bulk of the mesityl groups results in an equilibrium mixture of Mes_3_PO/B­(C_6_F_5_)_3_ and Mes_3_PO·B­(C_6_F_5_)_3_. The more hindered Dipp_3_PO does not show any evidence of interaction with B­(C_6_F_5_)_3_, nor does Dipp_3_AsO. In contrast, Dipp_3_SbO forms a stable Dipp_3_SbO·B­(C_6_F_5_)_3_ adduct with no spectroscopic evidence of dissociation or exchange in solution (Scheme 1). We study the impact of steric hindrance on R_3_SbO·B­(C_6_F_5_)_3_ adduct formation by comparing R = Dipp, Mes, and 3,5-Me_2_Ph and additionally computationally investigate the thermodynamics of these reactions and probe the variation in the nature of the B–O bonds across the different adducts.

**Scheme 1:**
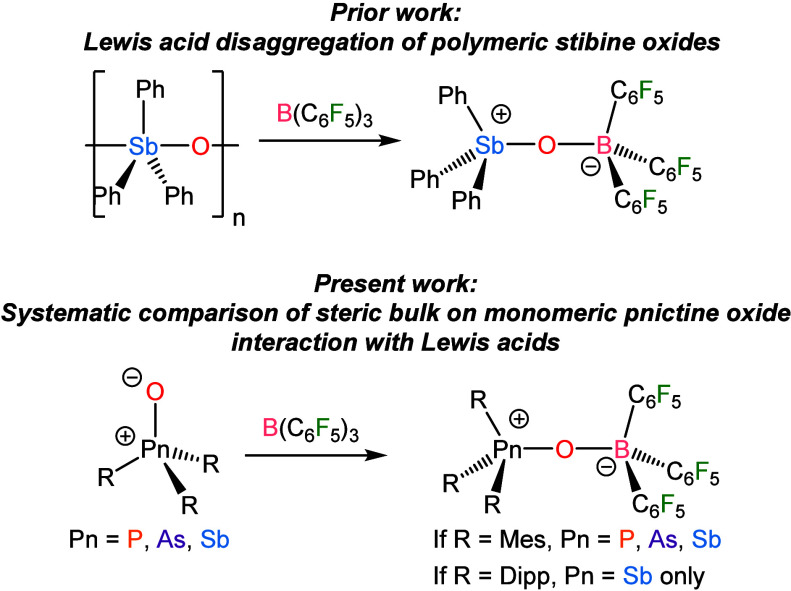
Prior Work Demonstrating That Polymeric Stibine Oxides Can Be Disaggregated with Lewis Acids and the Present Work on the Influence of Steric Bulk on the Reaction of Lewis Acids with Monomeric Pnictine Oxides

## Results and Discussion

### Reaction of Mes_3_SbO with Al­(OEt)_3_ and B­(OEt)_3_

On the basis of our earlier results highlighting the Bro̷nsted basicity of monomeric stibine oxides,^21^ we anticipated that they would similarly exhibit an enhanced Lewis basicity, as compared to the lighter congeners. We first explored the reactivity of Mes_3_SbO with Al­(OEt)_3_, given the established reactivity of phosphine oxides with Al­(OR)_3_ compounds, where R is an alkyl or silyl group.^22–24^ As expected, combination of these species readily afforded the adduct Mes_3_SbO·Al­(OEt)_3_ as an analytically pure solid (Scheme 2). Subtle changes occur in the ^1^H and ^13^C NMR chemical shifts of resonances from the mesityl rings, as compared to free Mes_3_SbO, and the integrations of these signals as compared to the O*Et* resonances confirm the formulation suggested by the microanalytical data. Single-crystal X-ray diffraction data further confirmed the identity of the product (Figure S48). The molecule crystallized in space group *P*3̅*c*1 and resides on a crystallographic 3-fold rotation axis. The O–Al bond length is 1.719(10) Å, which is shorter than the sum of the single-bond covalent radii for these elements (1.89 Å), highlighting the strength of the Lewis adduct. The Sb–O bond length of 1.904(9) Å is significantly lengthened from the value of 1.8481(16) Å of free Mes_3_SbO.^18^

**Scheme 2:**
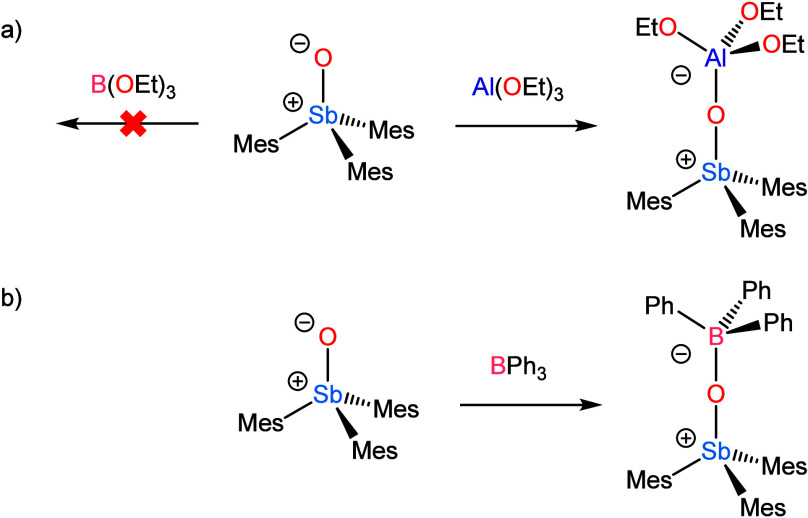
Reactivity of Mes_3_SbO with (a) M­(OEt)_3_ where M = B or Al, and (b) BPh_3_

Encouraged by the strength of the interaction with Al­(OEt)_3_, we tested the ability of Mes_3_SbO to form an adduct with B­(OEt)_3_, but only unreacted Mes_3_SbO was obtained following a workup analogous to that described above for Mes_3_SbO·Al­(OEt)_3_. NMR spectra of Mes_3_SbO with 1–10 equiv of B­(OEt)_3_ show no evidence of interaction between the two molecules (Figures S3 and S4). This result is unsurprising because trialkoxyboranes are among the weakest neutral trisubstituted B-based Lewis acids.^25,26^ Donation from the O-based lone pairs into the empty B-based orbital is typically invoked to explain this attenuated acidity, and no isolable intermolecular Lewis adducts of phosphine oxides or arsine oxides with B­(OR)_3_ are known when R is a simple alkyl or aryl group. It is noteworthy that a sufficiently large inductive effect can apparently override this lone-pair delocalization: very recently, the adduct Ph_3_PO·B­(OTeF_5_)_3_ was isolated and characterized.^27^

### Reaction of Mes_3_SbO with BPh_3_

Given the lack of interaction between Mes_3_SbO and B­(OEt)_3_, we explored the interaction of this stibine oxide with the stronger, albeit still relatively weak, Lewis acid BPh_3_.^26^ Combination of toluene solutions of the two species resulted in the precipitation of a colorless solid, whose limited solubility in a variety of organic solvents precluded NMR spectroscopic characterization. If the toluene solutions of the two reactants were layered and allowed to mix slowly by diffusion, then diffraction-quality crystals could be obtained (Figure 1). Single-crystal X-ray diffraction revealed the product to be Mes_3_SbO·BPh_3_ and powder diffraction confirmed that the bulk sample comprised the same substance. The molecule crystallized in space group *P*3̅ and sits on a 3-fold rotation axis. The B–O bond length is 1.546(5) Å, which is significantly longer than the sum of the single-bond covalent radii of these elements (1.48 Å), and the Sb–O distance is 1.829(3) Å. This latter value is nearly unchanged from the Sb–O bond length of the free stibine oxide.^18^ Although the B center is unquestionably pyramidalized with a nearly ideal tetrahedral C–B–C angle of 109.99(15)°, these bond metrics collectively indicate that the interaction of Mes_3_SbO with BPh_3_ is weak.

**Figure 1:**
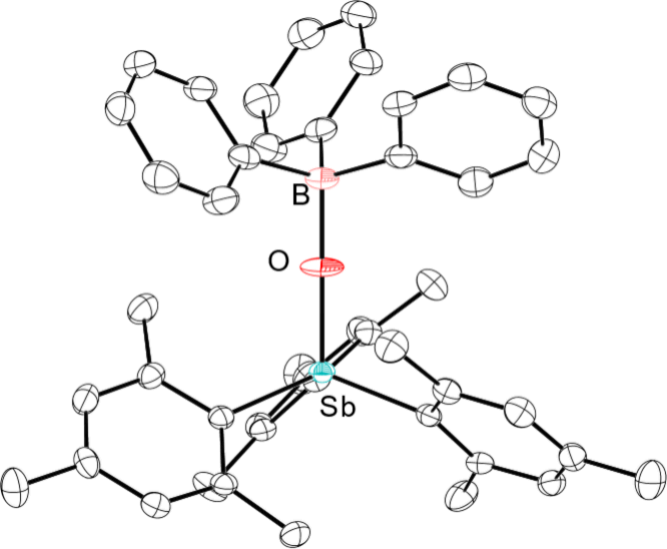
Thermal ellipsoid plot of Mes_3_SbO·BPh_3_. Ellipsoids are shown at the 50% probability level. H atoms have been omitted for the sake of clarity. Color code: Sb light blue, B pink, O red, C black.

### Reaction of Mes_3_PnO with B­(C_6_F_5_)_3_

In contrast, the combination of Mes_3_SbO and the stronger Lewis acid B­(C_6_F_5_)_3_ results in the formation of a strong adduct (Scheme 3). In CDCl_3_, the product of the reaction displays a single ^11^B NMR signal at −0.75 ppm, and three sharp ^19^F NMR signals with Δδ_m,p_ = 5.1 ppm, both of which are consistent with a 4-coordinate B center. The single sets of sharp ^19^F and ^13^C signals stand in contrast to the ^1^H NMR spectrum, which features a mix of sharp and broad signals that decoalesce upon cooling to −20 °C (Figures S6 and S7), consistent with a hindered internal rotation that occurs on the NMR time scale. Single-crystal X-ray diffraction identified the product as Mes_3_SbO·B­(C_6_F_5_)_3_ and confirmed the steric congestion (Figure 2). The greater degree of congestion, as compared to Mes_3_SbO·BPh_3_, stems not only from the substitution around the periphery of the B-bound aryl ring but also from the decreased B–O distance of 1.488(5) Å. The greater Lewis acidity of B­(C_6_F_5_)_3_ results in the formation of a stronger B–O bond. There is also a concomitant lengthening of the Sb–O distance to 1.884(3) Å from the distance of 1.848(2) Å in the free monomeric Mes_3_SbO.^18^

**Scheme 3:**
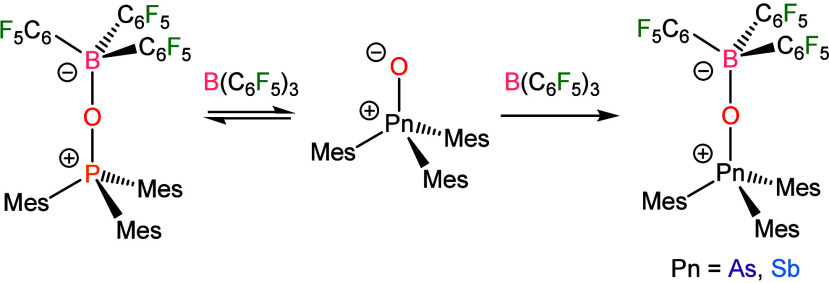
Reactions of Mes_3_PnO (Pn = P, As, Sb) with B­(C_6_F_5_)_3_

**Figure 2:**
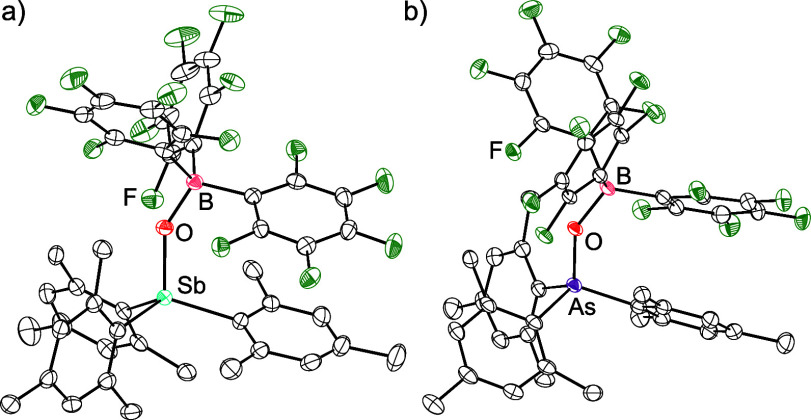
Thermal ellipsoid plots of (a) Mes_3_SbO·B­(C_6_F_5_)_3_ and (b) Mes_3_AsO·B­(C_6_F_5_)_3_. Ellipsoids are shown at the 50% probability level. H atoms and solvent molecules have been omitted for the sake of clarity. For (b), only one of the two crystallographically independent molecules in the asymmetric unit is shown. Color code: Sb light blue, As purple, B pink, O red, C black, F green.

As with Mes_3_SbO, Mes_3_AsO was able to readily bind B­(C_6_F_5_)_3_ and formed the adduct Mes_3_AsO·B­(C_6_F_5_)_3_ (Figure 2). Although isolable, solutions of the product would gradually decompose over time with signals from hydrolysis products such as [Mes_3_As­(OH)]­[B­(C_6_F_5_)_3_(OH)] developing. The NMR signals of Mes_3_AsO·B­(C_6_F_5_)_3_ presented similarly to those of Mes_3_SbO·B­(C_6_F_5_)_3_, but with full decoalescence of the signals at room temperature. The greater degree of steric congestion expected as a result of a shorter Pn–O bond would be consistent with this result. Indeed, the As–O distance of 1.7094(15) Å is less than the Sb–O distance above but close to the As–O distance of 1.698(2) Å in Ph_3_AsO·B­(C_6_F_5_)_3_. The B–O distance of 1.533(3) Å in Mes_3_AsO·B­(C_6_F_5_)_3_ is longer than that of 1.521(3) Å in the Ph_3_AsO adduct. This result is consistent with the influence of steric congestion described above.

In contrast, Mes_3_PO and B­(C_6_F_5_)_3_ form an equilibrium mixture of the starting materials and Mes_3_PO·B­(C_6_F_5_)_3_. Intermediate exchange between the free and adducted species results in broad signals in the ^1^H and ^31^P NMR spectra at room temperature. Cooling to −20 °C shifts the equilibrium and slows the rate of exchange sufficiently that signals, although still broad, can be observed (Figures S25–S28). The breadth of the signals, even at low temperature, suggests a highly dynamic equilibrium, and the adduct could not be isolated. Because Ph_3_PO, Ph_3_AsO, and Ph_3_SbO all form stable adducts with B­(C_6_F_5_)_3_,^6,17^ we ascribe the weakened interaction between Mes_3_PO and B­(C_6_F_5_)_3_ to a combination of reduced basicity (as compared to the heavier congeners) and increased steric bulk (as compared to Ph_3_PO).

### Reaction of Dipp_3_PnO with B­(C_6_F_5_)_3_

The data above demonstrate that the steric pressure introduced by mesityl substituents is sufficient to disrupt the formation of a strong Mes_3_PO·B­(C_6_F_5_)_3_ adduct, but not Mes_3_AsO·B­(C_6_F_5_)_3_ or Mes_3_SbO·B­(C_6_F_5_)_3_. We therefore investigated whether adduct formation would persist in the face of a further increased steric encumbrance. Combination of B­(C_6_F_5_)_3_ and either Dipp_3_PO or Dipp_3_AsO resulted in no Dipp_3_PnO·B­(C_6_F_5_)_3_ adduct formation, as assessed by NMR spectroscopic measurements on the reaction mixtures (Scheme 4, Figures S34–S40). In the absence of pnictine oxide binding to the borane, residual ether could be observed coordinating. In the case of the mixture of Dipp_3_PO and B­(C_6_F_5_)_3_, additional weak signals (e.g., [B­(C_6_F_5_)_3_(OH)]^−^) would develop in the NMR spectra over time, which we attribute to the unquenched reactivity of acid and base acting upon trace moisture.

**Scheme 4:**
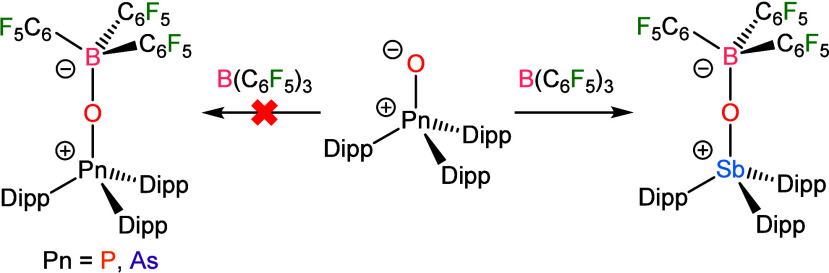
Reactions of Dipp_3_PnO (Pn = P, As, Sb) with B­(C_6_F_5_)_3_

In contrast, Dipp_3_SbO and B­(C_6_F_5_)_3_ react to form a single new species. In mixtures of Dipp_3_SbO and B­(C_6_F_5_)_3_ in CDCl_3_, a ^11^B NMR signal appears at −0.37 ppm and Δδ_m,p_(^19^F) = 5.3 ppm, strongly suggesting that a stable adduct persists in solution. For this Dipp_3_SbO·B­(C_6_F_5_)_3_ adduct, even at room temperature, the ^1^H NMR signals are sharp and fully desymmetrized (Figure S29), suggesting that rotation about the Sb–C bond has been significantly slowed as compared to the less hindered stibine oxides. In the ^19^F NMR spectrum, the ortho signals are decoalesced and broad (Figure S31). These results point to the much greater degree of steric congestion present in Dipp_3_SbO·B­(C_6_F_5_)_3_ but also to the persistence of the B–O interaction. An EXSY experiment performed on a mixture of B­(C_6_F_5_)_3_ and 2 equiv Dipp_3_SbO, which forms one equivalent of the Dipp_3_SbO·B­(C_6_F_5_)_3_ adduct and leaves one free Dipp_3_SbO, showed clear evidence of exchange among desymmetrized signals of each of the species (arising from rotation about Sb–C bonds). There was, however, no evidence of exchange between the free and bound stibine oxide (Figure S33). Although we were consistently unsuccessful at isolating pure bulk samples of the Dipp_3_SbO·B­(C_6_F_5_)_3_ adduct, diffraction-quality crystals could be grown from a solution containing equimolar amounts of B­(C_6_F_5_)_3_ and Dipp_3_SbO (Figure 3). Crystallographic analysis confirmed the formation of the adduct Dipp_3_SbO·B­(C_6_F_5_)_3_, which featured an Sb–O bond length of 1.8947(14) Å and a B–O bond length of 1.512(3) Å.

**Figure 3:**
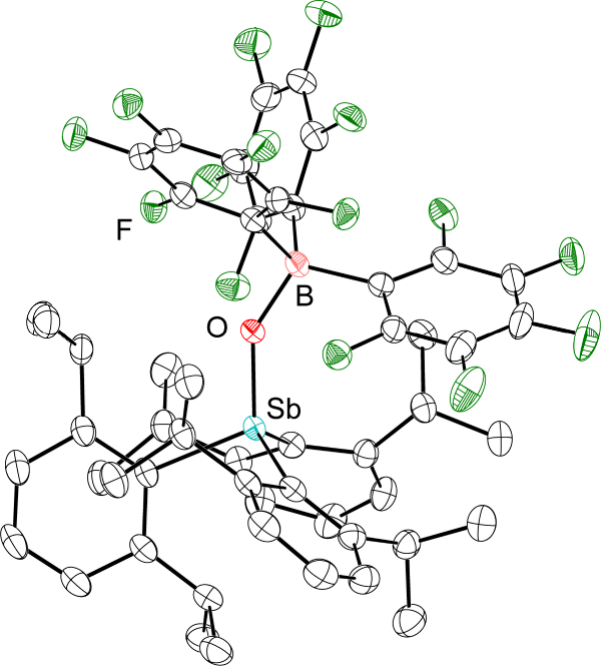
Thermal ellipsoid plots of Dipp_3_SbO·B­(C_6_F_5_)_3_. Ellipsoids are shown at the 50% probability level. H atoms are omitted for clarity. Color code: Sb light blue, B pink, O red, C black, F green.

### Synthesis of [(3,5-Me_2_Ph)_3_SbO]_*n*_ and (3,5-Me_2_Ph)_3_SbO·B­(C_6_F_5_)_3_

To further systematically assess the impact of sterics on the formation of R_3_SbO·B­(C_6_F_5_)_3_ adducts, we targeted the less encumbered (3,5-Me_2_Ph)_3_SbO·B­(C_6_F_5_)_3_. To access the corresponding stibine oxide, (3,5-Me_2_Ph)_3_Sb was oxidized with PhIO; the white solid that formed is insoluble in a wide range of solvents. The IR spectrum of the product matches that of the starting stibine except for additional bands at 682 and 487 cm^–1^ (Figures S41 and S42), which we assign to Sb–O stretching and C–Sb–O bending modes, respectively. From the supernatant of the (3,5-Me_2_Ph)_3_Sb oxidation reaction mixture, a small portion of diffraction-quality crystals could be grown. Crystallographic analysis showed them to comprise the dimeric [(3,5-Me_2_Ph)_3_SbO]_2_ (Figure 4). The molecule crystallized in space group *P*2_1_/*n* and resides on a crystallographic inversion center. The Sb···Sb distance is 3.1258(6) Å and the central Sb_2_O_2_ rhomb has side lengths of 2.0784(17) and 1.9356(15) Å, an O–Sb–O angle of 77.77(7)°, and an Sb–O–Sb angle of 102.23(7)°. The Sb center has a distorted trigonal bipyramidal geometry and the pseudoaxial Sb–O bond length is the longer of the two, consistent with the 3-center-4-electron bonding expected along the trigonal bipyramidal axis.

**Figure 4:**
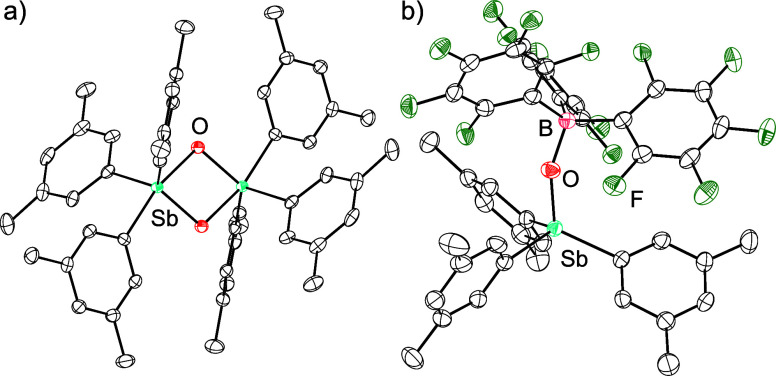
Thermal ellipsoid plots of (a) [(3,5-Me_2_Ph)_3_SbO]_2_ and (b) (3,5-Me_2_Ph)_3_SbO·B­(C_6_F_5_)_3_. Ellipsoids are shown at the 50% probability level. H atoms are omitted for clarity. Color code: Sb light blue, B pink, O red, C black, F green.

These results mirror those obtained upon oxidation of Ph_3_Sb,^28,29^ and we propose that the bulk product is a [(3,5-Me_2_Ph)_3_SbO]_*n*_ polymeric species analogous to (Ph_3_SbO)_*n*_ (Scheme 5).

**Scheme 5:**
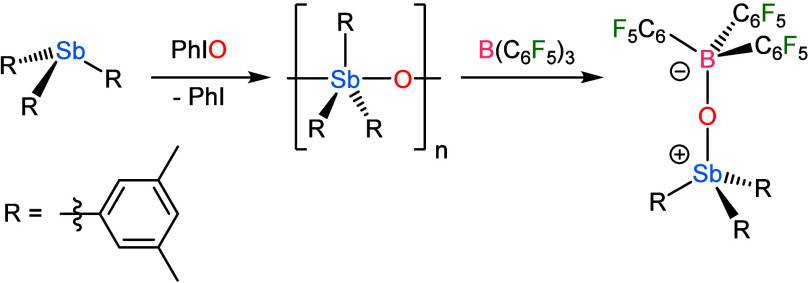
Synthesis of [(3,5-Me_2_Ph)_3_SbO]_*n*_ and Its Disaggregation with B­(C_6_F_5_)_3_

Suspension of crude [(3,5-Me_2_Ph)_3_SbO]_*n*_ in a solution of B­(C_6_F_5_)_3_ led to its gradual consumption and the formation of (3,5-Me_2_Ph)_3_SbO·B­(C_6_F_5_)_3_. This product displayed a ^11^B NMR signal at −0.98 ppm and sharp ^19^F NMR signals with Δδ_m,p_ = 4.5 ppm; features that are consistent with a tetrasubstituted B center. Both the ^1^H and ^13^C NMR spectra show only a single methyl resonance, indicating that, in contrast to the more sterically hindered Mes_3_SbO·B­(C_6_F_5_)_3_, rotation about the Sb–C bond is rapid on the NMR time scale at room temperature.

The X-ray crystal structure of (3,5-Me_2_Ph)_3_SbO·B­(C_6_F_5_)_3_ revealed the B–O bond length to be 1.487(4) Å, a value that is indistinguishable from that in Mes_3_SbO·B­(C_6_F_5_)_3_. Although there is a difference in the steric congestion of (3,5-Me_2_Ph)_3_SbO·B­(C_6_F_5_)_3_ and Mes_3_SbO·B­(C_6_F_5_)_3_, both feature a strong and nearly equivalent dative interaction between the stibine oxide and the borane. This trend holds upon extension to the previously reported and even less sterically encumbered adduct Ph_3_SbO·B­(C_6_F_5_)_3_,^17^ which has a B–O distance that is slightly longer (1.508(4) Å). In all three cases, a strong adduct forms with a B–O distance, consistent with the formation of a single bond.

### Structural Comparisons

The body of structural data presently collected permits valuable comparisons to be drawn, shedding light on the relative influence of donicity and steric bulk on pnictine oxide Lewis basicity.

The previously reported Ph_3_PnO·B­(C_6_F_5_)_3_ series showed a systematic trend of decreasing B–O bond lengths with increasing Pn atomic number (Table 1), which is consistent with the formation of an increasingly strong dative interaction between the Lewis basic pnictine oxide and the Lewis acidic borane. This trend continues in the Mes_3_PnO·B­(C_6_F_5_)_3_ series (Table 1), in that the B–O bond is shorter for the stibine oxide adduct than for the arsine oxide adduct; the phosphine oxide adduct was weakened enough that it existed only in dynamic equilibrium and could not be isolated (*vide supra*). This effect was exaggerated upon further increasing the steric bulk of the pnictine oxides: the only Dipp_3_PnO·B­(C_6_F_5_)_3_ species that could be observed, let alone isolated, was that formed by the stibine oxide.

**Table 1:** Crystallographically Determined Bond Lengths

	bond lengths (Å)		reference
	Pn–O	B–O	
Ph_3_PO·B(C_6_F_5_)_3_	1.497(2)	1.538(3)	^6^
Ph_3_AsO·B(C_6_F_5_)_3_	1.698(2)	1.521(3)	^17^
Ph_3_SbO·B(C_6_F_5_)_3_	1.877(2)	1.508(4)	^17^
Mes_3_AsO·B(C_6_F_5_)_3_	1.7094(15)	1.533(3)	this work
Mes_3_SbO·B(C_6_F_5_)_3_	1.884(3)	1.488(5)	this work
Dipp_3_SbO·B(C_6_F_5_)_3_	1.8947(14)	1.512(3)	this work
(3,5-Me_2_Ph)_3_SbO·B(C_6_F_5_)_3_	1.864(2)	1.487(4)	this work
Mes_3_SbO·BPh_3_	1.829(3)	1.546(5)	this work

Comparison of Ph_3_AsO·B­(C_6_F_5_)_3_ and Mes_3_AsO·B­(C_6_F_5_)_3_ highlights that the increased size of the R groups bound to the As atom lengthens and weakens the B–O bond. The weaker B–O interaction coincides with a shorter/stronger As–O bond. Similarly, the series of R_3_SbO·B­(C_6_F_5_)_3_ compounds can be compared for R = Ph, 3,5-Me_2_Ph, Mes, and Dipp. The most sterically encumbered member of the series, Dipp_3_SbO·B­(C_6_F_5_)_3_, indeed has the longest B–O bond.

### Computational Investigation

The spectroscopic and X-ray structural data described above present a series of results that are consistent with stibine oxides having a greater capacity to form adducts with Lewis acids and that the adducts that form are stronger as compared to the lighter pnictine oxide congeners. We obtained further insight into these reactions computationally. We first computed the free energies for B­(C_6_F_5_)_3_ reacting with Mes_3_PnO or Dipp_3_PnO (Pn = P, As, Sb) (Table 2). For all adducts, there are stationary points in which a B–O bond persists. There is, however, a significant increase in the magnitude of Δ*G*_rxn_ as the atomic number of the pnictogen increases. At the level of theory that was computationally tractable for these molecules (all substituents need to remain to capture steric effects), we do not anticipate accurate numerical agreement between theoretical and experimental thermochemistry. Nevertheless, the trends present in the computational data are informative. In both series, |Δ*G*_rxn_| increases from P to Sb, with the increase being more dramatic for the Dipp_3_PnO compounds. The Δ*G*_rxn_ for Mes_3_PO is approximately the same as that for Dipp_3_AsO and any reactions computed to have a Δ*G*_rxn_ at this level or less negative did not afford isolable adducts; all those with Δ*G*_rxn_ values more negative than this threshold did afford isolable adducts. The influence of the steric bulk was clearly evident in the relative values of Δ*G*_rxn_ for pairs of compounds with the same Pn atom but different R groups. The bulkier Dipp groups unilaterally decreased the favorability of the reaction, but the effect size increased with decreasing atomic number of the Pn atom. For Sb, the Lewis basicity of the R_3_SbO motif was able to overcome even the notable steric bulk of the Dipp groups.

**Table 2:** Computational Free Energies of Reaction and Bond Critical Point Electron Densities

	Δ*G*_rxn_ (kJ mol^–1^)	ρ_bcp_ (a.u)
Mes_3_PO·B(C_6_F_5_)_3_	–75	0.108
Mes_3_AsO·B(C_6_F_5_)_3_	–118	0.119
Mes_3_SbO·B(C_6_F_5_)_3_	–165	0.127
Dipp_3_PO·B(C_6_F_5_)_3_	–30	0.103
Dipp_3_AsO·B(C_6_F_5_)_3_	–82	0.118
Dipp_3_SbO·B(C_6_F_5_)_3_	–129	0.125

Analysis of the topology of the electron density of the resulting adducts revealed that there were no significant differences in the nature of the Lewis acid–base interactions that occurred as either R or Pn was changed. That is, the overall shapes of the electron density (ρ) and the Laplacian of the electron density (∇^2^ρ) along the lengths of the bond paths did not change (Figure 5). The importance of analyzing real space functions along the length of the bond path when studying polar covalent bonds has been previously highlighted.^30^ Across both series, there was a subtle trend whereby the value of ρ at the bond critical point (ρ_bcp_; Table 2) increased with increasing atomic number of the pnictogen. Likewise, both local minima in the Laplacian in the O–B interatomic valence region were progressively more negative with increasing pnictogen atomic number. These trends are subtle, but consistent with the ability of the stibine oxide to form a stronger interaction with the borane. The capacity of stibine oxides to form stronger Lewis acid–base interactions than lighter congeners will stem in part from the increased ability of the O-based lone pairs to donate to an acceptor, as we have shown earlier with a sterically unencumbered Lewis acid, H^+^.^21^ In the case of the more sterically hindered Lewis acids investigated here, the greater propensity for adduct formation will also have a contribution from the increase in the length of the Pn–O bond as the atomic number of the Pn atom increases (Table 1), which decreases the steric strain in the adduct that forms.

**Figure 5:**
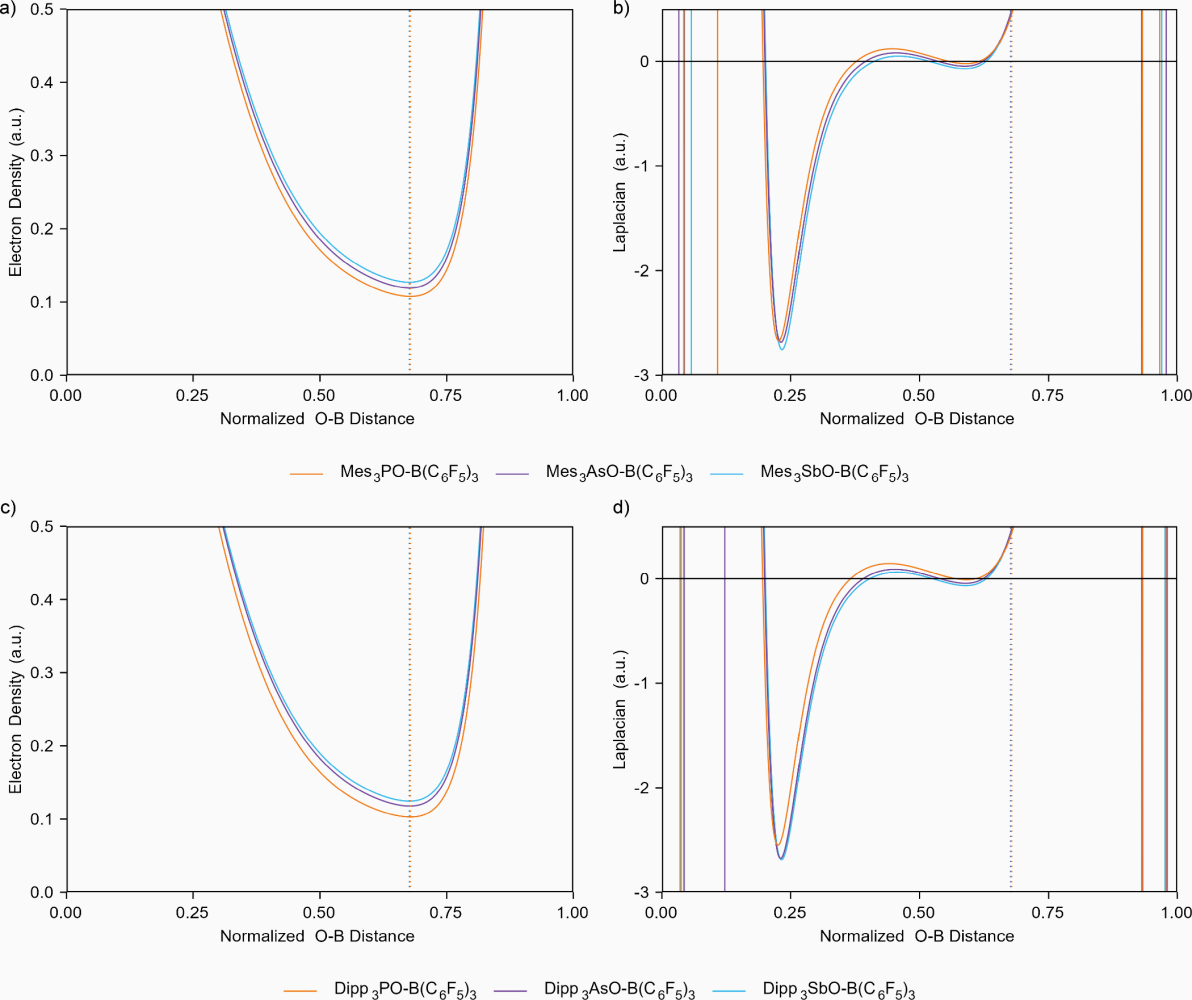
Analysis of (a) the electron density and (b) the Laplacian of the electron density along the O–B bond path for the Mes_3_PnO·B­(C_6_F_5_)_3_ series where Pn = P, As, and Sb. Dotted vertical lines indicate the position of the bond critical point. (c, d) Analogous information for the Dipp_3_PnO·B­(C_6_F_5_)_3_ series.

## Conclusions

The unique electronic structure of the phosphoryl group affords phosphine oxides with significant chemical stability while still allowing them to engage in Lewis basic reactivity. The stability of the phosphoryl group derives from dative back-bonding interactions of the lone pairs on the terminal oxygen atom, which attenuates their ability to engage in donation to an external Lewis acid. In stibine oxides, the stiboryl group also features back-bonding from the oxygen atom, but to a much lesser extent. The pnictogen–oxygen bond is consequently weaker, but the Lewis basicity of the functional group is correspondingly enhanced. Here, we have demonstrated that this enhanced Lewis basicity can overcome levels of steric hindrance that otherwise prevent the formation of stable Lewis acid–base adducts for analogous phosphine oxides and arsine oxides.

## Experimental Section

### General Methods

Reagents and solvents were purchased from commercial vendors and used as received, unless otherwise specified. Triphenylborane, tris­(3,5-dimethylphenyl)­stibine, trimesitylstibine oxide, trimesitylphosphine oxide, tris­(2,6-diisopropylphenyl)­phosphine oxide, tris­(2,6-diisopropylphenyl)­arsine oxide, and tris­(2,6-diisopropylphenyl)­stibine oxide were synthesized according to literature protocols.^12,18,31–33^ All reactions were performed under a N_2_ atmosphere in an OMNI-lab glovebox or on a dual-manifold Schlenk line. All solvents were dried over 3 Å molecular sieves. NMR spectra were collected by using a Bruker Avance III HD 500 spectrometer equipped with a multinuclear Smart Probe. Unless otherwise specified, NMR experiments were performed at room temperature. Signals in the ^1^H and ^13^C NMR spectra are reported in ppm as chemical shifts from tetramethylsilane and were referenced using the CHCl_3_ (^1^H, 7.26 ppm) and CDCl_3_ (^13^C, 77.16 ppm) solvent signals. The frequencies of ^11^B NMR signals are reported in ppm as chemical shifts from BF_3_·OEt_2_ (referenced to an external sample of BF_3_·OEt_2_). The frequencies of ^19^F NMR signals are reported in ppm as chemical shifts from CFCl_3_ (referenced to an external sample of BF_3_·OEt_2_ at –152.8 ppm). ^31^P NMR signals are reported as chemical shifts from 85% H_3_PO_4_ (referenced to an external solution of triphenylphosphine in CDCl_3_ at −5.35 ppm). Infrared (IR) spectra were collected on KBr pellets by using a PerkinElmer Spectrum One FT-IR spectrometer. Elemental analysis was performed at the UC Berkeley College of Chemistry Microanalytical Facility. NMR data were processed using Mestrenova (version 12.0.2).

### Synthesis of (Trimesitylstibine Oxide)·Triethoxyalane Adduct (Mes_3_SbO·Al­(OEt)_3_)

A solution of Mes_3_SbO (70 mg, 0.14 mmol) in DCM (1 mL) was added to a suspension of Al­(OEt)_3_ (23 mg, 0.14 mmol) in DCM (3 mL). The suspension was allowed to stir overnight. After 12 h, the mixture was passed through a Celite pad, with an additional aliquot of DCM (1 mL) used for quantitative transfer. The clarified filtrate was stripped of the solvent to yield a white powder. The solid was washed with pentane (3 × 0.5 mL) before being dried under vacuum. Yield: 83 mg (89%). Crystals suitable for X-ray diffraction were grown from the slow diffusion of pentane into a chloroform solution of the adduct. Found: C, 59.98%; H, 7.26%. Calc. for C_33_H_48_O_4_AlSb: C, 60.28%; H, 7.36%. ^1^H NMR (500 MHz, CDCl_3_) δ: 6.95 (s, 6H), 3.63 (q, 6H), 2.47 (s, 18H), 2.30 (s, 9H), 0.99 (q, 9H). ^13^C­{^1^H} NMR (125 MHz, CDCl_3_) δ: 143.65, 142.96, 133.72, 130.94, 57.68, 24.21, 21.26, 21.01.

### NMR Spectroscopy of Mes_3_SbO/B­(OEt)_3_

Mixtures of Mes_3_SbO and B­(OEt)_3_ in CDCl_3_ were prepared in ratios of 1:1, 1:5, and 1:10. ^1^H and ^11^B­{^1^H} NMR spectra were acquired at room temperature (Figures S3 and S4).

### Synthesis of (Trimesitylstibine Oxide)·Triphenylborane Adduct (Mes_3_SbO·BPh_3_)

A solution of Mes_3_SbO (50 mg, 0.10 mmol) in toluene (3 mL) was added to a solution of BPh_3_ (24 mg, 0.10 mmol) in toluene (3 mL). The mixture was allowed to stand for 1 h, during which time a white, microcrystalline solid precipitated from the solution. The supernatant was decanted, and the precipitate was washed with pentane (3 × 0.5 mL) before being dried under vacuum. Yield: 65 mg (87%). The insolubility of the product in a variety of solvents precluded NMR spectroscopic characterization. Crystals suitable for X-ray diffraction were grown by layering a toluene solution of Mes_3_SbO on top of a toluene solution of BPh_3_. Powder X-ray diffraction was used to confirm the purity of the bulk product (Figure S5).

### Synthesis of (Trimesitylstibine Oxide)·Tris­(pentafluorophenyl)­borane Adduct Chloroform Solvate (Mes_3_SbO·B­(C_6_F_5_)_3_·CHCl_3_)

A solution of Mes_3_SbO (60 mg, 0.12 mmol) in DCM (2 mL) was added to a solution of B­(C_6_F_5_)_3_ (62 mg, 0.12 mmol) in DCM (2 mL), and the mixture was allowed to stand for 5 min before being stripped of solvent to yield a crude yellow oil. The residue was extracted with chloroform (3 mL) and layered under pentane, yielding colorless crystals after 36 h. Yield: 71 mg (52%). Crystals suitable for X-ray diffraction were grown from the slow diffusion of pentane into a solution of the product. ^1^H NMR (500 MHz, CDCl_3_) δ: 6.91 (s, 6H), 2.5–2.1 (br, 18H), 2.5–2.1 (br, 9H). ^11^B­{^1^H} NMR (160 MHz, CDCl_3_) δ: −0.75. ^19^F­{^1^H} NMR (470 MHz, CDCl_3_) δ: −133.21 (d, 6F), −160.21 (t, 3F), −165.35 (t, 6F). ^13^C­{^1^H} NMR (125 MHz, CDCl_3_) δ: 143.16, 134.22, 131.14, 23.05, 21.06. Powder X-ray diffraction was used to confirm the purity of the bulk product (Figure S11).

### Synthesis of Trimesitylarsine (Mes_3_As)

Magnesium turnings (0.57 g, 23.45 mmol) were suspended in THF (15 mL) with 1,2-dibromoethane (0.507 mL, 5.862 mmol) at 0 °C. The mixture was brought to reflux and then cooled to room temperature, and 2-bromomesitylene (2.652 mL, 17.58 mmol) was added dropwise. The reaction mixture was then refluxed until the magnesium was fully consumed (6 h). AsCl_3_ (0.492 g, 5.862 mmol) was then added dropwise into the yellow solution of mesitylmagnesium bromide at room temperature. The reaction mixture was then refluxed for 14 h. The resulting mixture was cooled to room temperature, diluted with diethyl ether (200 mL), and washed with water (200 mL). The aqueous phase was back-extracted with diethyl ether (2 × 50 mL), and the organic phases were combined, washed with water (3 × 100 mL), and washed with brine (1 × 100 mL). The organic phase was dried over anhydrous sodium sulfate for 30 min and stripped of solvent under reduced pressure to yield a crude yellow oil. Acetonitrile was added dropwise with sonication to afford a white powder that was collected via vacuum filtration and dried in vacuo. Yield: 1.4 g (56%). Analytical data match those previously reported for this compound.^34^ ^1^H NMR (500 MHz, CDCl_3_) δ: 6.78 (s, 3H), 2.25 (s, 9 H), 2.14 (s, 18).

### Synthesis of Trimesitylarsine Oxide (Mes_3_AsO)

Mes_3_As (400 mg, 0.924 mmol) was dissolved in DCM (10 mL). Hydrogen peroxide (9.249 mL, 4.624 mmol, 50% aqueous solution) was added dropwise, and the heterogeneous reaction was allowed to stir for 1 h. The reaction was quenched by the addition of a saturated aqueous NaHCO_3_ solution (100 mL) and a 5% aqueous solution of Na_2_S_2_O_3_ (200 mL). The reaction was diluted with additional DCM (50 mL) and the organic phase was separated. The aqueous phase was back-extracted with DCM (2 × 150 mL), and the organic phases were combined, washed with water (3 × 100 mL), and washed with brine (1 × 100 mL). The organic phase was dried over anhydrous sodium sulfate for 30 min and stripped of solvent under reduced pressure to yield a transparent oil. The oil was taken up in minimal hexanes (10 mL) and placed in a freezer overnight to afford colorless crystals. Yield: 369 mg (89%). ^1^H NMR (500 MHz, CDCl_3_) δ: 6.84 (s, 6H), 2.33 (br, 18H), 2.27 (s, 9H). ^13^C­{^1^H} NMR (125 MHz, CDCl_3_) δ: 141.85, 140.84, 135.53, 131.15, 22.98, 21.09. Powder X-ray diffraction data from freshly prepared bulk solid agreed with the simulated powder diffractogram generated from the crystal structure (Figure S16).

### Synthesis of (Trimesitylarsine Oxide)·Tris­(pentafluorophenyl)­borane Adduct Chloroform Solvate (Mes_3_AsO·B(C_6_F_5_)_3_·CH_2_Cl_2_)

A solution of Mes_3_AsO (30 mg, 0.067 mmol) in DCM (2 mL) was combined with a solution of B­(C_6_F_5_)_3_ (34 mg, 0.067 mmol) in DCM (2 mL). The reaction mixture was allowed to stand for 5 min and then passed through a Celite pad, using an additional aliquot of DCM (1 mL) for quantitative transfer. Pentane (3 mL) was added to the filtrate, which was concentrated under reduced pressure until it was cloudy. The solution was allowed to stand at room temperature to afford colorless plates. The supernatant was decanted, and the crystals were washed with minimal pentane. Yield: 24 mg (35%). ^1^H NMR (500 MHz, CDCl_3_) δ: 6.86 (s, 3H), 6.80 (s, 3H), 2.55 (s, 9H), 2.27 (s, 9H), 2.05 (s, 9H). ^11^B­{^1^H} NMR (160 MHz, CDCl_3_) δ: −0.27. ^19^F­{^1^H} NMR (470 MHz, CDCl_3_) δ: −131.82 (d, 6F), −159.67 (t, 3F), −165.53 (t, 6F). ^13^C­{^1^H} NMR (125 MHz, CDCl_3_) δ: 144.52, 142.97, 140.59, 132.06, 131.72, 131.67, 22.50, 20.90. Powder X-ray diffraction data from freshly prepared bulk solid agreed with the simulated powder diffractogram generated from the crystal structure (Figure S21).

### NMR Spectroscopy of Mes_3_PO/B­(C_6_F_5_)_3_

A 1:1 mixture of Mes_3_PO and B­(C_6_F_5_)_3_ in CDCl_3_ was prepared and ^1^H and ^31^P­{^1^H} NMR spectra were acquired at 20, 10, 0, −10, and −20 °C (Figures S25–S28). Reference spectra of Mes_3_PO alone at room temperature and −20 °C were also collected (Figures S22–S24).

### Synthesis of (Tris­(2,6-diisopropylphenyl)­stibine oxide)·Tris­(pentafluorophenyl)­borane Adduct (Dipp_3_SbO·B­(C_6_F_5_)_3_)

A solution of Dipp_3_SbO (60 mg, 0.12 mmol) in DCM (2 mL) was combined with a solution of B­(C_6_F_5_)_3_ (62 mg, 0.12 mmol) in DCM (2 mL). The solution was allowed to stand for 5 min. The reaction mixture was stripped of solvent to yield a crude yellow oil, which was taken up in pentane (3 mL) and passed through a Celite pad with an additional aliquot of pentane (1 mL) used for quantitative transfer. The clarified filtrate was stripped of the solvent to yield a yellow residue. Yield: 33 mg (31%). Crystals suitable for X-ray diffraction were grown by slow concentration of a DCM/cyclohexane solution of the product. ^1^H NMR (500 MHz, CDCl_3_) δ: 7.41 (t, 3H), 7.31 (d, 3H), 7.19 (d, 3H), 3.33 (br, 3H), 2.45 (br, 3H), 0.92 (d, 9H), 0.87 (d, 9H), 0.68 (d, 9H) 0.58 (d, 9H). ^11^B­{^1^H} NMR (160 MHz, CDCl_3_) δ: −0.87. ^19^F­{^1^H} NMR (470 MHz, CDCl_3_) δ: −128.17, −129.79 (b, 6F), −159.84­(t, 3F), −165.17­(t, 6F). The compound was unstable both in solution and in the solid-state preventing successful elemental analysis.

### NMR Spectroscopy of Dipp_3_AsO/B­(C_6_F_5_)_3_ and Dipp_3_PO/B­(C_6_F_5_)_3_

Mixtures of B­(C_6_F_5_)_3_ with Dipp_3_PO and Dipp_3_AsO in CDCl_3_ (1:1 ratio of borane and pnictine oxide) were prepared, and ^1^H, ^11^B­{^1^H}, ^19^F­{^1^H}, and ^31^P­{^1^H} NMR spectra were acquired (Figures S34–S40).

### Synthesis of Tris­(3,5-dimethylphenyl)­stibine Oxide ((3,5-Me_2_Ph)_3_SbO)_*n*_

A solution of (3,5-Me_2_Ph)_3_Sb (98 mg, 0.22 mmol) in DCM (10 mL) was added to a suspension of iodosobenzene (49 mg, 0.22 mmol) in DCM (2 mL). The resulting yellow suspension was allowed to stir at room temperature. After 1.5 h, the iodosobenzene had been consumed, and a white, amorphous solid had precipitated from the reaction mixture. The supernatant was decanted, and the product was washed with pentane (3 × 1.5 mL) before being dried under vacuum. Yield: 86 mg (85%). Satisfactory elemental analysis could not be obtained; a problem that was also encountered with the previously reported (Ph_3_SbO)_*n*_. Crystals of the dimeric species ((3,5-Me_2_Ph)_3_SbO)_2_ suitable for X-ray diffraction were grown from the slow concentration of the decanted supernatant. IR (KBr, cm^–1^): 682 (ν_Sb–O, stretch_), 487 (δ_C–Sb–O, bend_).

### Synthesis of (Tris­(3,5-dimethylphenyl)­stibine oxide)·Tris­(pentafluorophenyl)­borane Adduct ((3,5-Me_2_Ph)_3_SbO·B­(C_6_F_5_)_3_)

A solution of ((3,5-Me_2_Ph)_3_SbO)_*n*_ (61 mg, 0.14 mequiv of monomer) in DCM (2 mL) was combined with a solution of B­(C_6_F_5_)_3_ (69 mg, 0.13 mmol) in DCM (2 mL). The solution was allowed to stand for 5 min. The solution was passed through a Celite pad with an additional aliquot of DCM (1 mL) used for quantitative transfer. The clarified filtrate was stripped of solvent and the solid was washed with pentane (3 × 1 mL) to yield a white powder. Yield: 54 mg (84%). Crystals suitable for X-ray diffraction were grown from the slow diffusion of pentane into a chloroform solution of the product. ^1^H NMR (500 MHz, CDCl_3_) δ: 7.27 (s, 3H), 7.11 (s, 6H), 2.33 (s, 18H). ^11^B­{^1^H} NMR (160 MHz, CDCl_3_) δ: −0.98. ^19^F­{^1^H} NMR (470 MHz, CDCl_3_) δ: −132.88 (d, 6F), −160.62 (t, 3F), −165.07 (t, 6F). ^13^C­{^1^H} NMR (125 MHz, CDCl_3_) δ: 140.74, 135.52, 131.52, 126.41, 21.45. Powder X-ray diffraction data from freshly prepared bulk solids agreed with the simulated powder diffractogram generated from the crystal structure (Figure S47).

### X-ray Crystallography

Crystals of Mes_3_SbO·Al­(OEt)_3_, Mes_3_SbO·BPh_3_, Mes_3_SbO·B­(C_6_F_5_)_3_·CHCl_3_, Mes_3_AsO, Mes_3_AsO·B­(C_6_F_5_)_3_·CH_2_Cl_2_, Dipp_3_SbO·B­(C_6_F_5_)_3_, ((3,5-Me_2_Ph)_3_SbO)_2_, and (3,5-Me_2_Ph)_3_SbO·B­(C_6_F_5_)_3_ were grown as described above, selected under a microscope, loaded onto a MiTeGen polyimide sample loop using Type NVH Cargille immersion oil, and mounted onto a Rigaku XtaLAB Synergy-S single-crystal X-ray diffractometer. Each crystal was cooled to 100 K under a stream of nitrogen. Diffraction of Cu Kα radiation from a PhotonJet-S microfocus source was detected by using a HyPix6000HE hybrid photon counting detector. Screening, indexing, data collection, and data processing were performed with CrysAlis^Pro^.^35^ The structures were solved using SHELXT and refined using SHELXL following established strategies.^36–38^ All non-H atoms were refined anisotropically. C-bound H atoms were placed at calculated positions and refined with a riding model and coupled isotropic displacement parameters (1.2 × U_eq_ for nonmethyl C–H atoms and 1.5 × U_eq_ for methyl groups).

### Powder X-ray Diffraction

Bulk samples of Mes_3_SbO·BPh_3_, Mes_3_AsO, Mes_3_AsO·B­(C_6_F_5_)_3_·(CH_2_Cl_2_), (3,5-Me_2_Ph)_3_SbO·B(C_6_F_5_)_3_, and Mes_3_SbO·B­(C_6_F_5_)_3_·(CHCl_3_) were ground using an agate mortar and pestle. The fine powders were each loaded onto a MiTeGen polyimide sample loop using Type NVH Cargille immersion oil and mounted onto a Rigaku XtaLAB Synergy-S single-crystal X-ray diffractometer. The powder was cooled to 100 K under a stream of nitrogen. The diffraction of Cu Kα radiation was collected while the sample underwent a Gandolfi scan. Data collection and processing were performed using CrysAlis^Pro^.^35^ Simulated powder diffractograms were generated from experimental crystal structures using Mercury.^39,40^ These simulated diffractograms were compared with the experimentally measured powder diffractograms.

### Computational Experiments

All geometry optimizations and frequency calculations were performed in the gas phase using ORCA 5.0.1.^41–43^ Geometry optimizations and frequency calculations were performed using crystallographic input coordinates at the BP86/def2-SVP level of theory, with the RI approximation and def2/J auxiliary basis set.^44–47^ For all calculations, dispersion corrections were applied using Grimme’s D3 method with Becke-Johnson damping.^48^ Electron densities used for topological analysis were generated at the DKH-PBE0/old-DKH-TZVPP level of theory using the BP86/def2-SVP-optimized coordinates, with the RIJCOSX approximation and SARC/J auxiliary basis set.^49–54^ Topological analyses were performed using MultiWFN.^55,56^

## Supplementary Material





## Notes

NMR and IR spectra, crystallographic refinement tables, thermal ellipsoid plots, and powder diffractograms (PDF)
Computationally optimized Cartesian coordinates (XYZ)
