# Supplementary material for: Enhanced Stibine Oxide Lewis Basicity Overcomes Steric Frustration
Source: Organometallics. 2025 Sep 9;44(18):2074–82. doi: 10.1021/acs.organomet.5c00212 (PMC12458979; doi:10.1021/acs.organomet.5c00212)
Supplement: Supplementary file 1 [file om5c00212_si_001.pdf]

Supporting Information for

Enhanced Stibine Oxide Lewis Basicity Overcomes Steric Frustration

Addis Getahun[a], *John S. Wenger*[a] *and Timothy C. Johnstone**[a]

[a] Department of Chemistry and Biochemistry, University of California Santa Cruz, Santa Cruz, California 95064, United States.

CONTENTS

	Page
Figures S1-S2: ^{1}H and $^{13}C\{^{1}H\}$ NMR spectra of $Mes_3SbO\cdot Al(OEt)_3$	S3
Figures S3-S4: ^{1}H and $^{11}B\{^{1}H\}$ NMR spectra of mixtures of Mes_3SbO and $B(OEt)_3$	S4
Figure S5: Simulated/experimental powder X-ray diffractograms of $Mes_3SbO\cdot BPh_3$	S5
Figures S6-S10: ^{1}H, $^{11}B\{^{1}H\}$, $^{19}F\{^{1}H\}$, and $^{13}C\{^{1}H\}$ NMR spectra of $Mes_3SbO\cdot B(C_6F_5)_3\cdot(CHCl_3)$	S6
Figures S11: Simulated/experimental powder X-ray diffractograms of $Mes_3SbO\cdot B(C_6F_5)_3\cdot(CHCl_3)$	S8
Figure S12: ^{1}H NMR spectrum of Mes_3As	S9
Figure S13-S15: ^{1}H and $^{13}C\{^{1}H\}$ NMR spectra of Mes_3AsO	S9
Figures S16: Simulated/experimental powder X-ray diffractograms of Mes_3AsO	S11
Figure S17-S20: ^{1}H, $^{11}B\{^{1}H\}$, $^{19}F\{^{1}H\}$, and $^{13}C\{^{1}H\}$ NMR spectra of $Mes_3AsO\cdot B(C_6F_5)_3$	S11
Figures S21: Simulated/experimental powder X-ray diffractograms of $Mes_3AsO\cdot B(C_6F_5)_3\cdot CH_2Cl_2$	S13
Figures S22-S24: ^{1}H and $^{31}P\{^{1}H\}$ NMR spectra of Mes_3PO	S14
Figures S25-S26: ^{1}H and $^{31}P\{^{1}H\}$ NMR spectra of Mes_3PO and $Mes_3PO\cdot B(C_6F_5)_3$	S15
Figures S27-S28: ^{1}H and $^{31}P\{^{1}H\}$ NMR spectra of $Mes_3PO/B(C_6F_5)_3$	S16
Figures S29-S33: ^{1}H, $^{11}B\{^{1}H\}$, $^{19}F\{^{1}H\}$, $^{13}C\{^{1}H\}$, and ^{1}H EXSY NMR spectra of $Dipp_3SbO\cdot B(C_6F_5)_3$	S17
Figures S34-S36: ^{1}H, $^{11}B\{^{1}H\}$, and $^{19}F\{^{1}H\}$ NMR spectra of $Dipp_3AsO/B(C_6F_5)_3$	S20
Figures S37-S40: ^{1}H, $^{11}B\{^{1}H\}$, $^{19}F\{^{1}H\}$, $^{31}P\{^{1}H\}$ NMR spectra of $Dipp_3PO/B(C_6F_5)_3$	S22
Figures S41: IR spectrum (KBr pellet) of $(3,5\text{-}Me_2Ph)_3Sb$	S23
Figures S42: IR spectrum (KBr pellet) of $[(3,5\text{-}Me_2Ph)_3SbO]_n$	S24
Figures S43-S46: ^{1}H, $^{11}B\{^{1}H\}$, $^{19}F\{^{1}H\}$, and $^{13}C\{^{1}H\}$ NMR spectra of $(3,5\text{-}Me_2Ph)_3SbO\cdot B(C_6F_5)_3$	S24
Figure S47: Simulated/experimental powder X-ray diffractograms of $(3,5\text{-}Me_2Ph)_3SbO\cdot B(C_6F_5)_3$	S26
Figures S48-S55: Thermal ellipsoid plots of crystal structures	S27
Tables S1-S2: Crystallographic parameters	S31

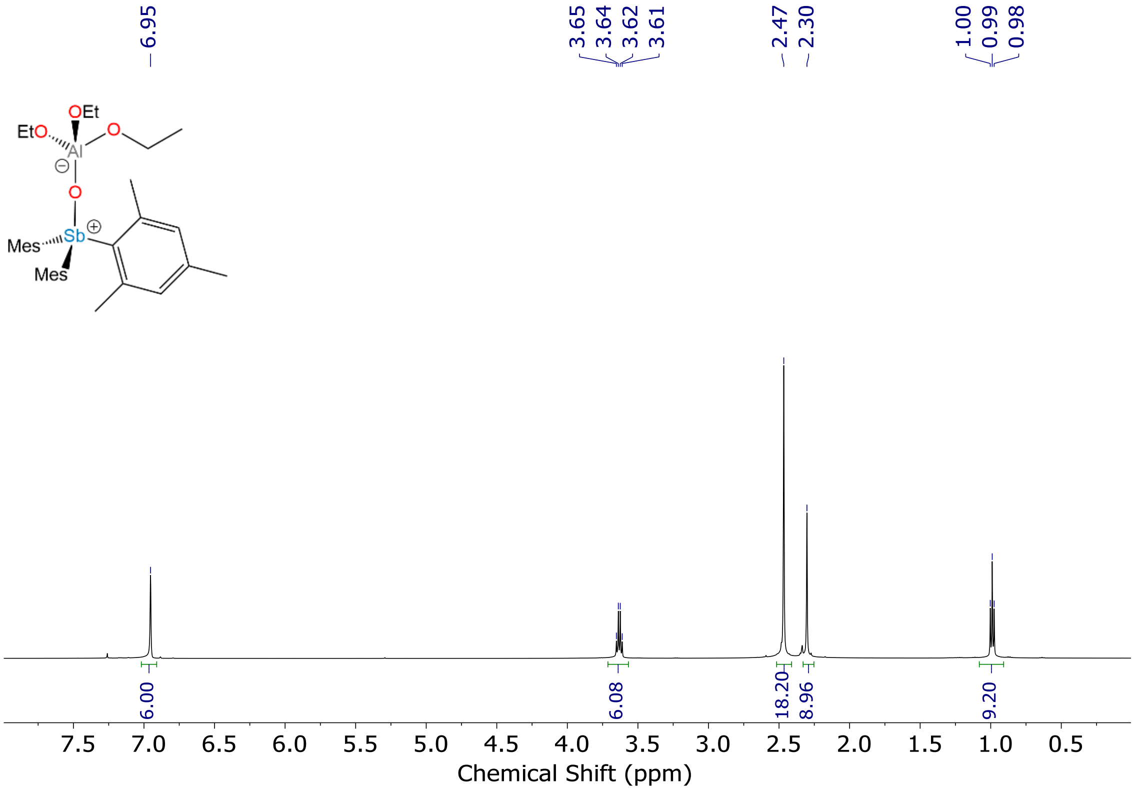

Figure S1. ^{1}H NMR spectrum ($CDCl_3$, 500 MHz) of $Mes_3SbO{\cdot}Al(OEt)_3$.

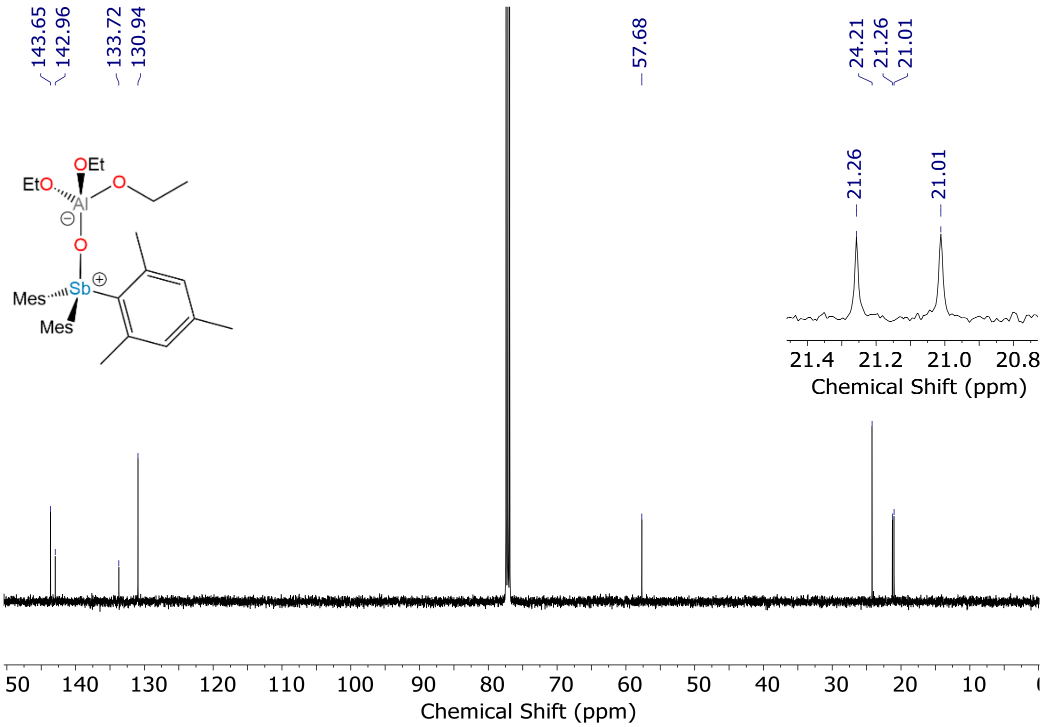

Figure S2. $^{13}C\{^{1}H\}$ NMR spectrum ($CDCl_3$, 125 MHz) of $Mes_3SbO{\cdot}Al(OEt)_3$.

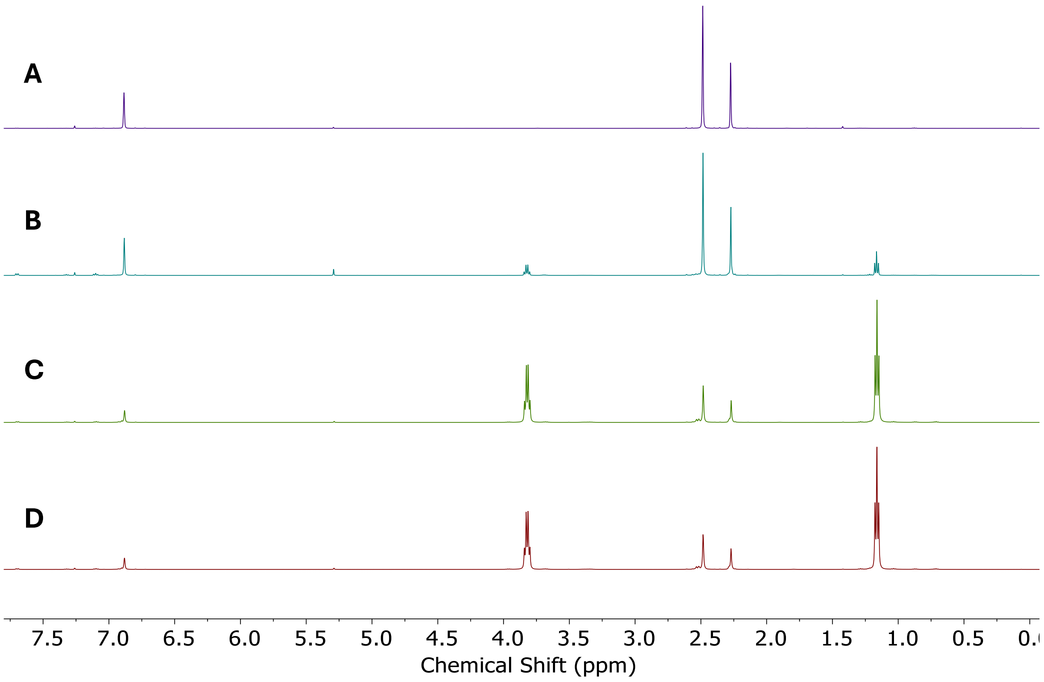

Figure S3. *In situ* ^{1}H NMR spectrum ($CDCl_3$, 500 MHz) of Mes_3SbO and $B(OEt)_3$, in which the concentration of $B(OEt)_3$ is increasing. (A) Mes_3SbO. (B) Mes_3SbO and 1 equivalent of $B(OEt)_3$. (C) Mes_3SbO and 5 equivalents of $B(OEt)_3$. (D) Mes_3SbO and 10 equivalents of $B(OEt)_3$.

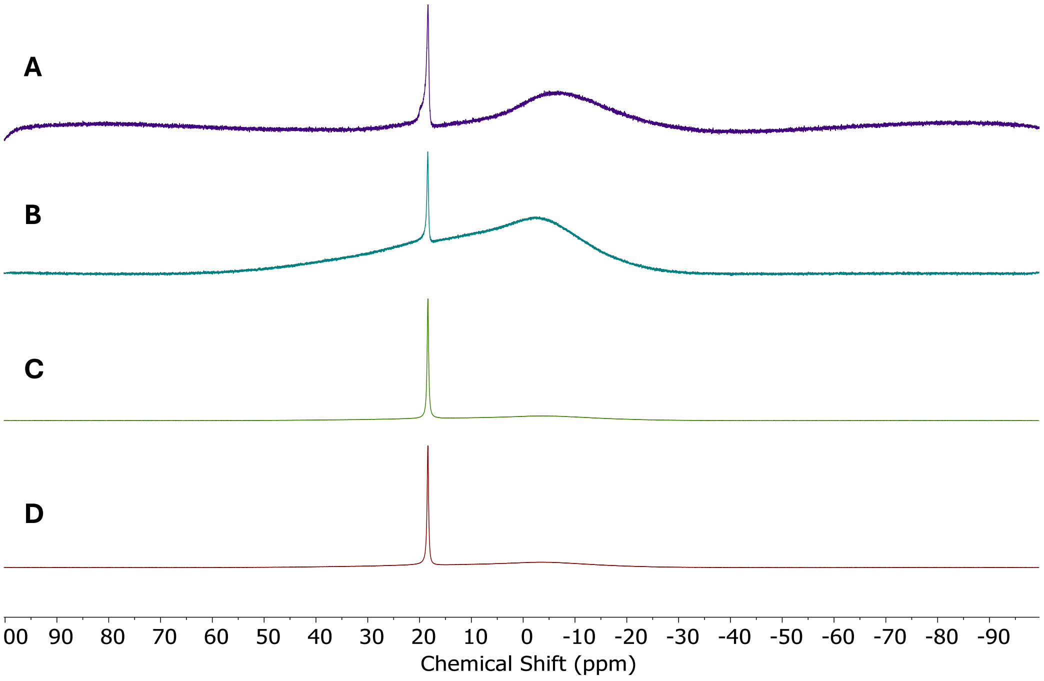

Figure S4. *In situ* $^{11}B\{^{1}H\}$ NMR spectrum ($CDCl_3$, 160 MHz) of Mes_3SbO and $B(OEt)_3$, in which the concentration of $B(OEt)_3$ is increasing. (A) $B(OEt)_3$. (B) Mes_3SbO and 1 equivalent of $B(OEt)_3$. (C) Mes_3SbO and 5 equivalent of $B(OEt)_3$. (D) Mes_3SbO and 10 equivalent of $B(OEt)_3$.

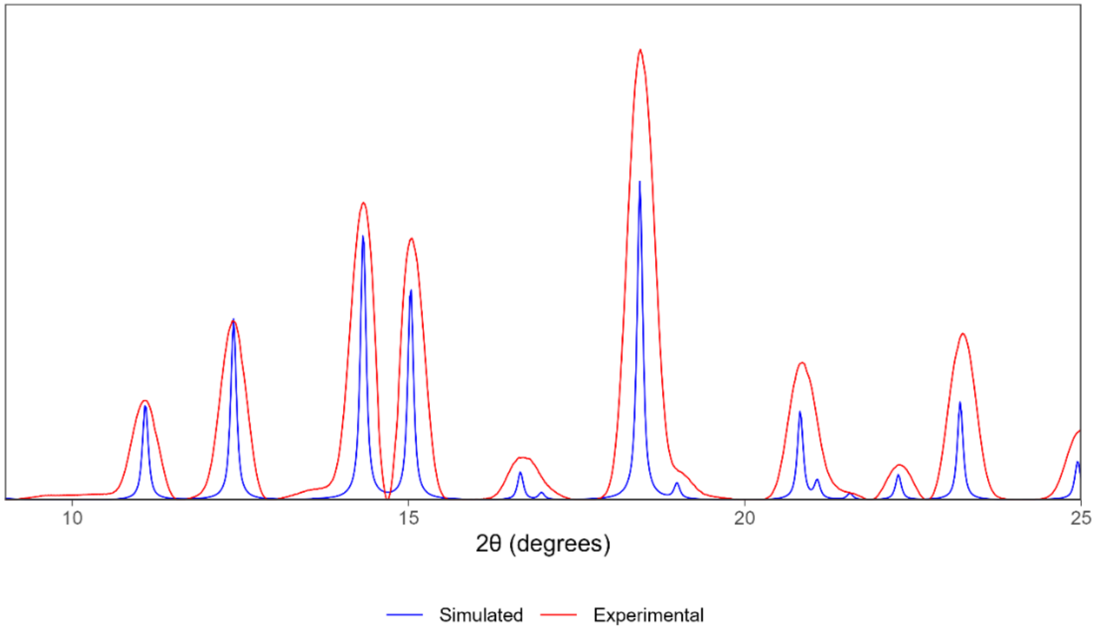

Figure S5. Simulated and experimental powder X-ray diffractograms of $Mes_3SbO{\cdot}BPh_3$.

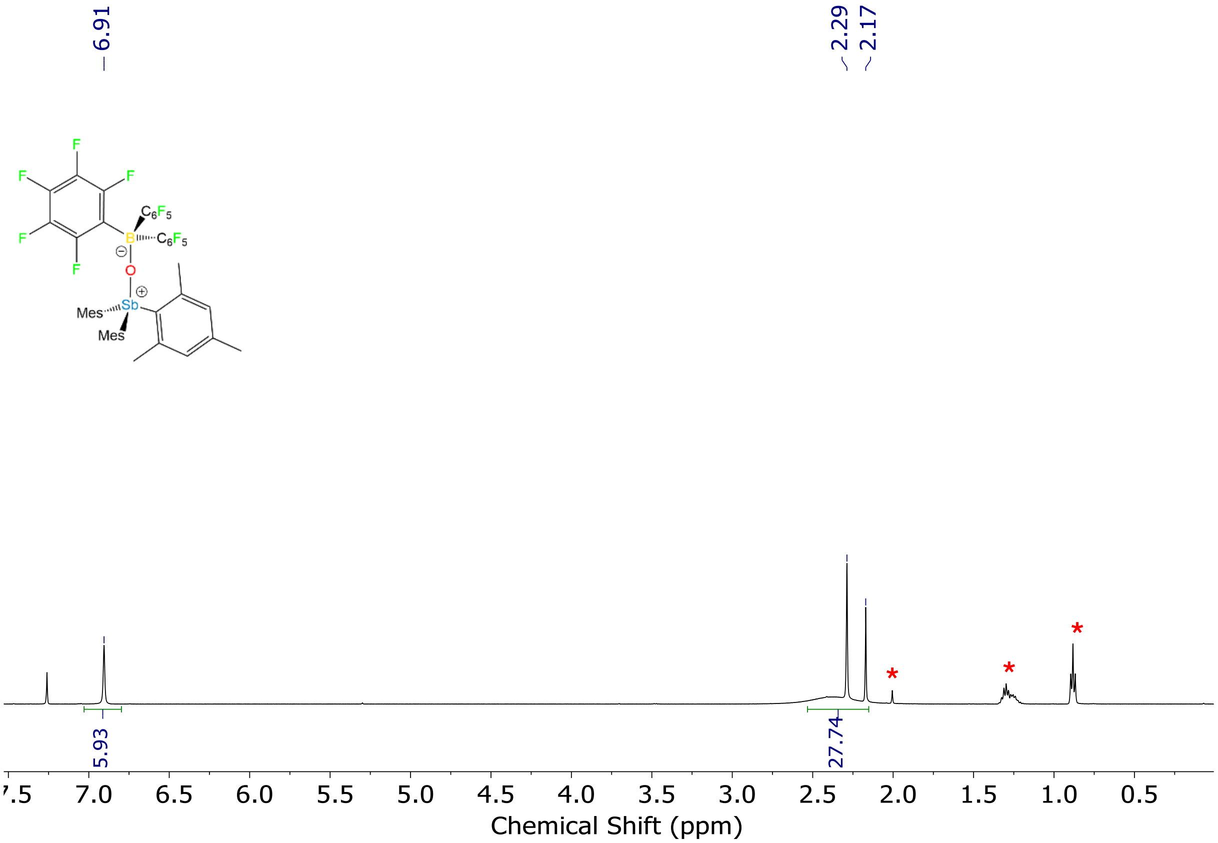

Figure S6. ^{1}H NMR spectrum ($CDCl_3$, 500 MHz) of $Mes_3SbO{\cdot}B(C_6F_5)_3{\cdot}(CHCl_3)$ at room temperature. The asterisks denote signals that correspond to trace pentane and acetonitrile in the sample.

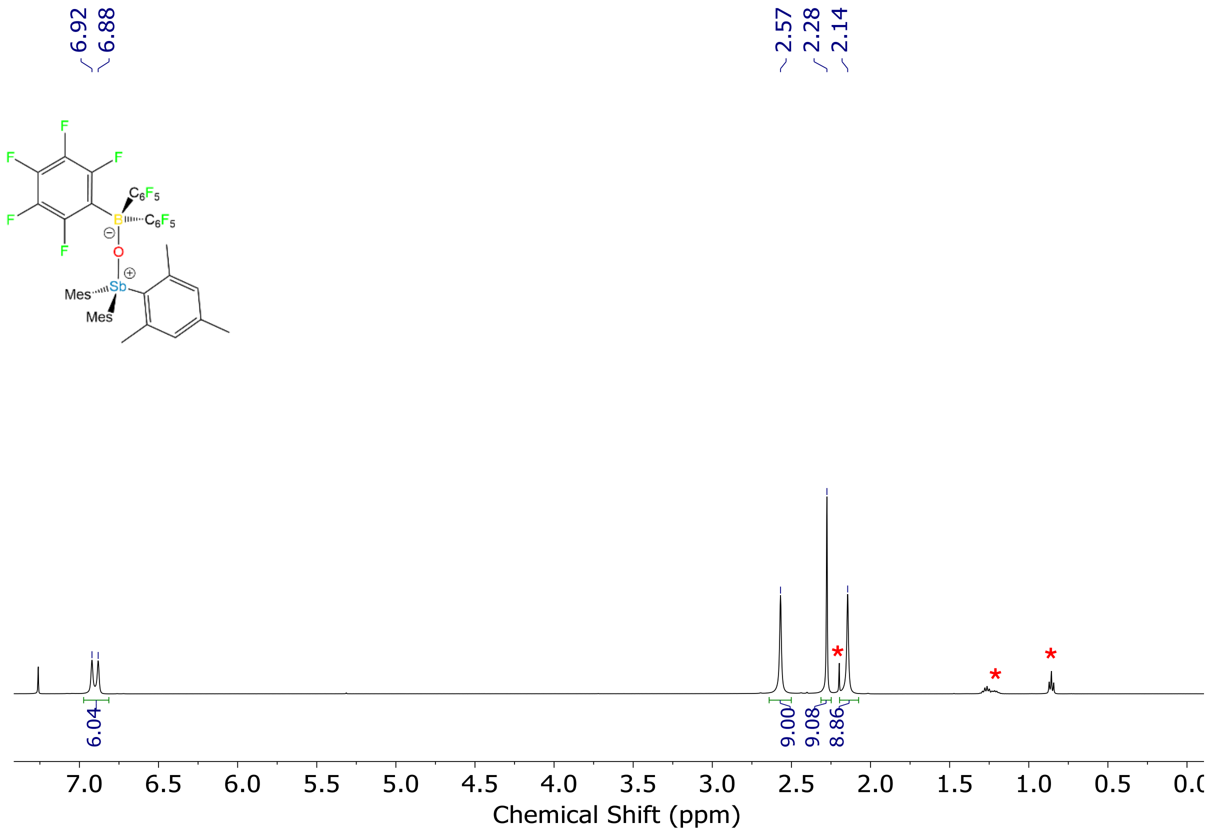

Figure S7. ^{1}H NMR spectrum ($CDCl_3$, 500 MHz) of $Mes_3SbO{\cdot}B(C_6F_5)_3{\cdot}(CHCl_3)$ at –20 °C. The asterisks denote signals that correspond to trace pentane and acetonitrile in the sample.

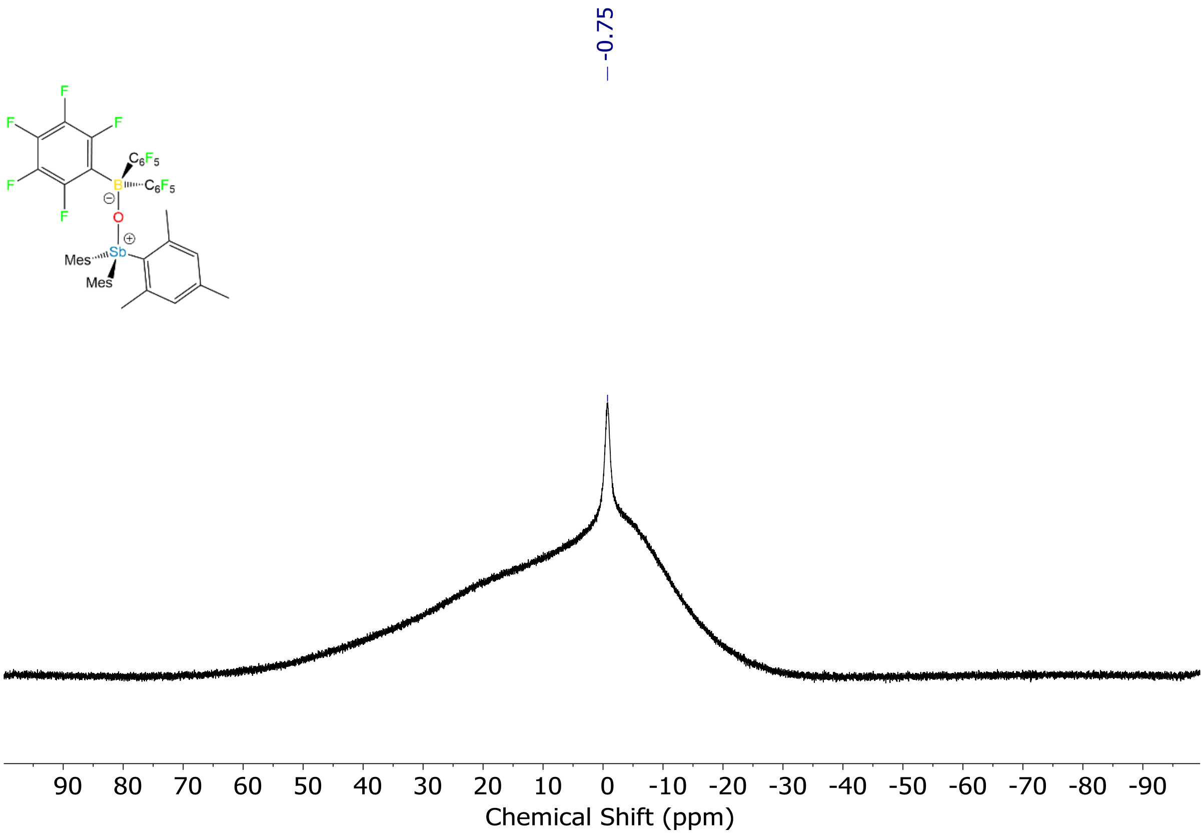

Figure S8. $^{11}B\{^1H\}$ NMR spectrum ($CDCl_3$, 160 MHz) of $Mes_3SbO{\cdot}B(C_6F_5)_3{\cdot}(CHCl_3)$ at room temperature.

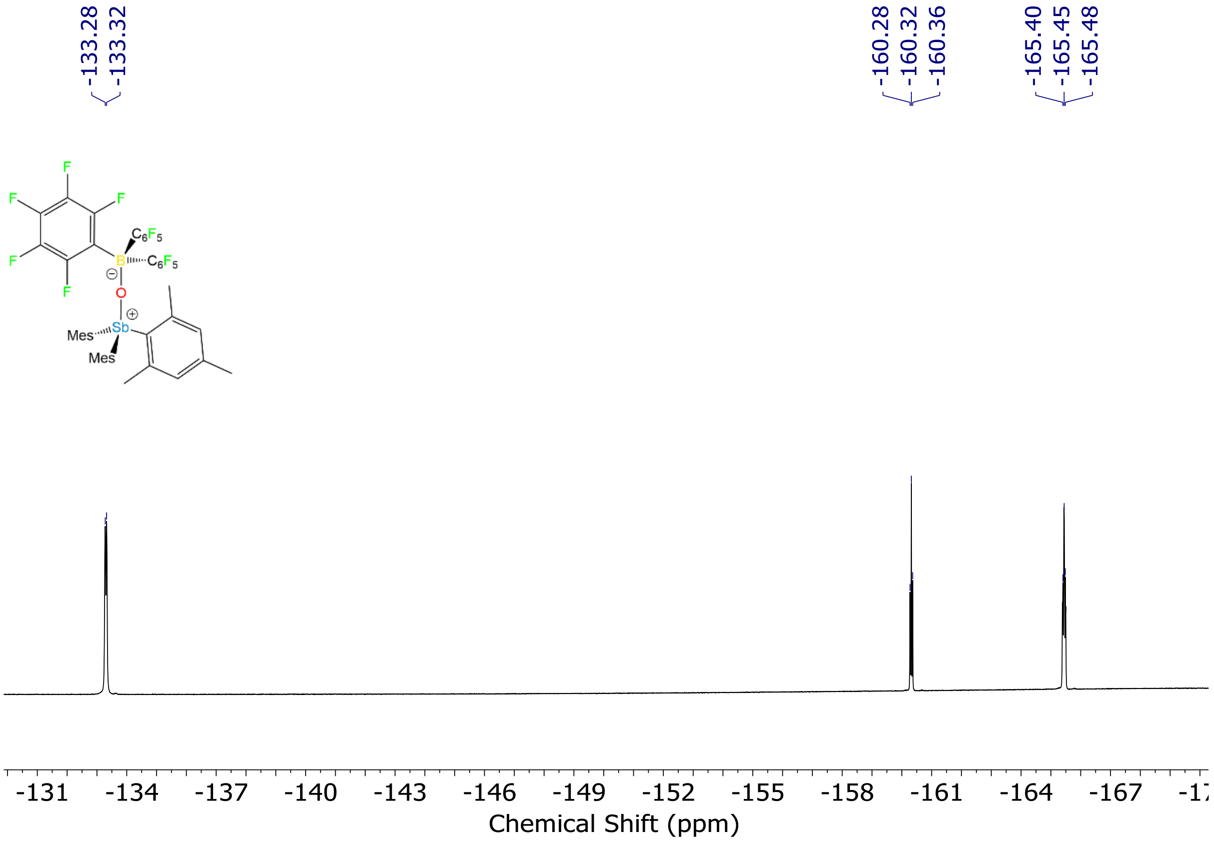

Figure S9. $^{19}F\{^1H\}$ NMR spectrum ($CDCl_3$, 470 MHz) of $Mes_3SbO{\cdot}B(C_6F_5)_3{\cdot}(CHCl_3)$ at room temperature.

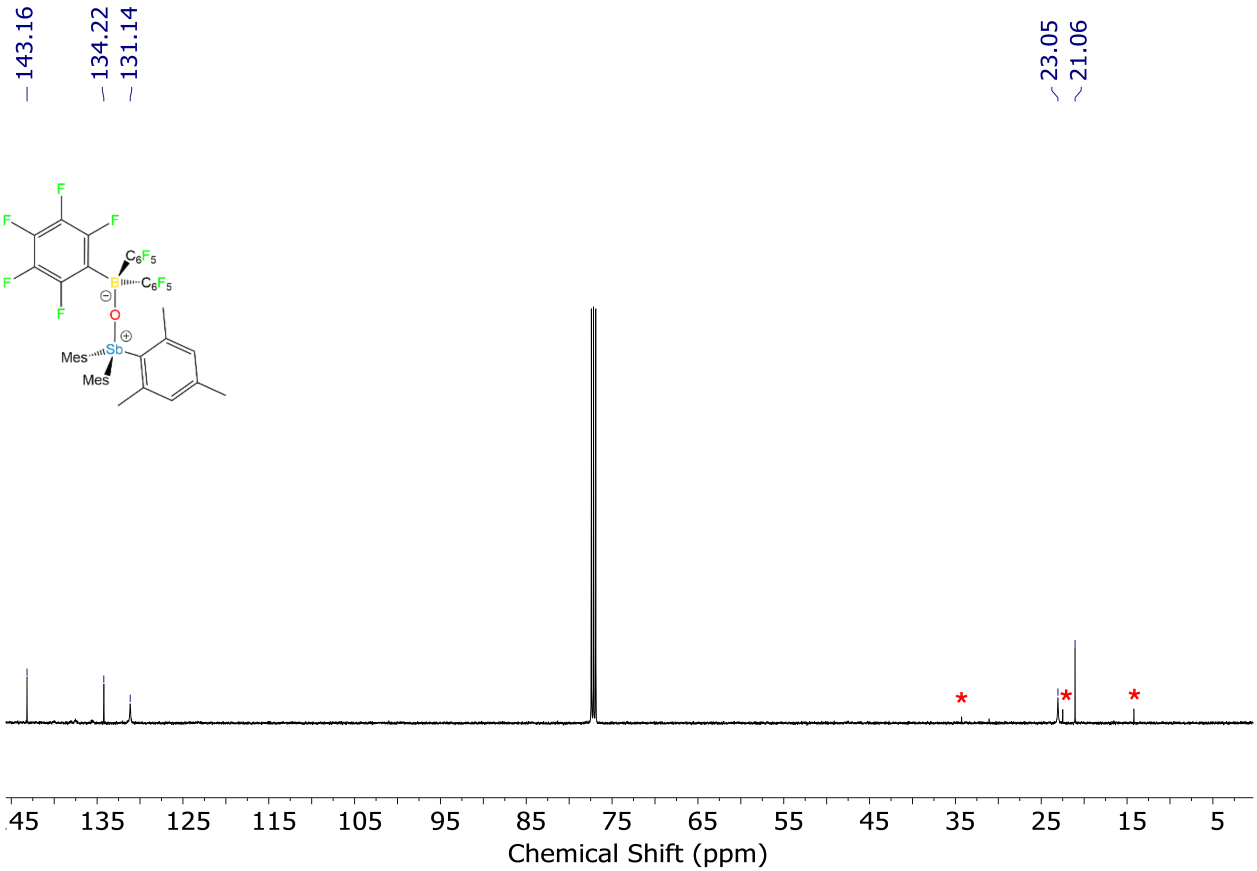

Figure S10. $^{13}C\{^1H\}$ NMR spectrum ($CDCl_3$, 125 MHz) of $Mes_3SbO\cdot B(C_6F_5)_3\cdot(CHCl_3)$ at room temperature. The asterisks denote signals that correspond to trace pentane in the sample.

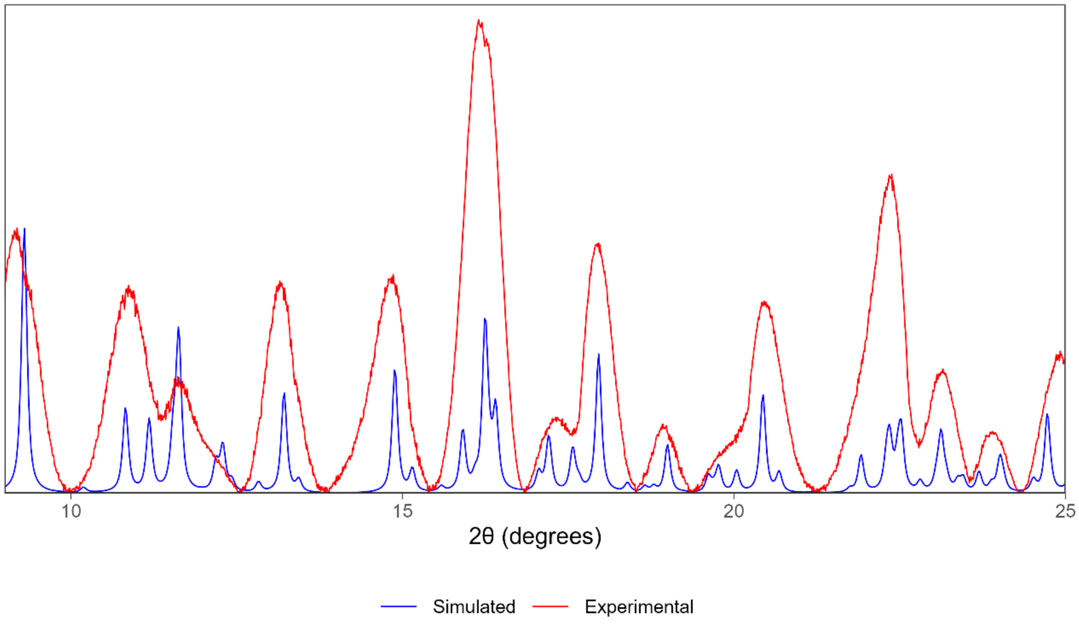

Figure S11. Simulated and experimental powder X-ray diffractograms of $Mes_3SbO\cdot B(C_6F_5)_3\cdot(CHCl_3)$.

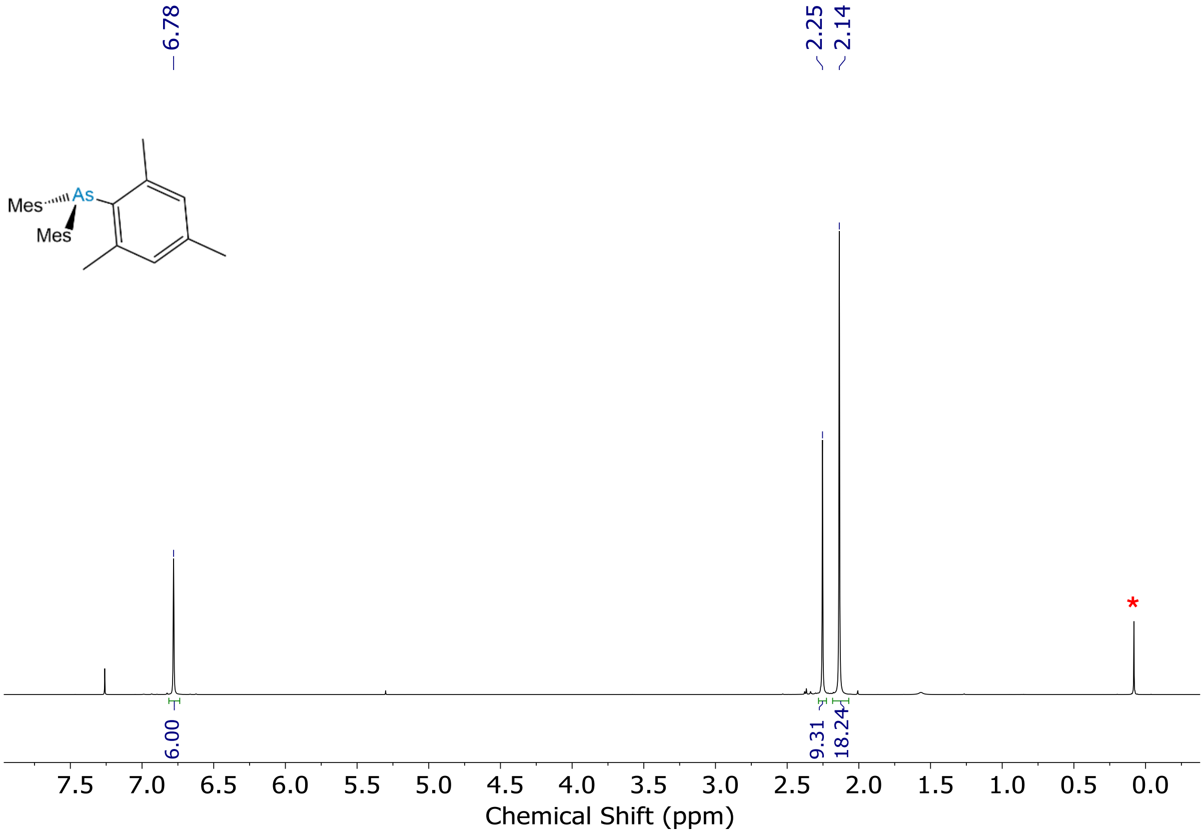

Figure S12. ^{1}H NMR spectrum ($CDCl_3$, 500 MHz) of Mes_3As. The asterisk denotes a signal that corresponds to trace silicone grease in the sample.

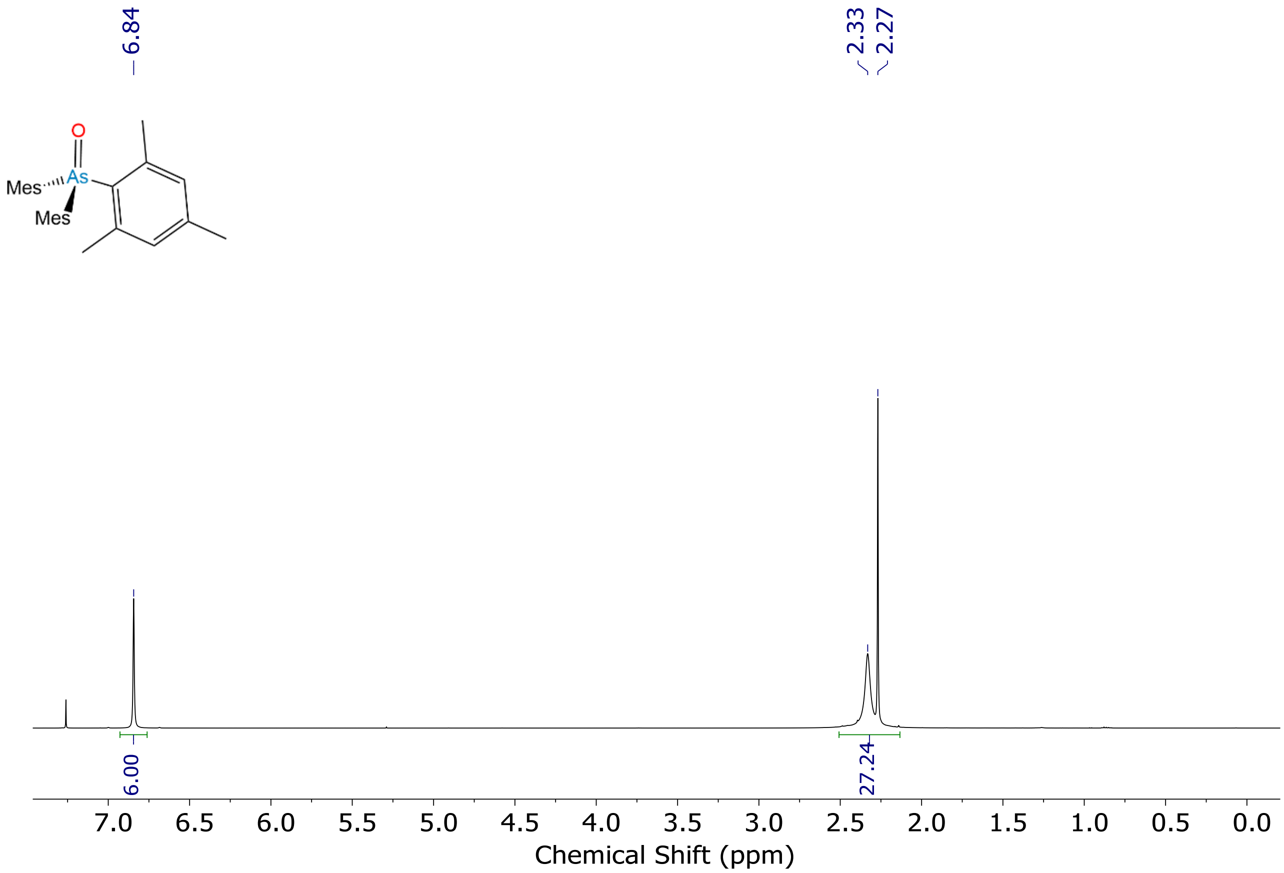

Figure S13. ^{1}H NMR spectrum ($CDCl_3$, 500 MHz) of Mes_3AsO at room temperature.

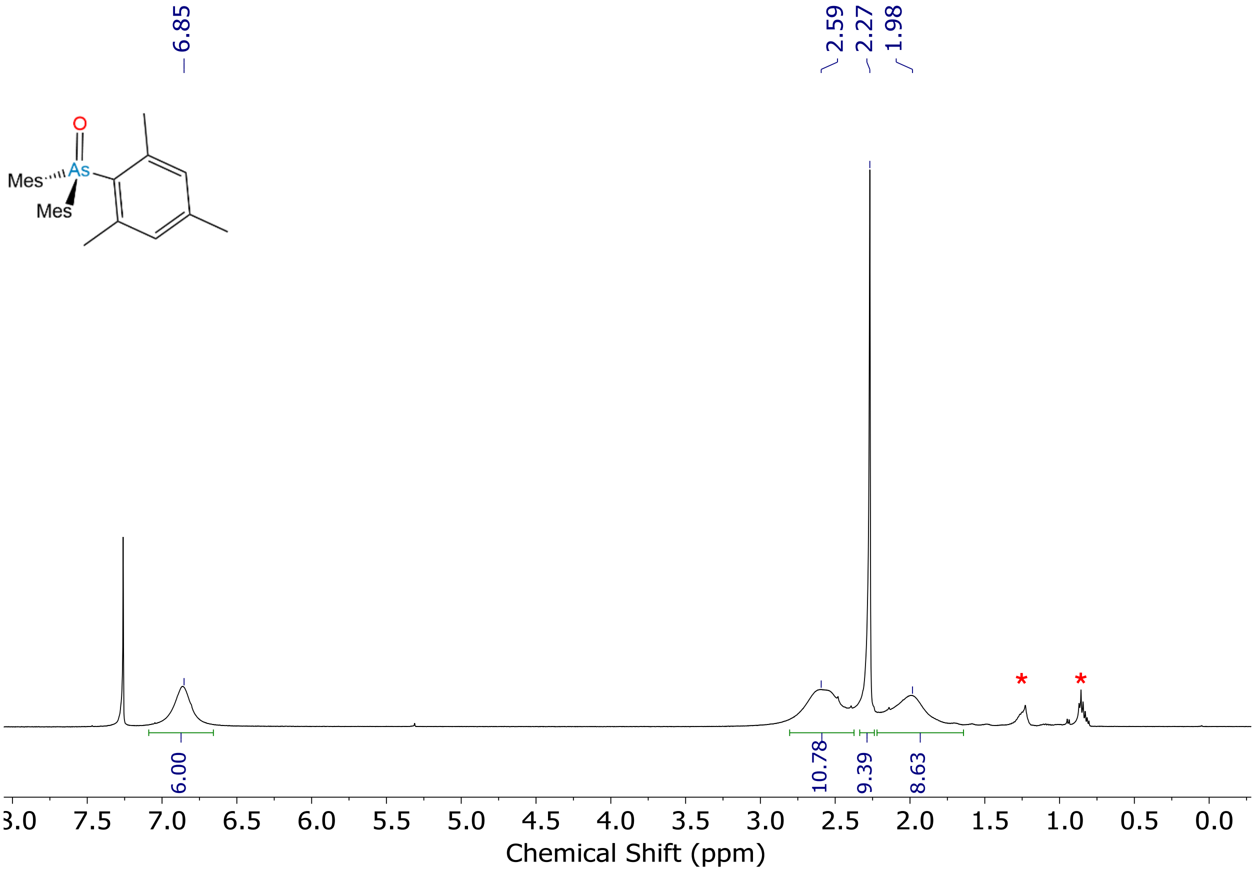

Figure S14. ^{1}H NMR spectrum ($CDCl_3$, 500 MHz) of Mes_3AsO at –20 °C. The asterisks denote signals that correspond to pentane.

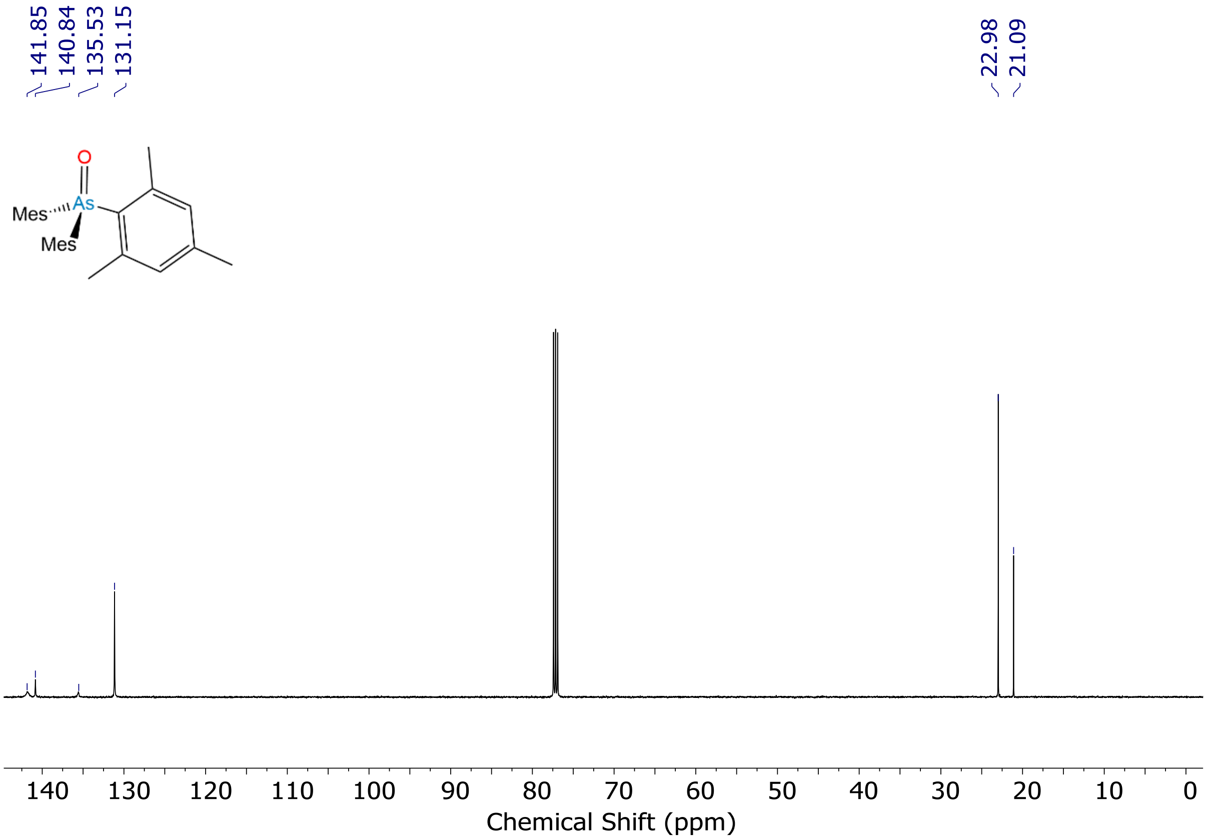

Figure S15. $^{13}C\{^{1}H\}$ NMR spectrum ($CDCl_3$, 125 MHz) of Mes_3AsO at room temperature.

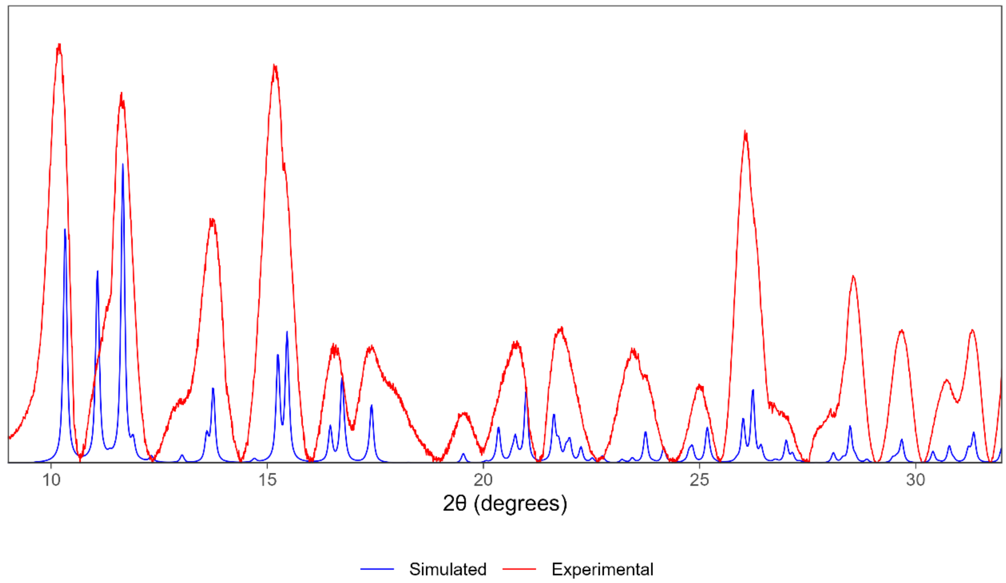

Figure S16. Simulated and experimental powder X-ray diffractograms of Mes_3AsO.

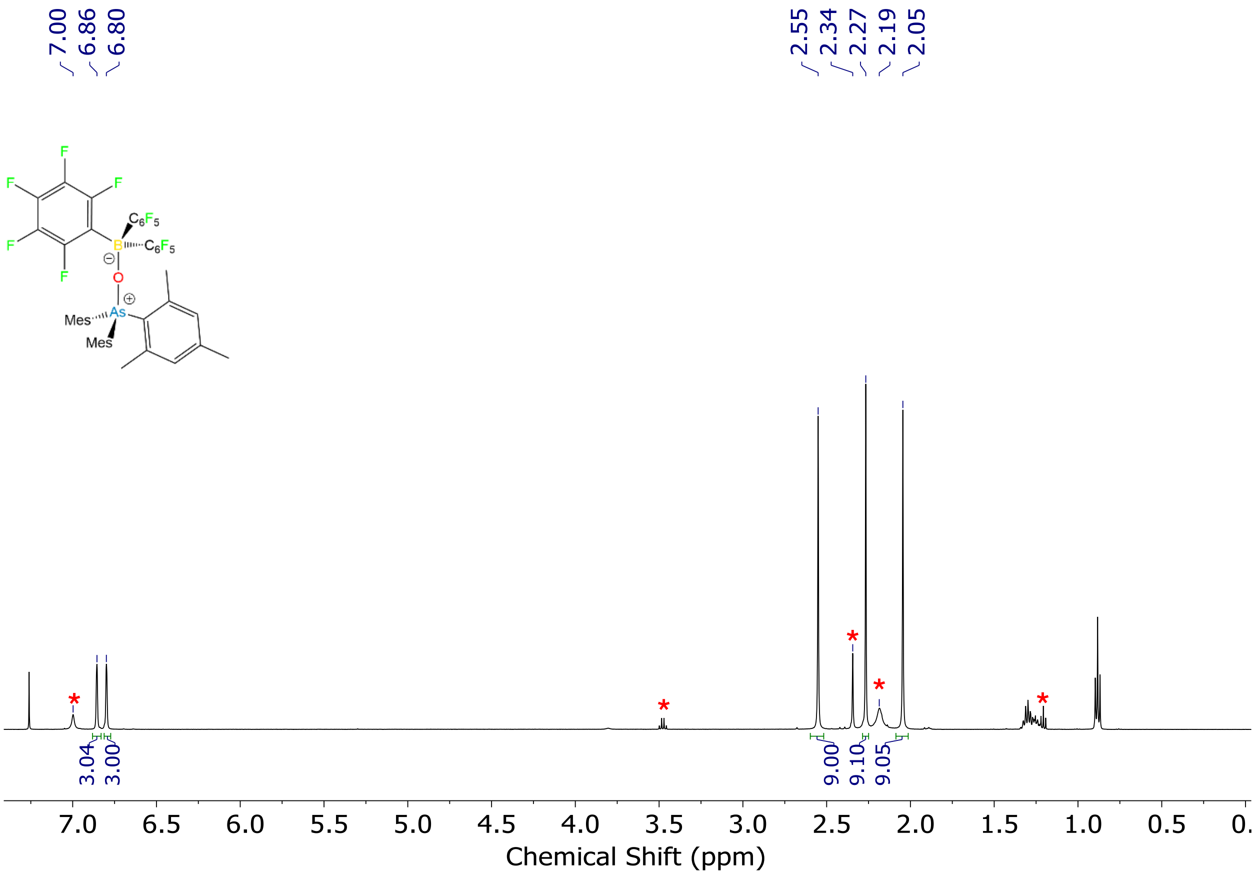

Figure S17. ^1H NMR spectrum ($CDCl_3$, 500 MHz) of $Mes_3AsO{\cdot}B(C_6F_5)_3$. The asterisks denote signals that correspond to $[Mes_3AsOH][B(C_6F_5)_3(OH)]$ and $Et_2O{\cdot}B(C_6F_5)_3$.

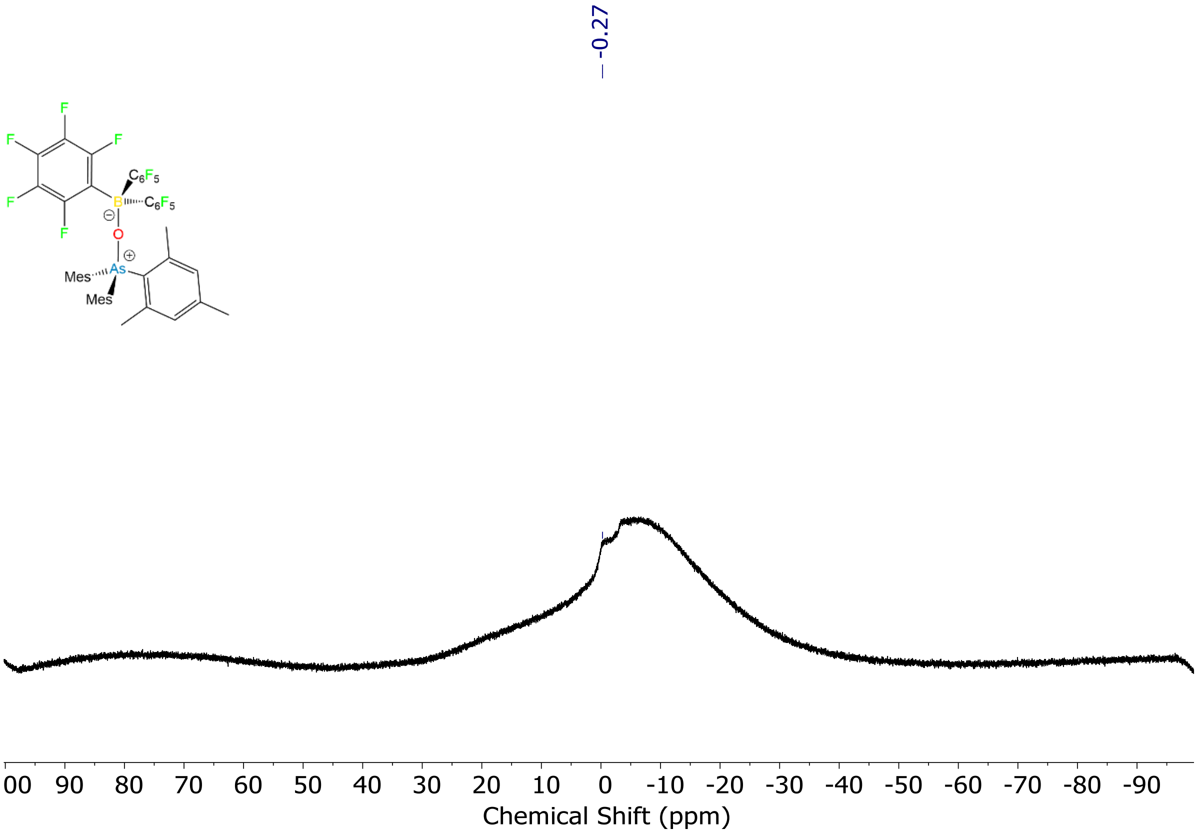

Figure S18. $^{11}B\{^{1}H\}$ NMR spectrum ($CDCl_3$, 160 MHz) of $Mes_3AsO{\cdot}B(C_6F_5)_3$.

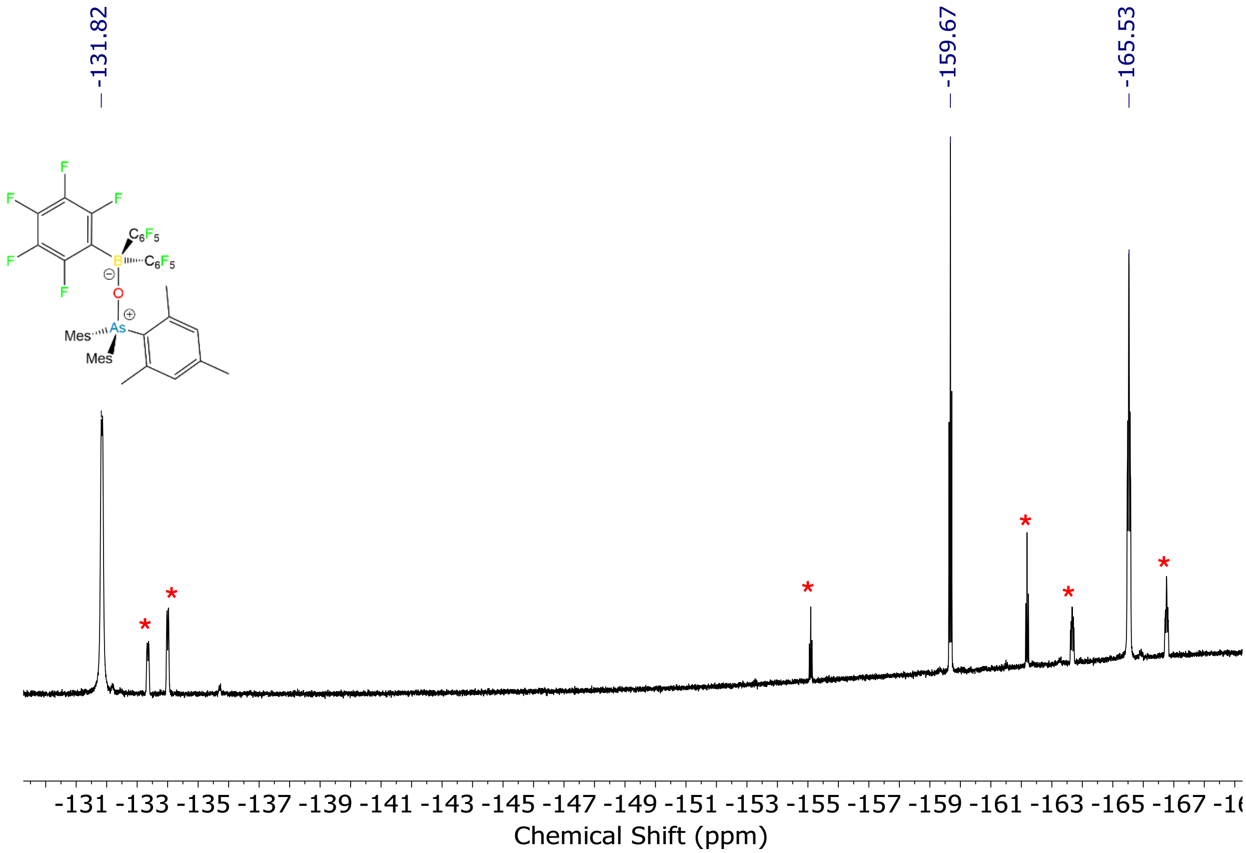

Figure S19. $^{19}F\{^{1}H\}$ NMR spectrum ($CDCl_3$, 470 MHz) of $Mes_3AsO{\cdot}B(C_6F_5)_3$. The asterisks denote signals that correspond to $[Mes_3AsOH][B(C_6F_5)_3(OH)]$ and $Et_2O{\cdot}B(C_6F_5)_3$.

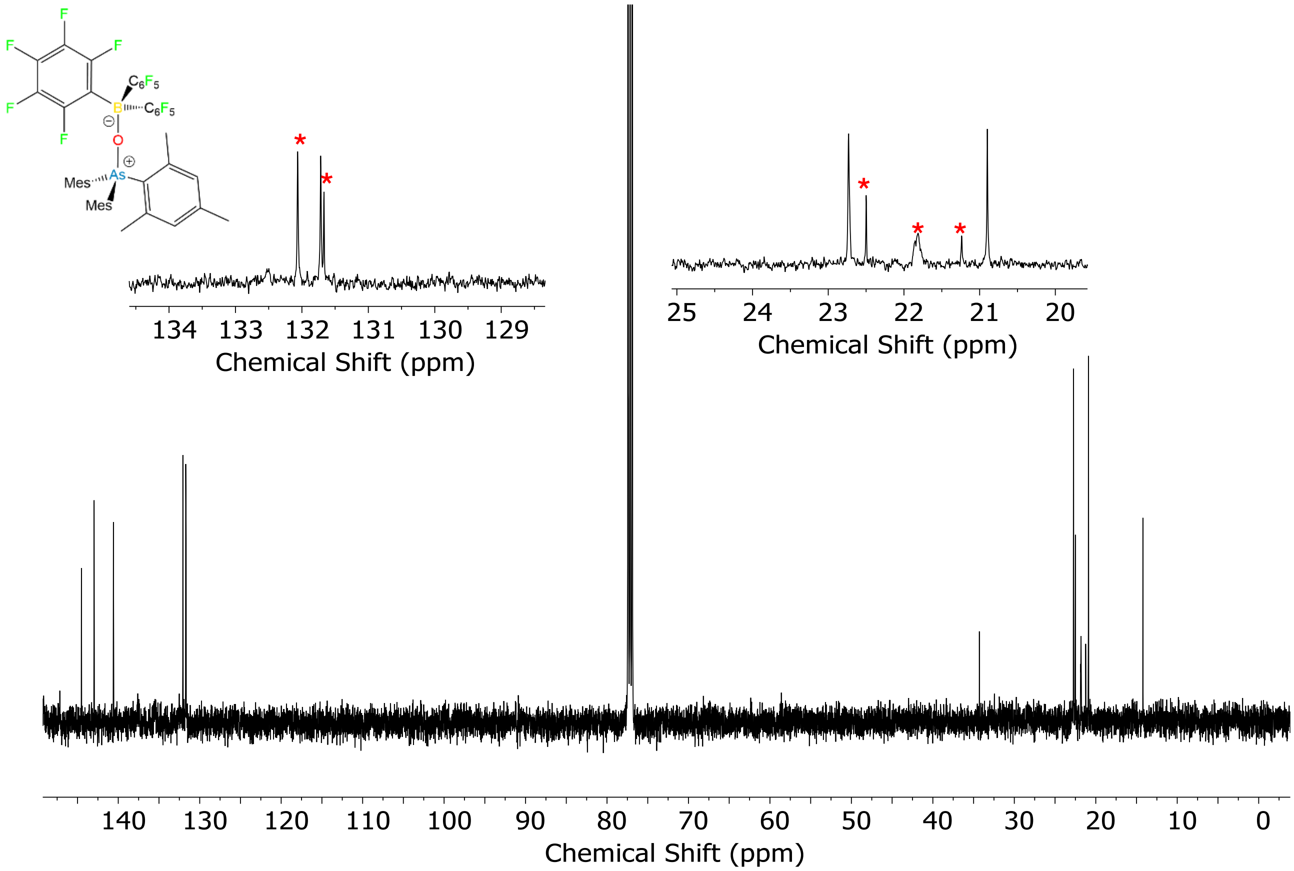

Figure S20. $^{13}C\{^{1}H\}$ NMR spectrum ($CDCl_3$, 125 MHz) of $Mes_3AsO{\cdot}B(C_6F_5)_3$. The asterisks denote signals that correspond to pentane, $[Mes_3AsOH][B(C_6F_5)_3(OH)]$ and $Et_2O{\cdot}B(C_6F_5)_3$.

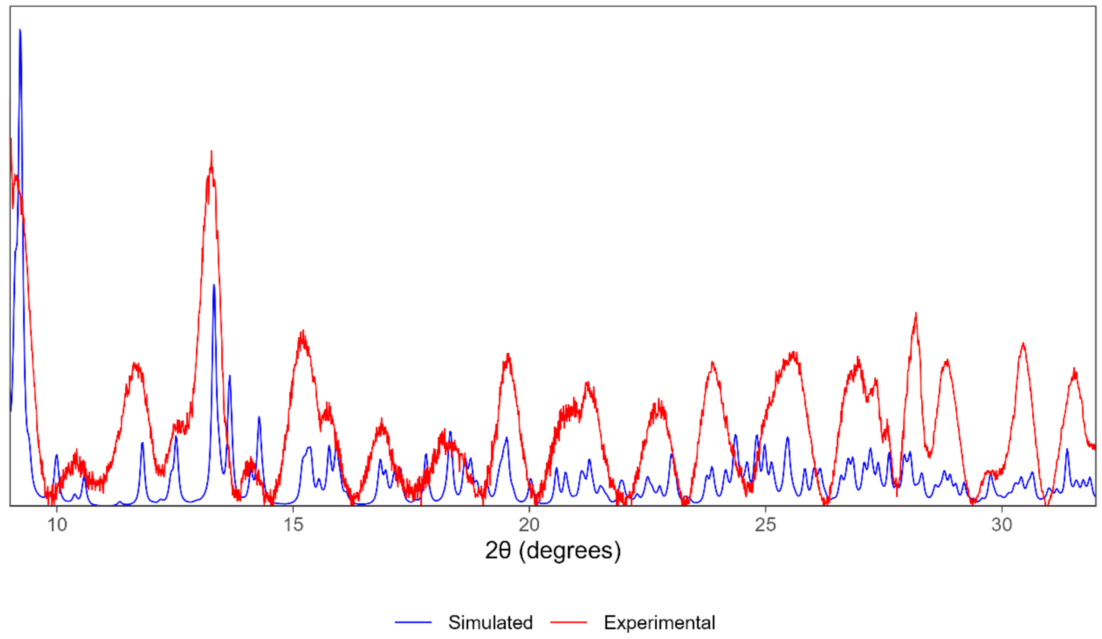

Figure S21. Simulated and experimental powder X-ray diffractograms of $Mes_3AsO{\cdot}B(C_6F_5){\cdot}(CH_2Cl_2)$.

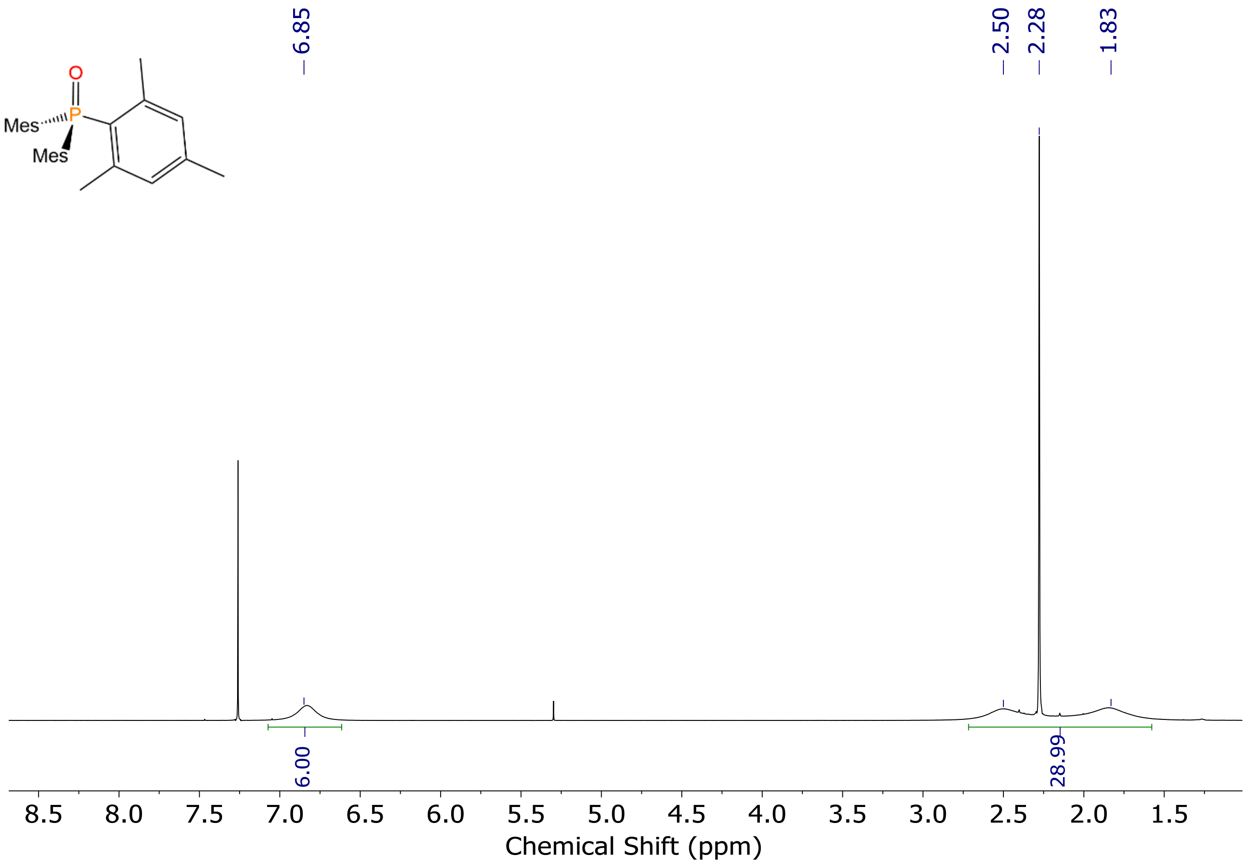

Figure S22. ^{1}H NMR spectrum ($CDCl_3$, 500 MHz) of Mes_3PO.

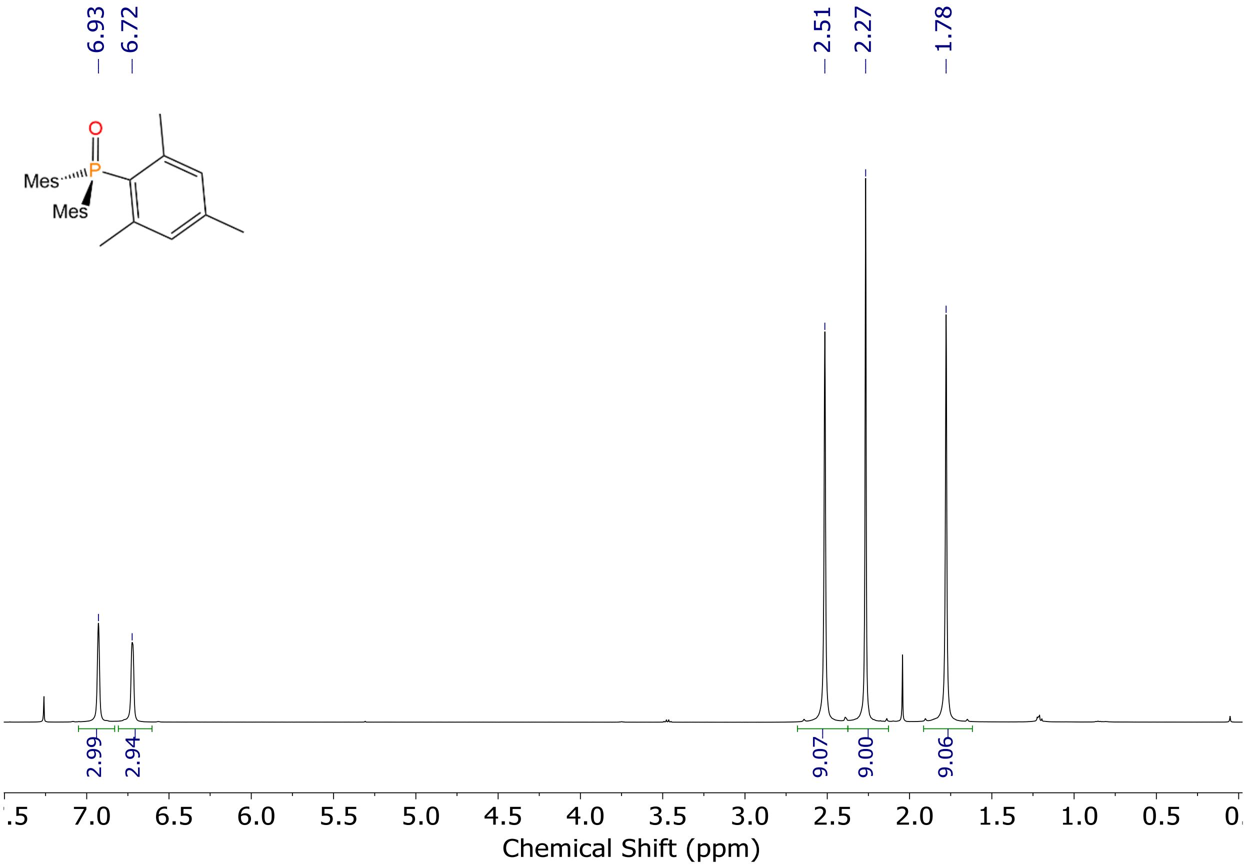

Figure S23. ^{1}H NMR spectrum ($CDCl_3$, 500 MHz) of Mes_3PO at –20 °C.

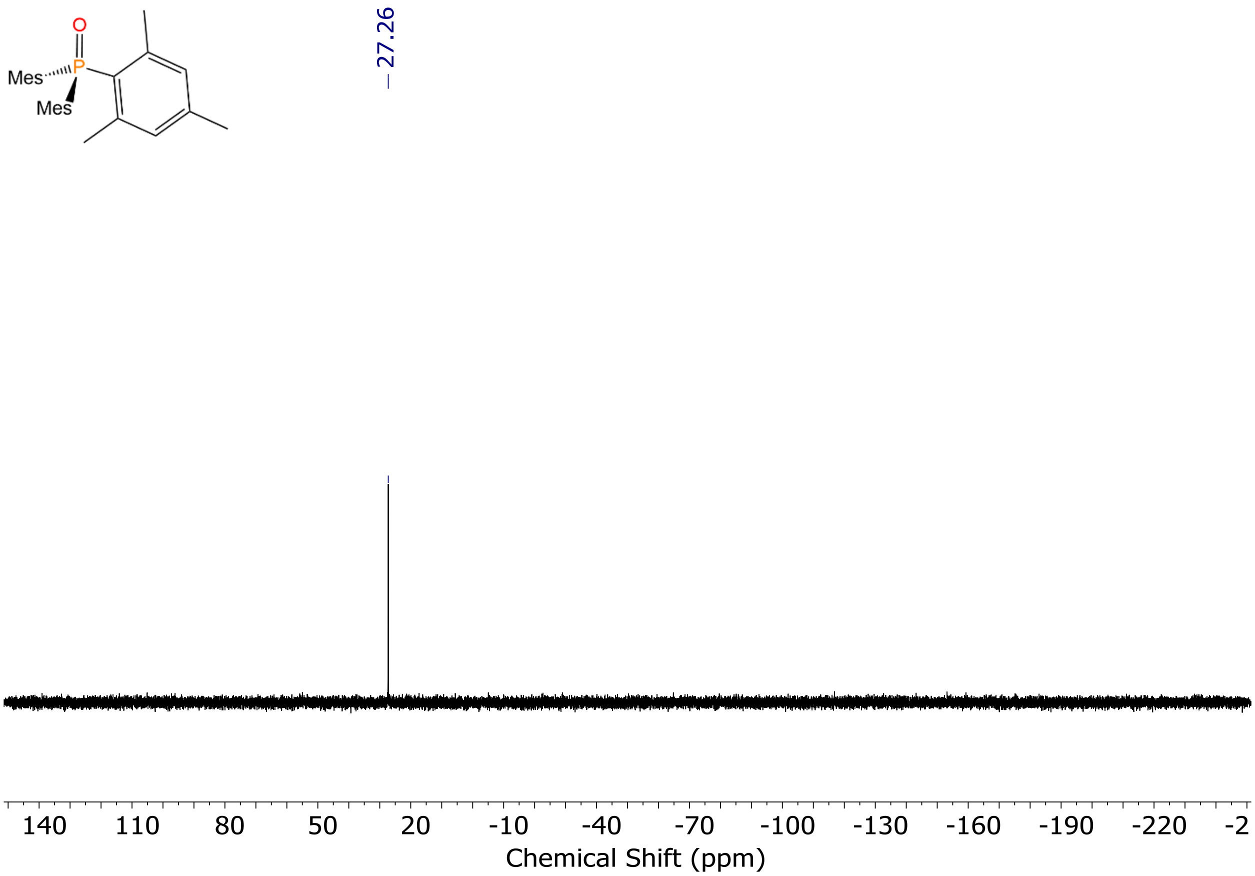

Figure S24. $^{31}P\{^1H\}$ NMR spectrum ($CDCl_3$, 202 MHz) of Mes_3PO.

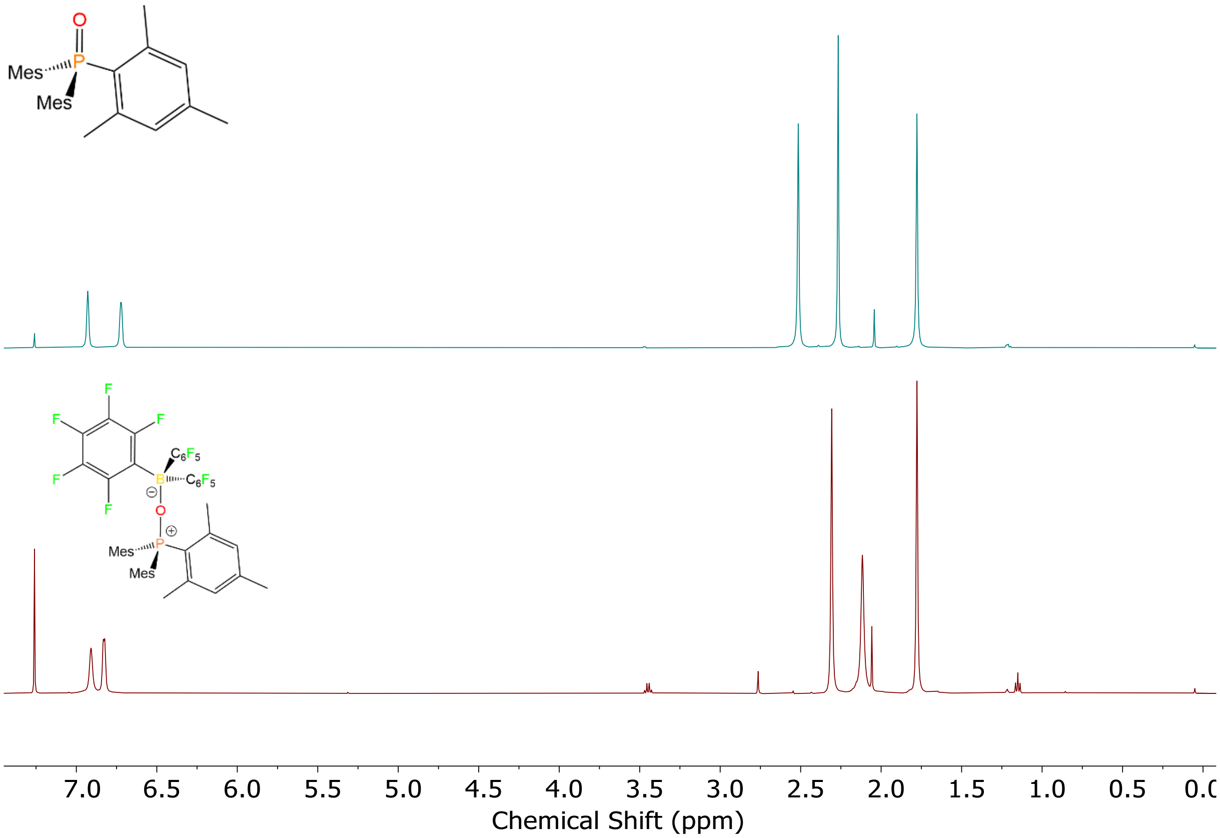

Figure S25. ^1H NMR spectrum ($CDCl_3$, 500 MHz) of Mes_3PO and $Mes_3PO{\cdot}B(C_6F_5)_3$ at –20 °C.

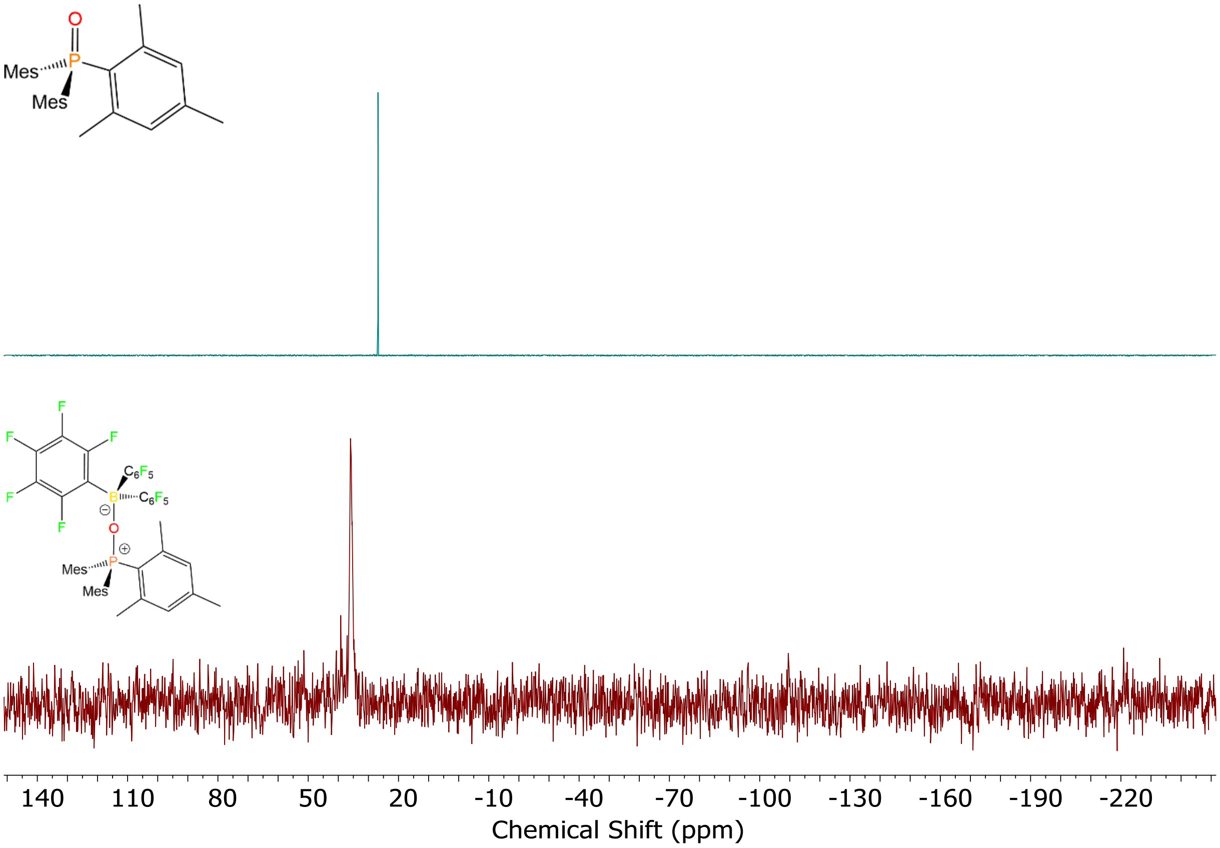

Figure S26. ³¹P{¹H} NMR spectrum ($CDCl_3$, 202 MHz) of Mes_3PO and $Mes_3PO{\cdot}B(C_6F_5)_3$ at –20 °C.

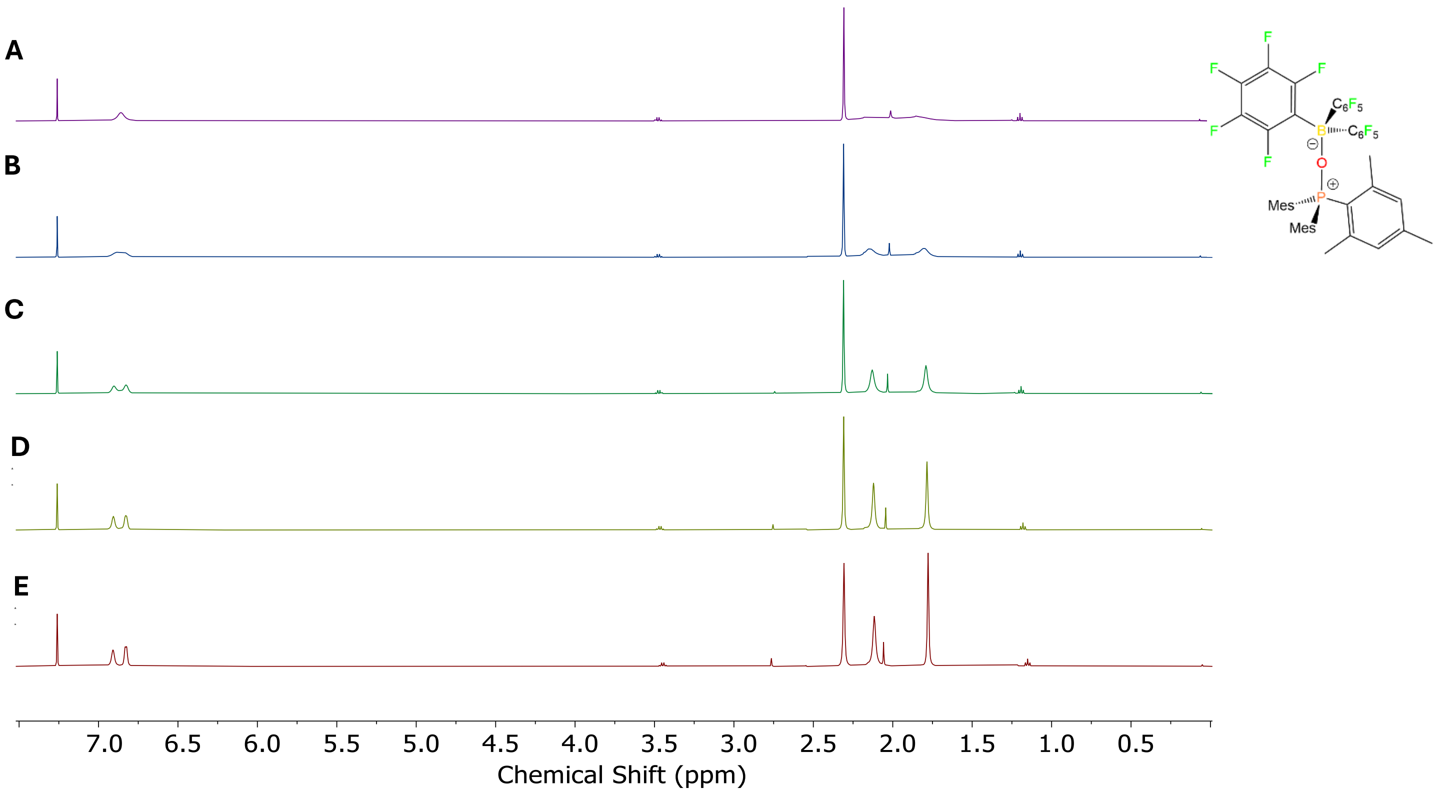

Figure S27. *In situ* ¹H NMR spectrum ($CDCl_3$, 500 MHz) of a 1:1 mixture of Mes_3PO and $B(C_6F_5)_3$ at varying temperatures. (A) 20 °C, (B) 10 °C, (C) 0 °C, (D) –10 °C, (E) –20 °C.

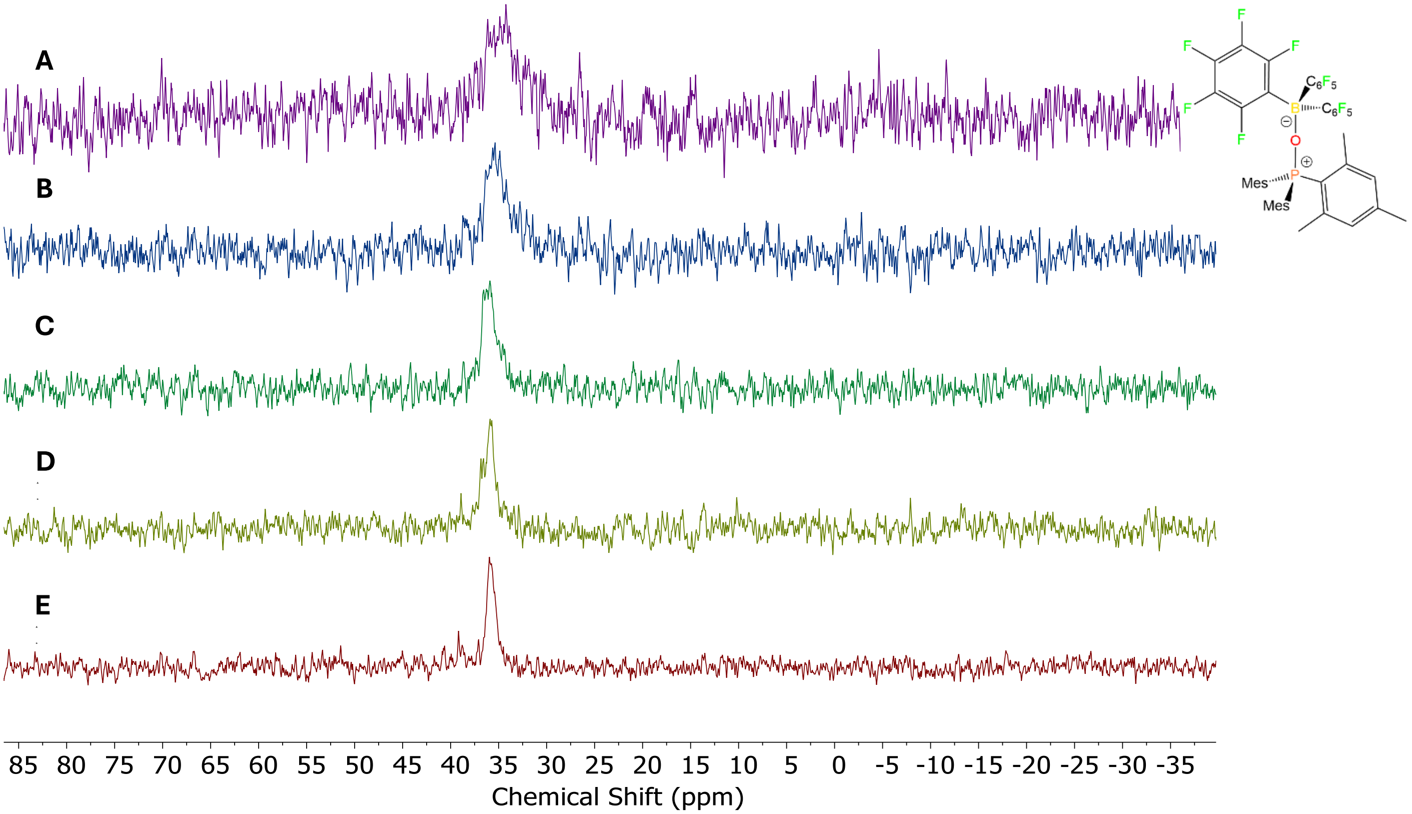

Figure S28. *In situ* $^{31}P\{^{1}H\}$ NMR spectrum ($CDCl_3$, 202 MHz) of a 1:1 mixture of Mes_3PO and $B(C_6F_5)_3$ at varying temperatures. (A) 20 °C, (B) 10 °C, (C) 0 °C, (D) –10 °C, (E) –20 °C.

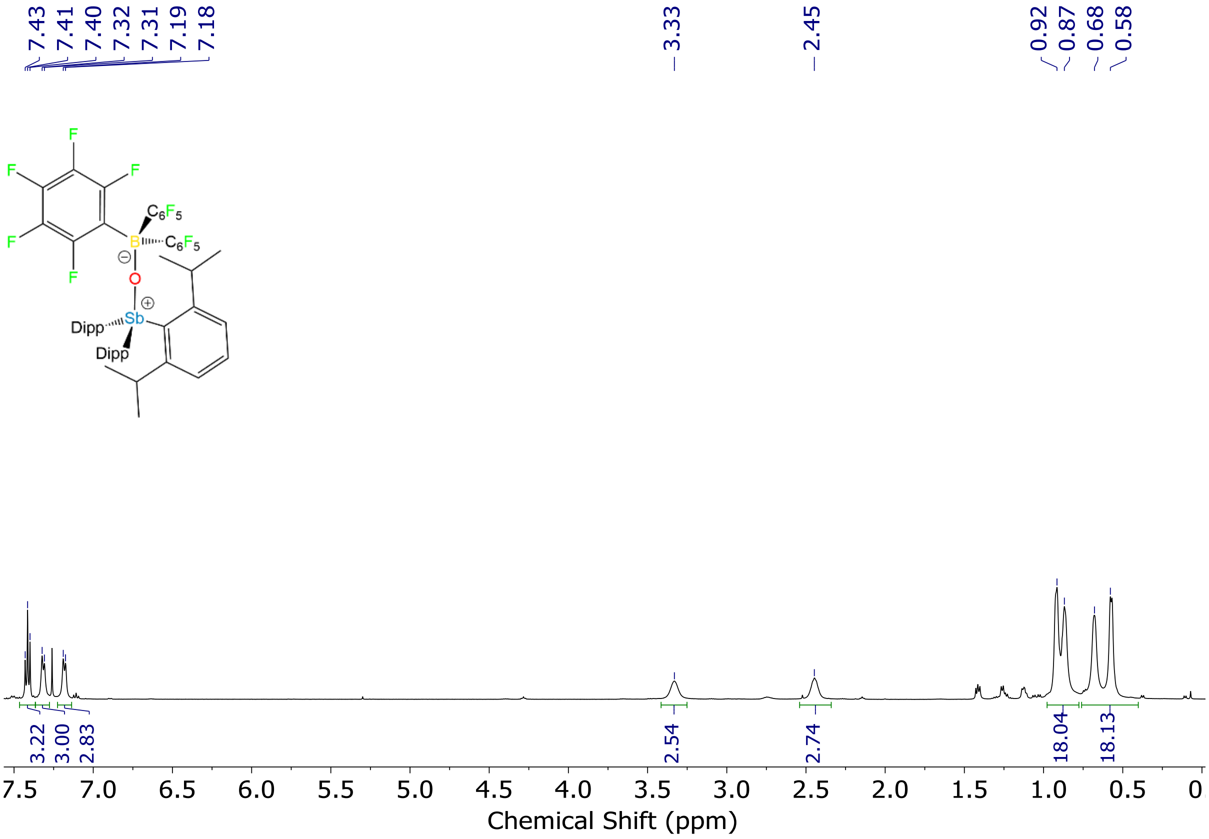

Figure S29. *In situ* ^{1}H NMR spectrum ($CDCl_3$, 500 MHz) of $Dipp_3SbO\cdot B(C_6F_5)_3$.

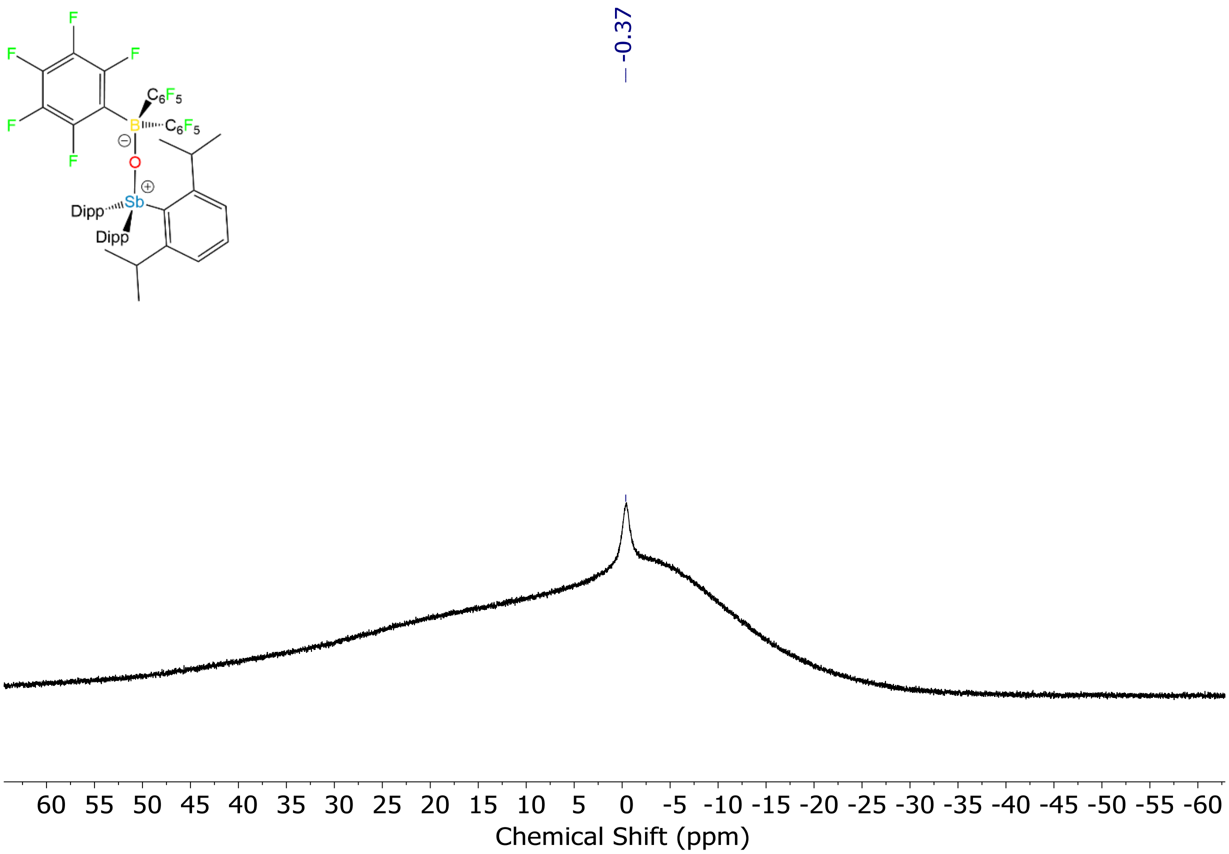

Figure S30. *In situ* $^{11}B\{^1H\}$ NMR spectrum ($CDCl_3$, 160 MHz) of $Dipp_3SbO{\cdot}B(C_6F_5)_3$.

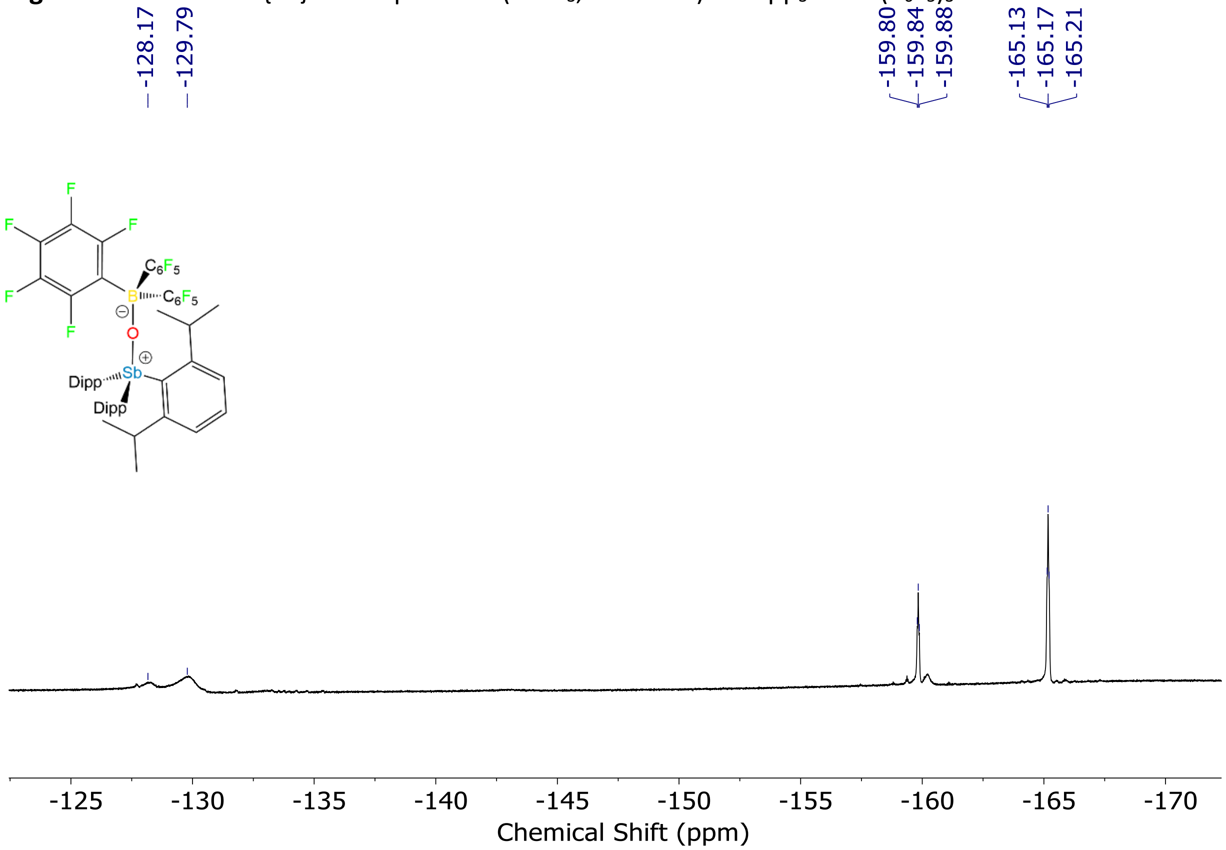

Figure S31. *In situ* $^{19}F\{^1H\}$ NMR spectrum ($CDCl_3$, 470 MHz) of $Dipp_3SbO{\cdot}B(C_6F_5)_3$.

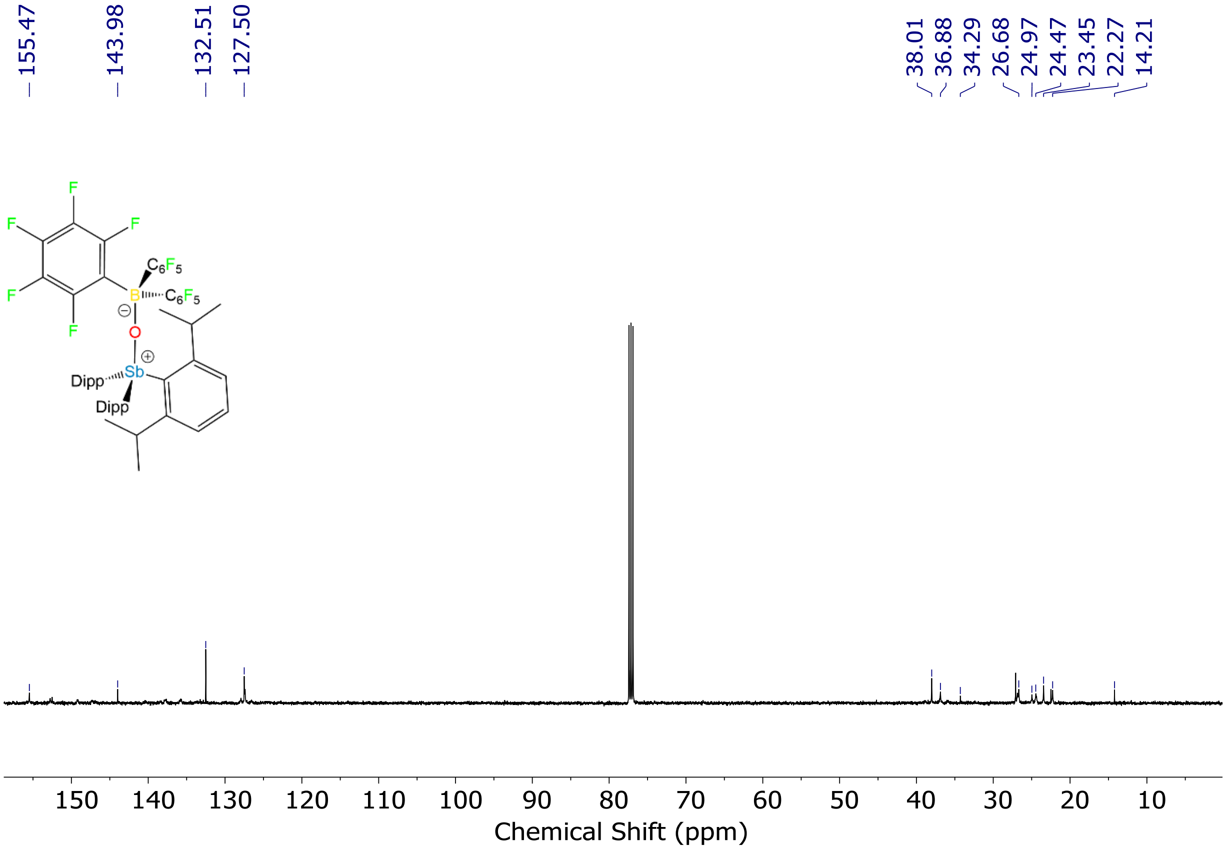

Figure S32. *In situ* $^{13}C\{^1H\}$ NMR spectrum ($CDCl_3$, 125 MHz) of $Dipp_3SbO{\cdot}B(C_6F_5)_3$.

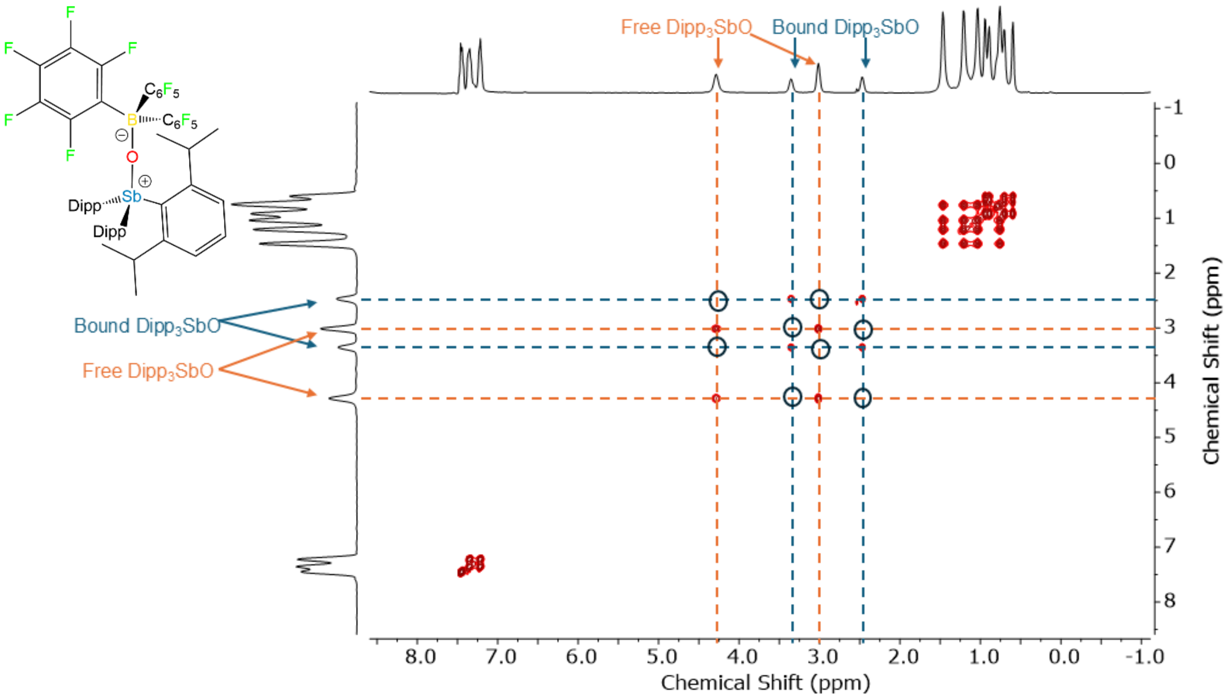

Figure S33. ^1H-^1H EXSY NMR spectrum ($CDCl_3$, 125 MHz, 200 ms mixing time) of free $Dipp_3SbO$ and $Dipp_3SbO{\cdot}B(C_6F_5)_3$. Circles highlight lack of exchange cross-peaks between free and bound $Dipp_3SbO$.

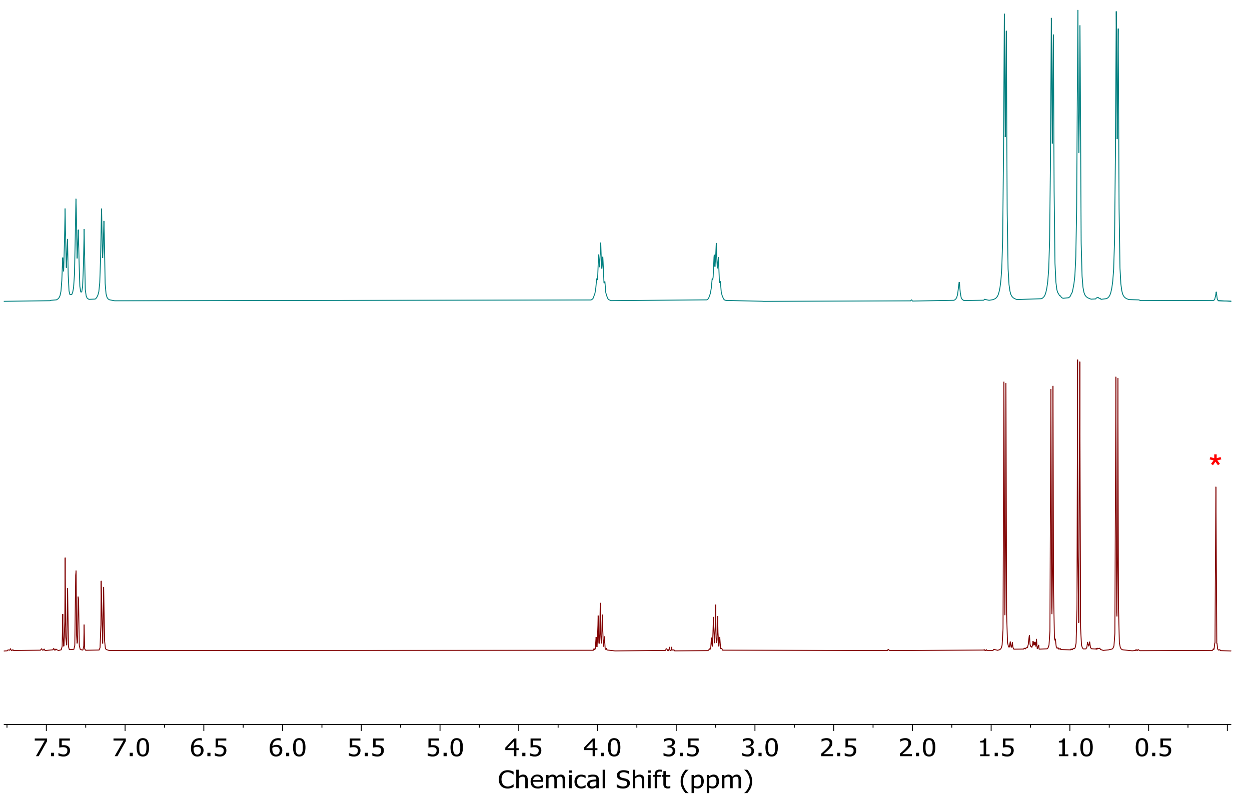

Figure S34. *In situ* ^{1}H NMR spectrum ($CDCl_3$, 500 MHz) of $Dipp_3AsO$ and a 1:1 mixture of $Dipp_3AsO$ and $B(C_6F_5)_3$. The asterisk denotes trace silicone grease in the sample.

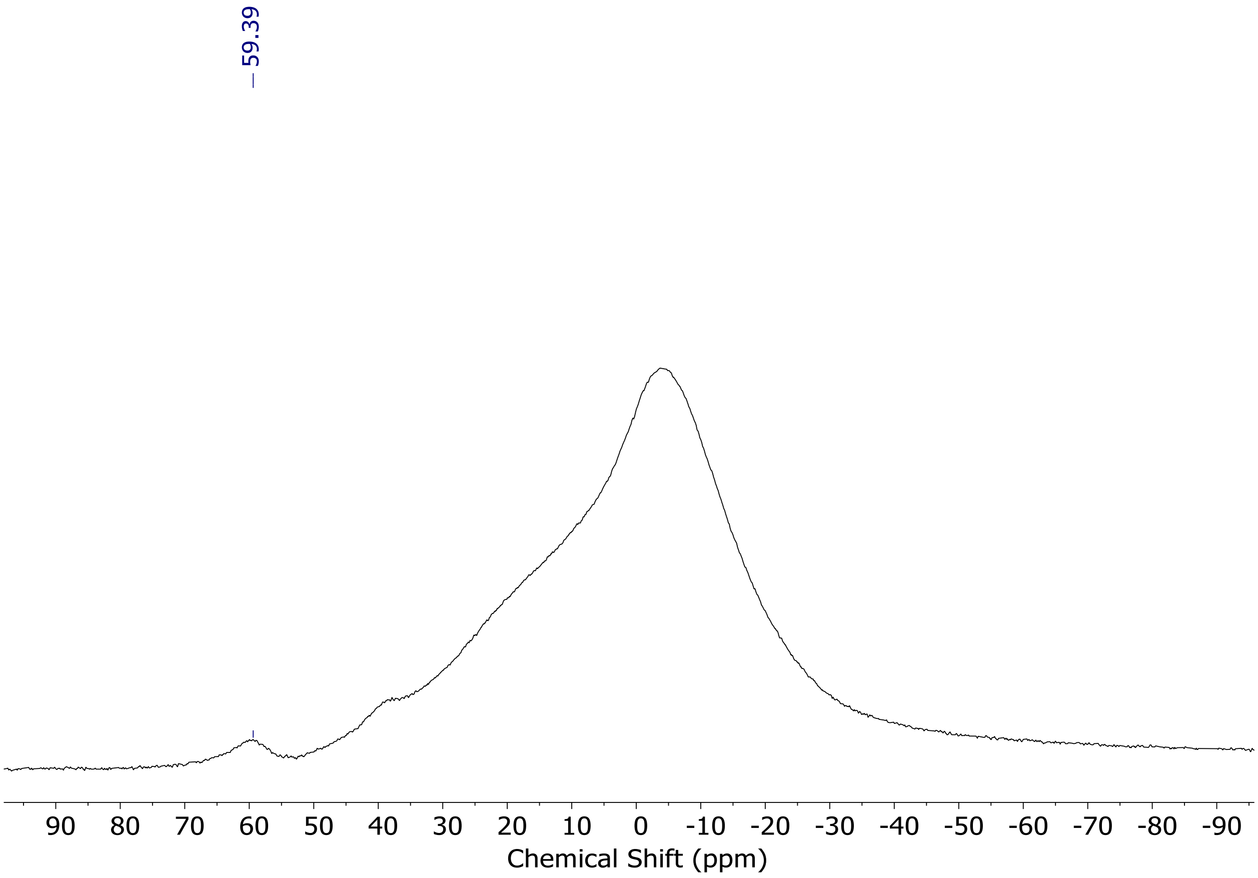

Figure S35. *In situ* $^{11}B\{^{1}H\}$ NMR spectrum ($CDCl_3$, 160 MHz) of a 1:1 mixture of $Dipp_3AsO$ and $B(C_6F_5)_3$.

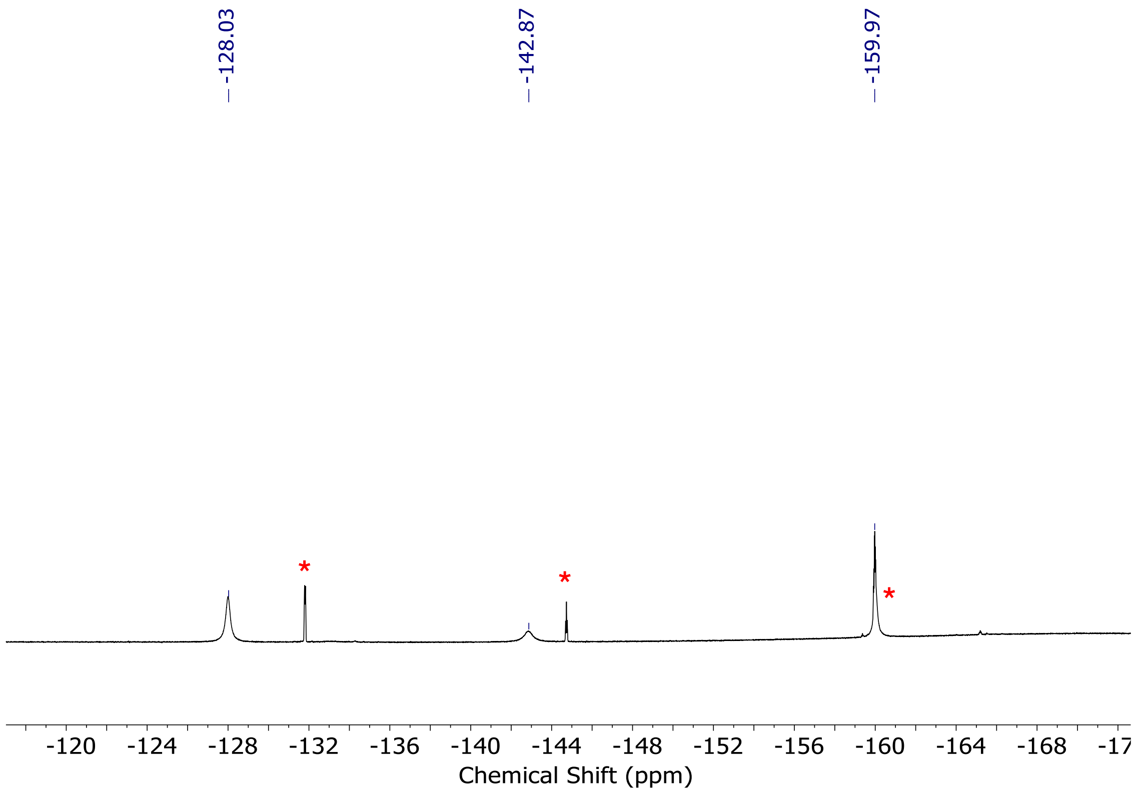

Figure S36. *In situ* $^{19}F\{^{1}H\}$ NMR spectrum ($CDCl_3$, 470 MHz) of a 1:1 mixture of $Dipp_3AsO$ and $B(C_6F_5)_3$. The asterisks denote signals that correspond to $Et_2O{\cdot}B(C_6F_5)_3$.

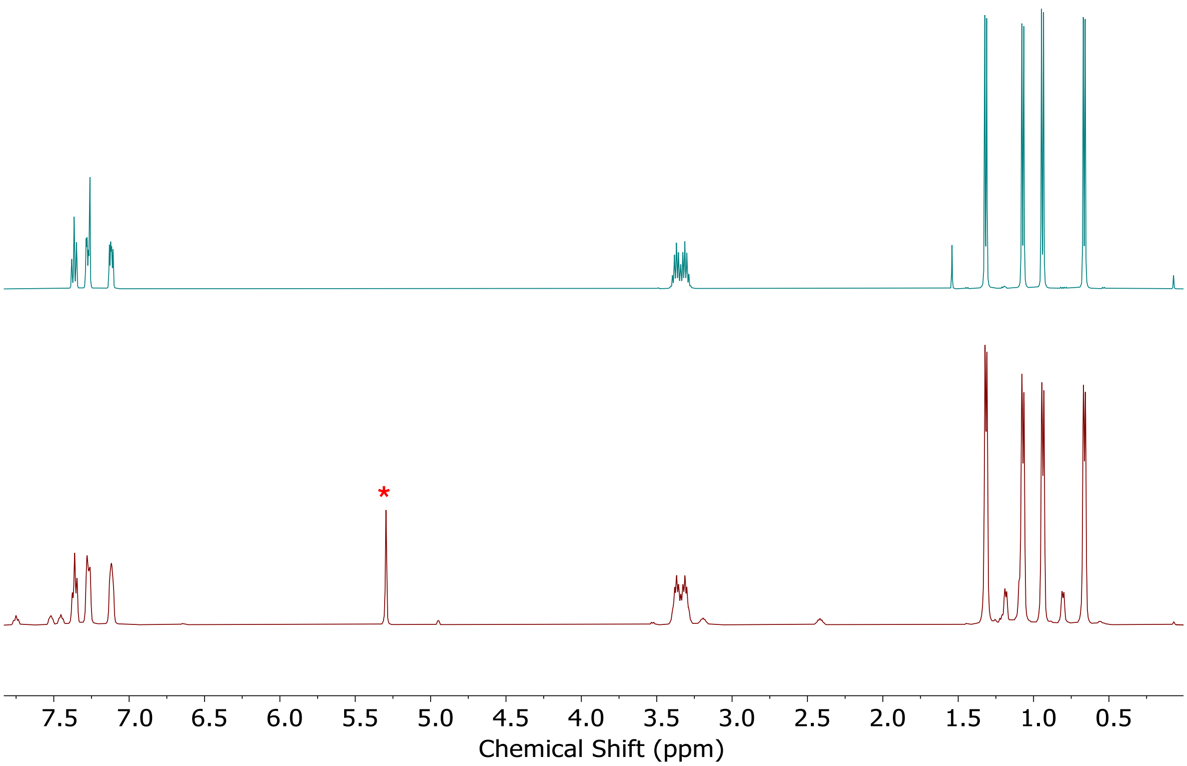

Figure S37. *In situ* ^{1}H NMR spectrum ($CDCl_3$, 500 MHz) of $Dipp_3PO$ (*top*) and a 1:1 mixture of $Dipp_3PO$ and $B(C_6F_5)_3$ (*bottom*). The asterisk denotes trace DCM in the sample.

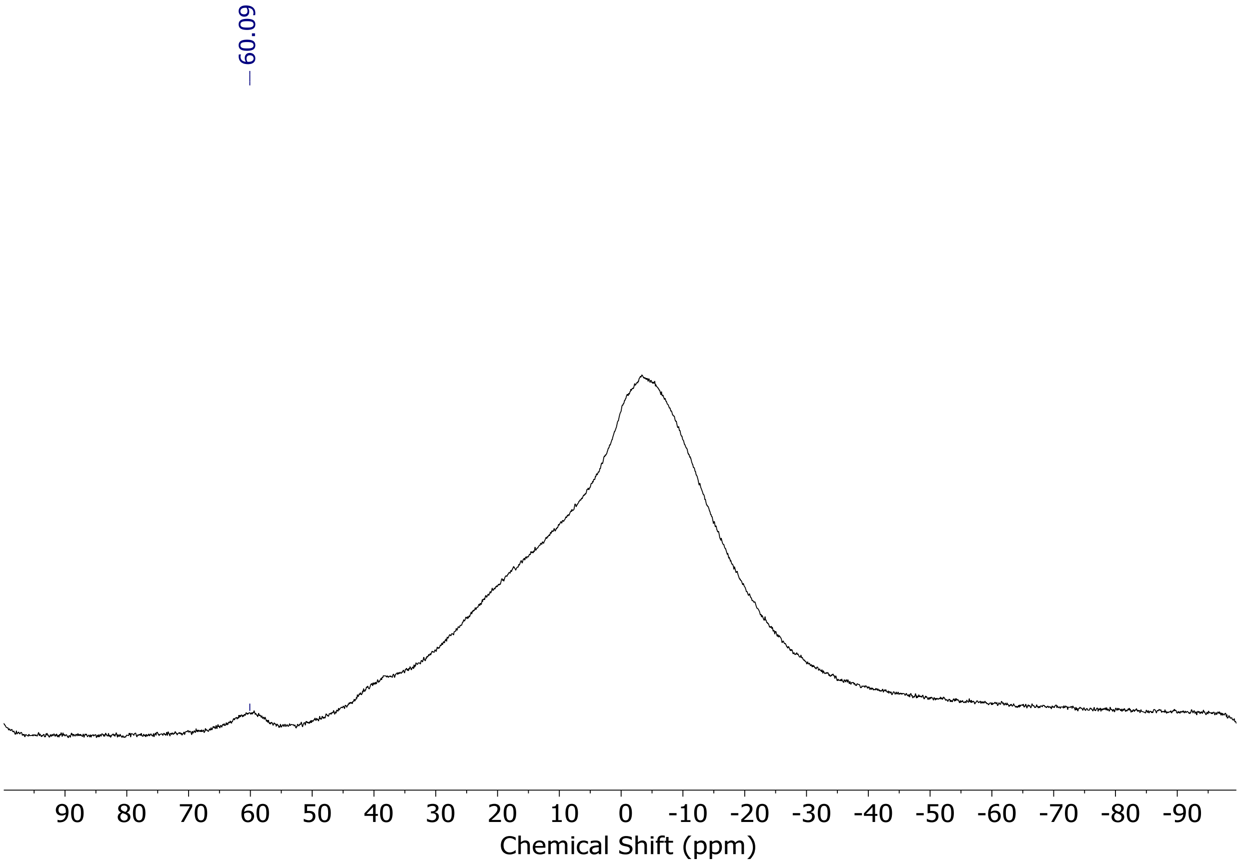

Figure S38. *In situ* $^{11}B\{^1H\}$ NMR spectrum ($CDCl_3$, 160 MHz) of a 1:1 mixture of $Dipp_3PO$ and $B(C_6F_5)_3$.

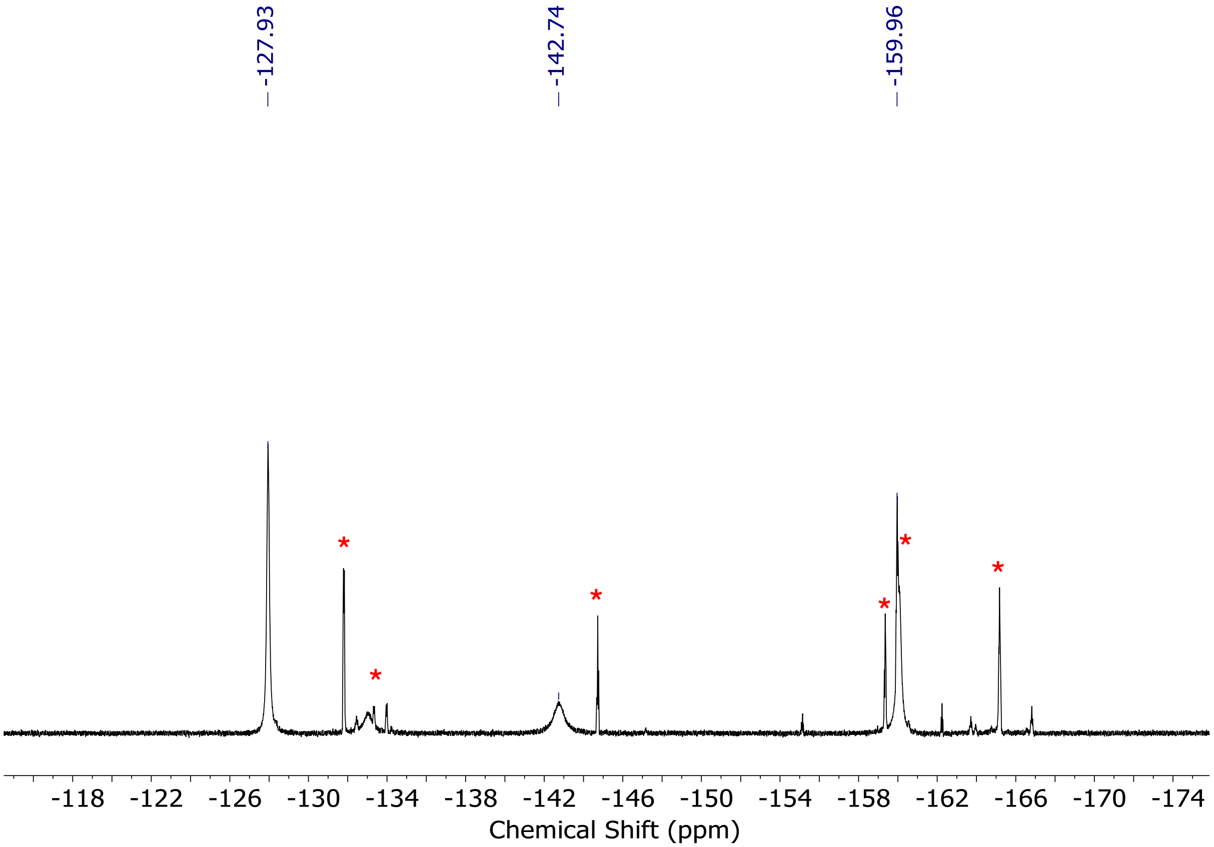

Figure S39. *In situ* $^{19}F\{^1H\}$ NMR spectrum ($CDCl_3$, 470 MHz) of a 1:1 mixture of $Dipp_3PO$ and $B(C_6F_5)_3$. The asterisks denote signals that correspond to $Et_2O{\cdot}B(C_6F_5)_3$ and salts of the $[B(C_6F_5)_3(OH)]^-$ anion.

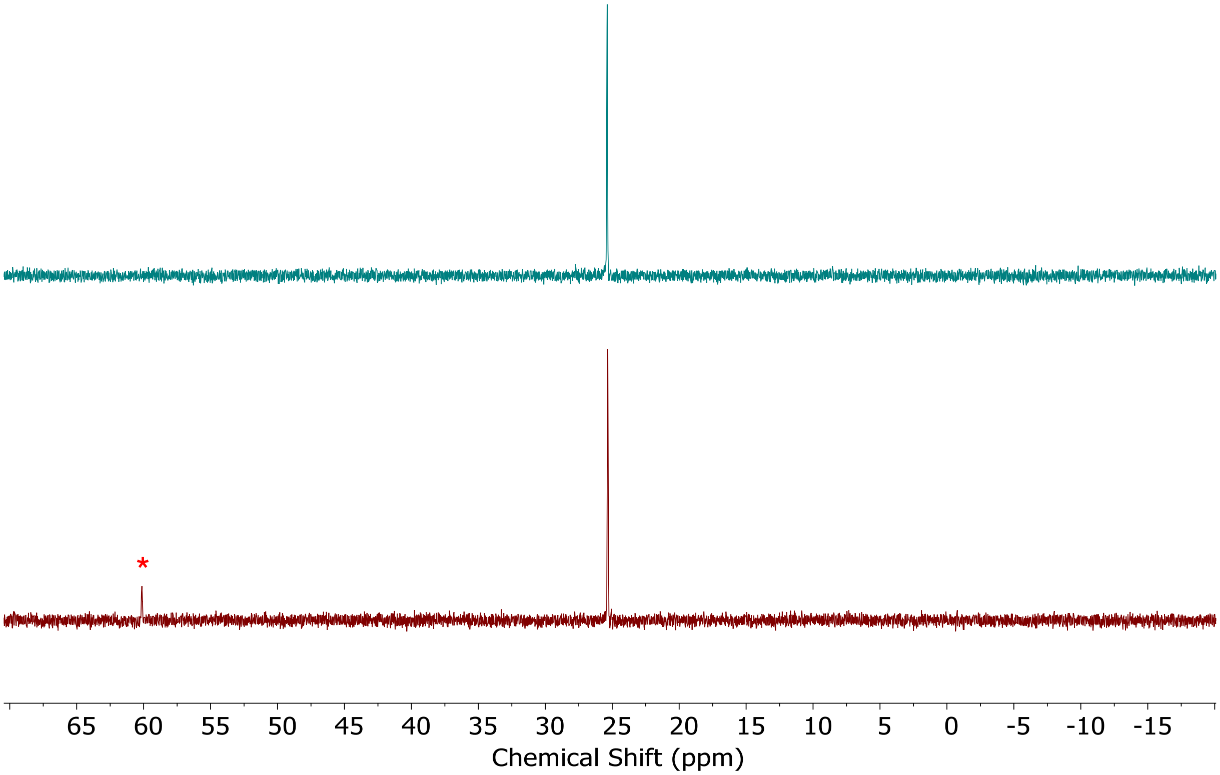

Figure S40. *In situ* $^{31}P\{^1H\}$ NMR spectrum ($CDCl_3$, 202 MHz) of $Dipp_3PO$ (*top*) and a 1:1 mixture of $Dipp_3PO$ and $B(C_6F_5)_3$ (*bottom*) The asterisk denotes a signal that correspond to residual $Dipp_3P$ in the sample.

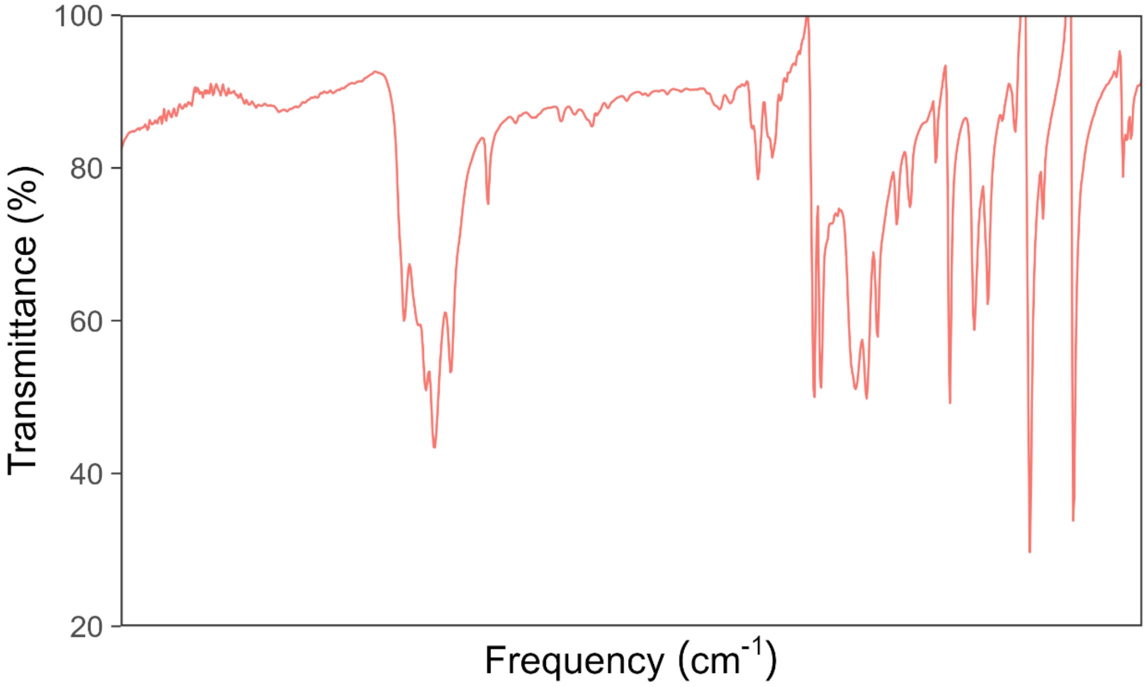

Figure S41. Experimental IR spectrum (KBr pellet) of $(3,5\text{-}Me_2Ph)_3Sb$.

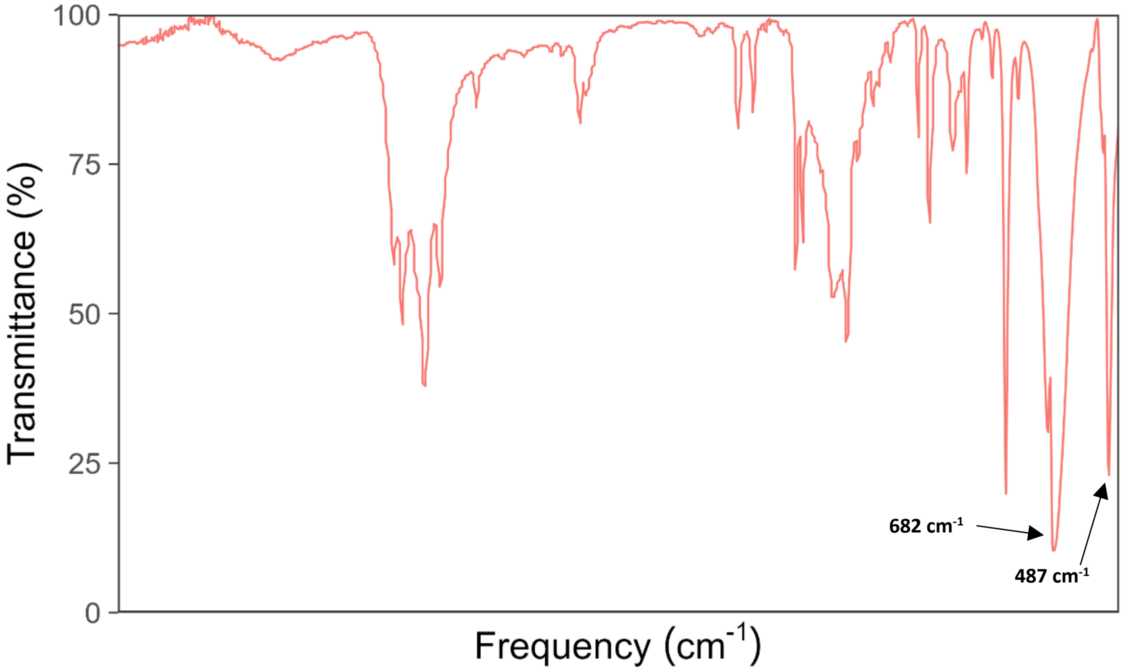

Figure S42. Experimental IR spectrum (KBr pellet) of [(3,5-Me_2Ph)$_3$SbO]$_n$ (ν_{SbO} = 682 cm^{-1} and δ_{SbO} = 487 cm^{1}).

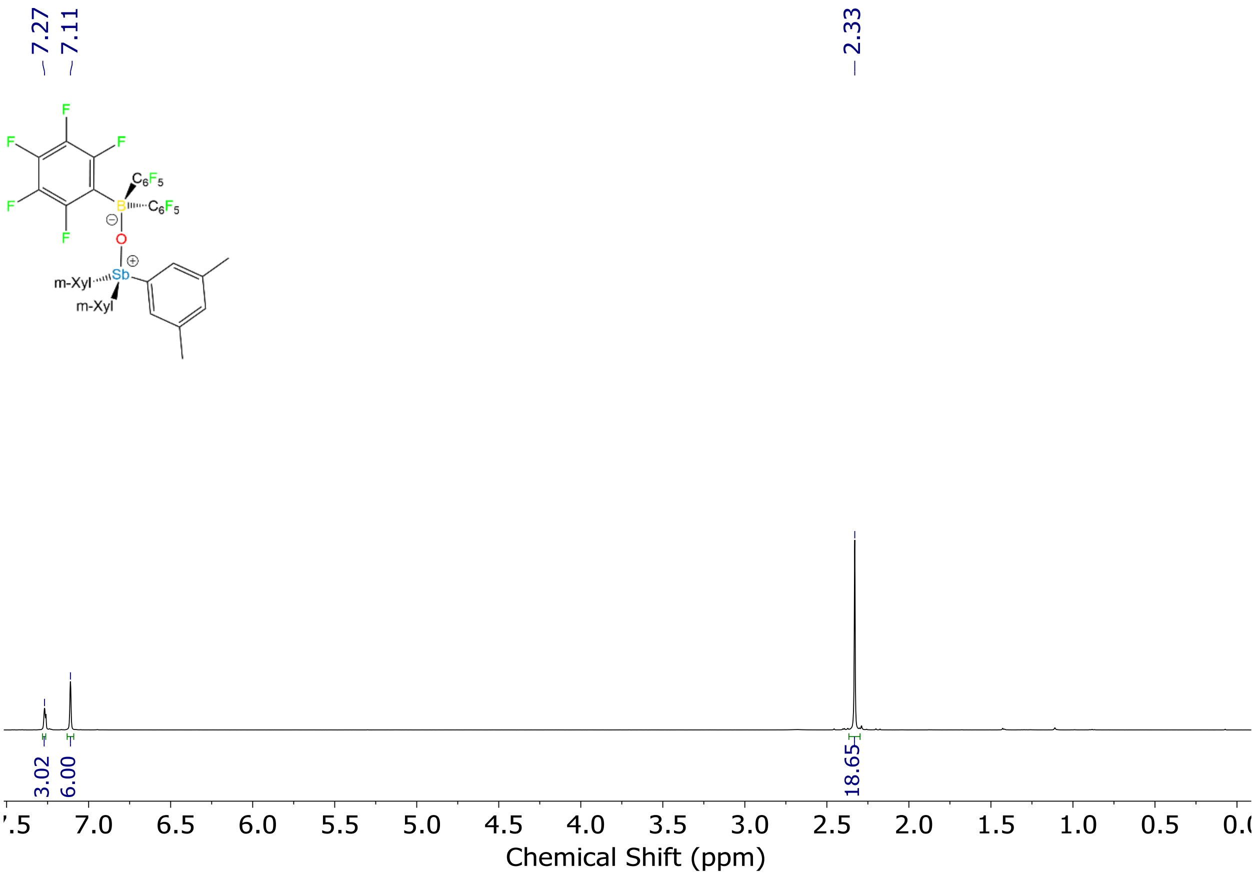

Figure S43. ^{1}H NMR spectrum ($CDCl_3$, 500 MHz) of (3,5-Me_2Ph)$_3$SbO·B(C_6F_5)$_3$.

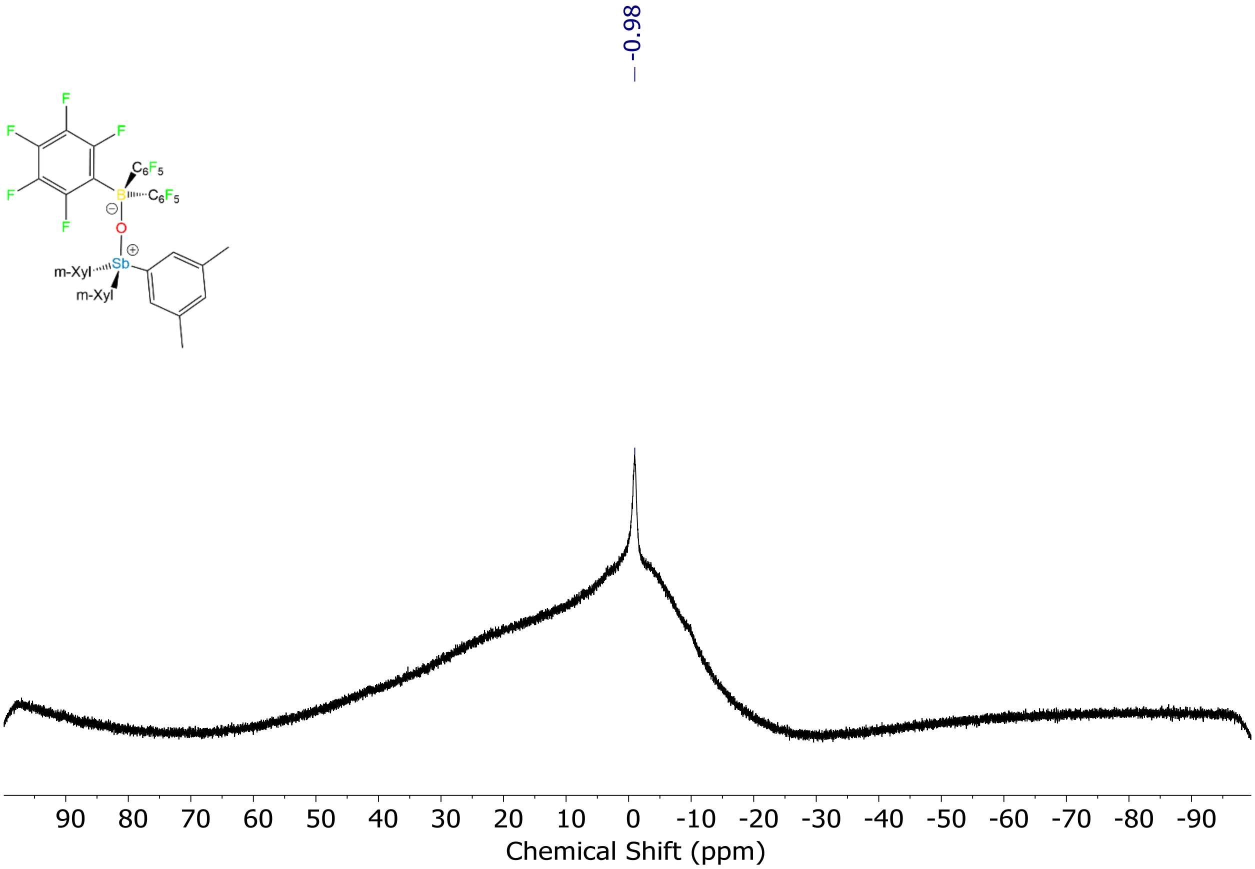

Figure S44. $^{11}B\{^{1}H\}$ NMR spectrum ($CDCl_3$, 160 MHz) of $(3,5\text{-}Me_2Ph)_3SbO\cdot B(C_6F_5)_3$.

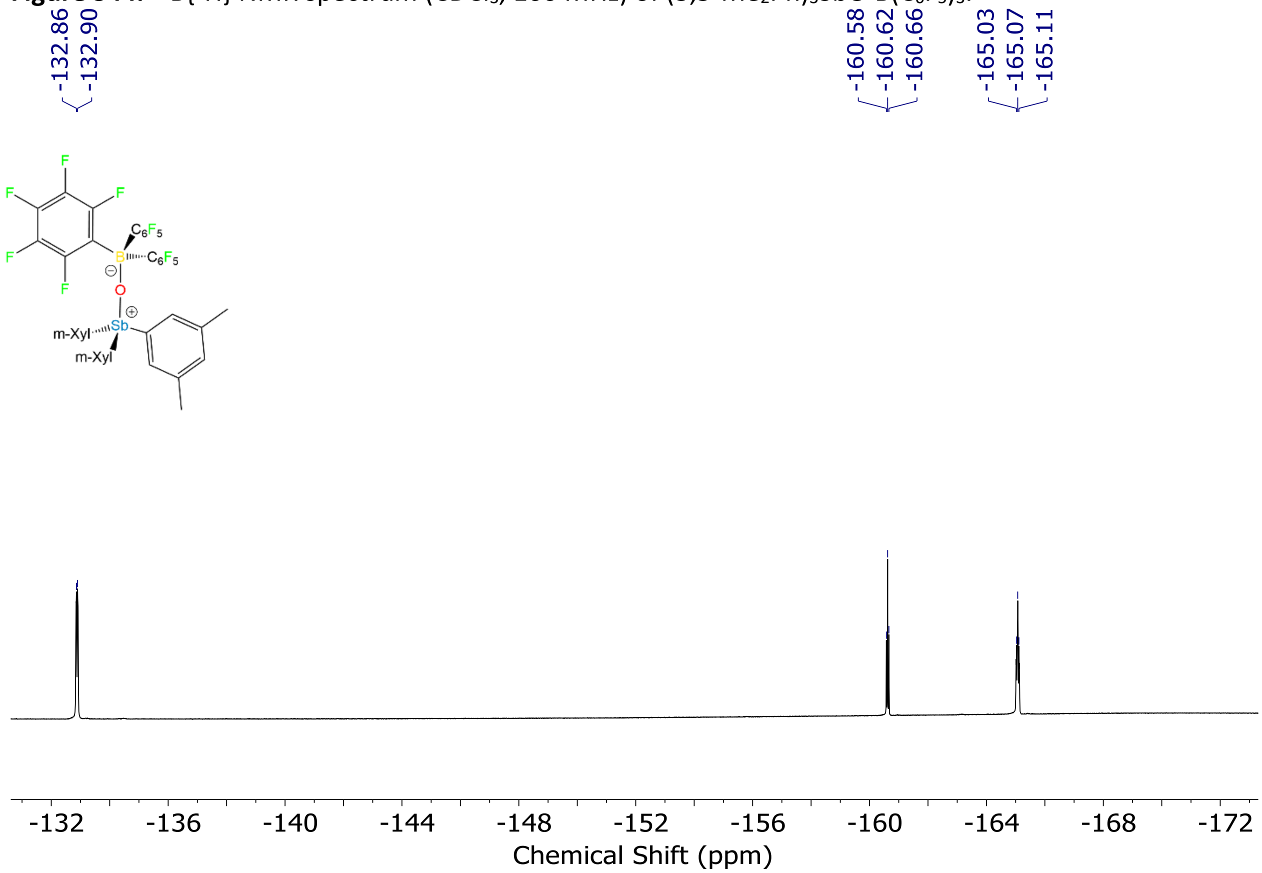

Figure S45. $^{19}F\{^{1}H\}$ NMR spectrum ($CDCl_3$, 470 MHz) of $(3,5\text{-}Me_2Ph)_3SbO\cdot B(C_6F_5)_3$.

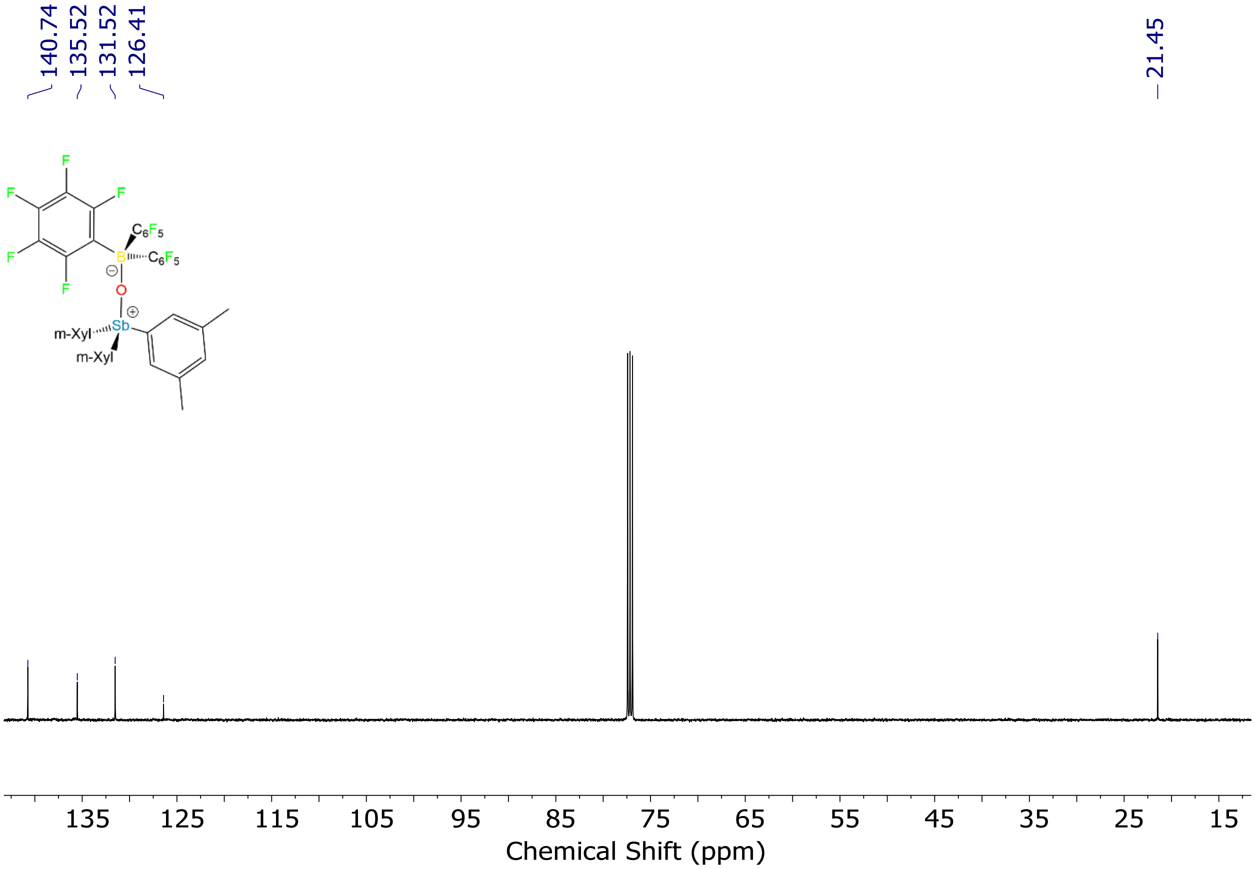

Figure S46. $^{13}C\{^1H\}$ NMR spectrum ($CDCl_3$, 125 MHz) of (3,5-$Me_2Ph)_3SbO\cdot B(C_6F_5)_3$.

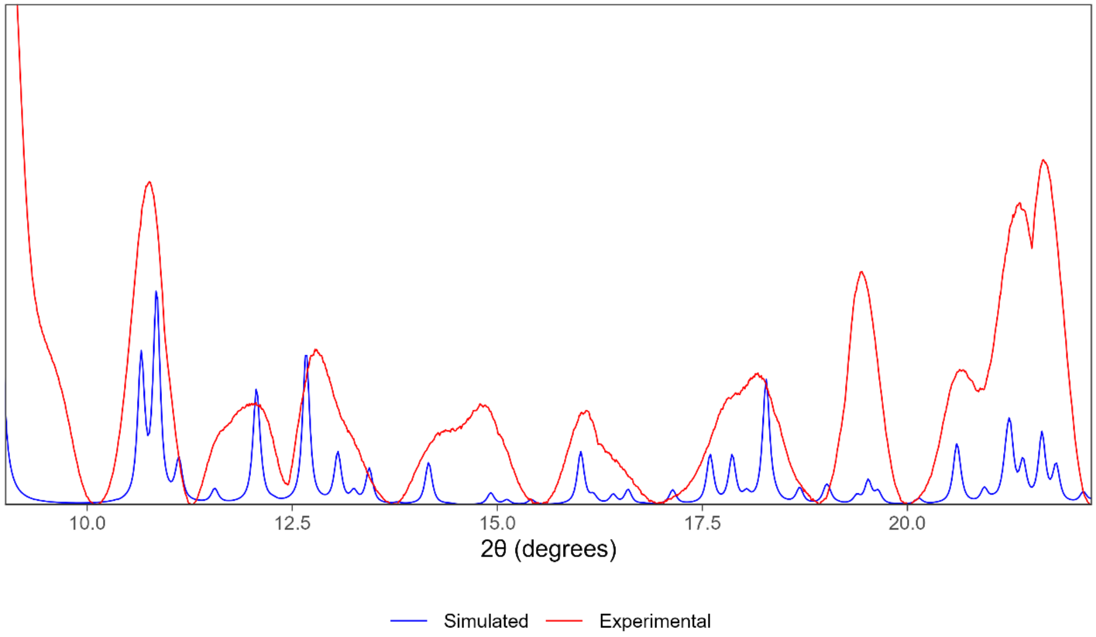

Figure S47. Simulated and experimental PXRD diffractogram of (3,5-$Me_2Ph)_3SbO\cdot B(C_6F_5)_3$.

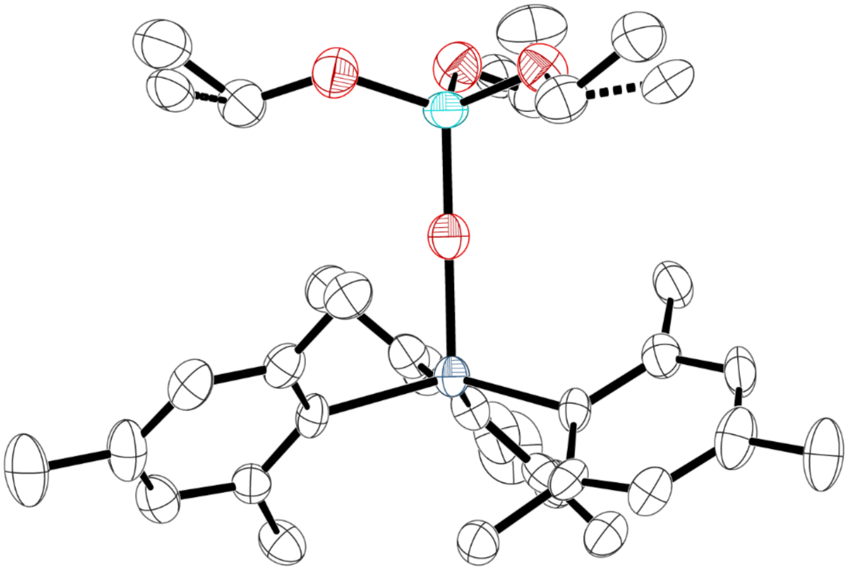

Figure S48. Thermal ellipsoid plot (50% probability) of $Mes_3SbO{\cdot}Al(OEt)_3$. Color code: Sb navy, Al teal, O red, and C black. Hydrogen atoms are omitted for clarity.

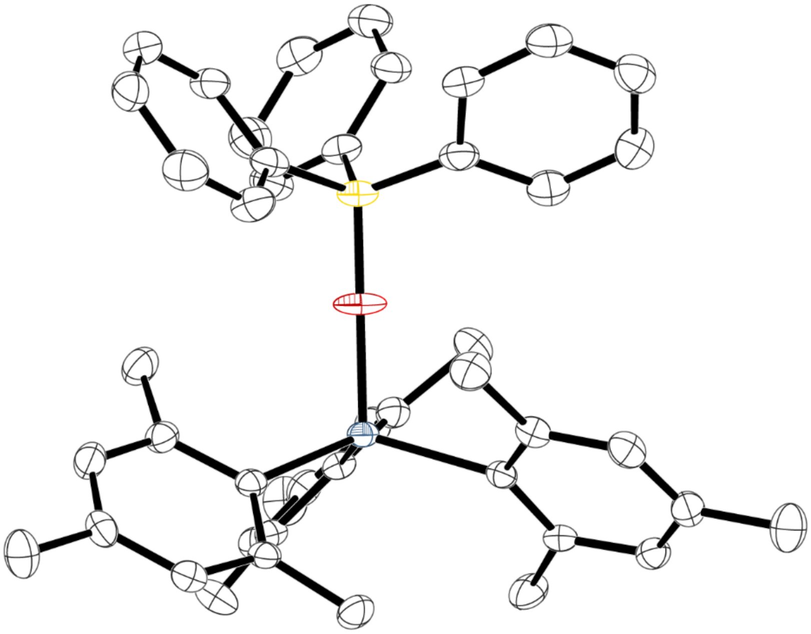

Figure S49. Thermal ellipsoid plot (50% probability) of $Mes_3SbO{\cdot}BPh_3$. Color code: Sb navy, B yellow, O red, and C black. Hydrogen atoms are omitted for clarity.

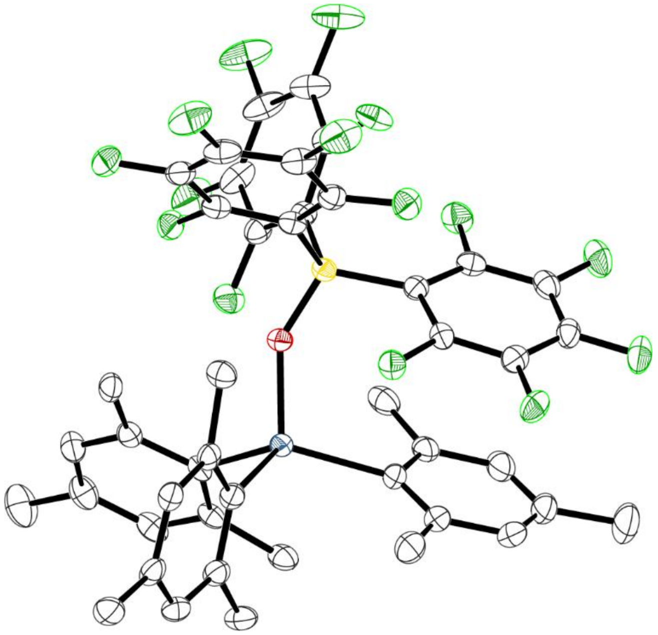

Figure S50. Thermal ellipsoid plot (50% probability) of $Mes_3SbO{\cdot}B(C_6F_5)_3{\cdot}(CHCl_3)$. Color code: Sb navy, B yellow, O red, F green, and C black. Hydrogen atoms and chloroform solvate disorder are omitted for clarity.

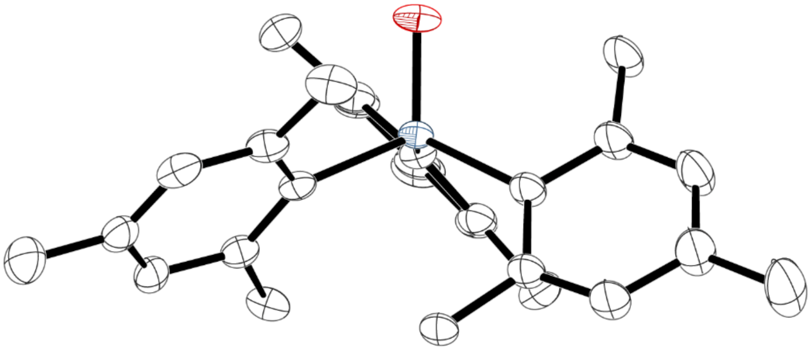

Figure S51. Thermal ellipsoid plot (50% probability) of Mes_3AsO. Color code: As navy, O red, and C black. Hydrogen atoms are omitted for clarity.

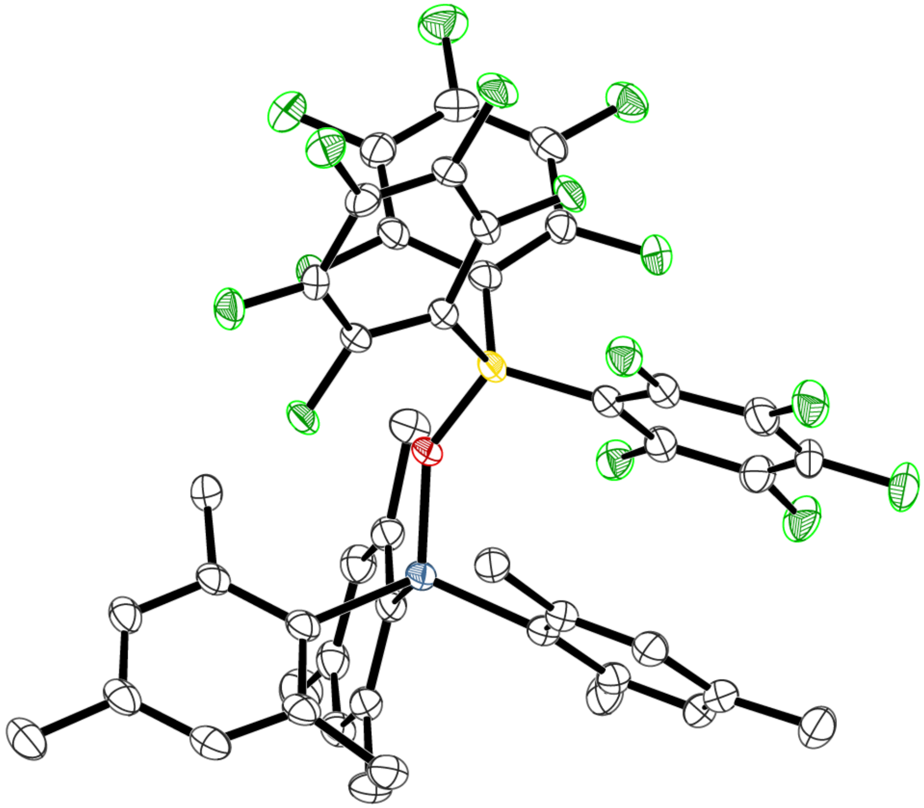

Figure S52. Thermal ellipsoid plot (50% probability) of $Mes_3AsO{\cdot}B(C_6F_5)_3$. Color code: As navy, B yellow, O red, and C black. Hydrogen atoms and dichloromethane solvate disorder are omitted for clarity.

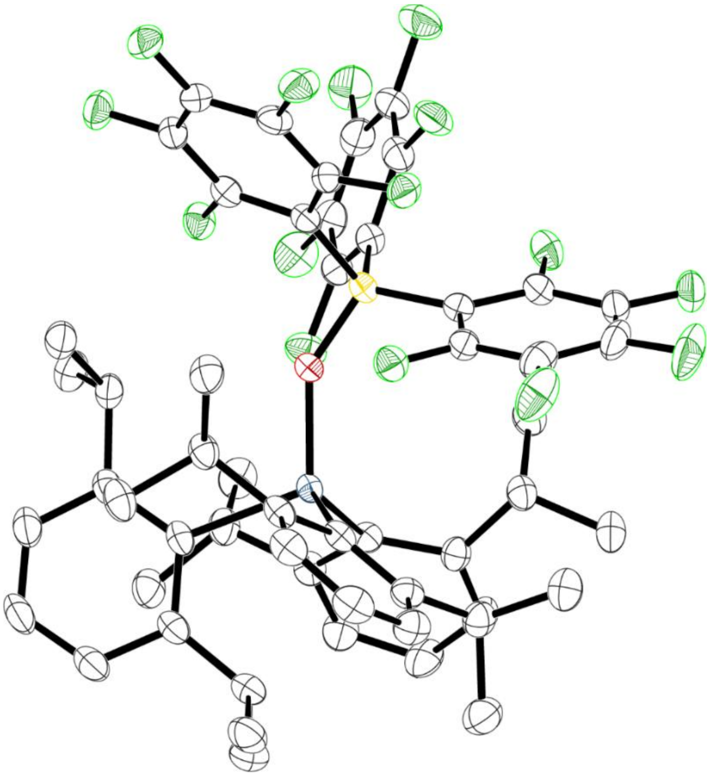

Figure S53. Thermal ellipsoid plot (50% probability) of $Dipp_3SbO{\cdot}B(C_6F_5)_3$. Color code: Sb navy, B yellow, F green, O red, and C black. Hydrogen atoms are omitted for clarity.

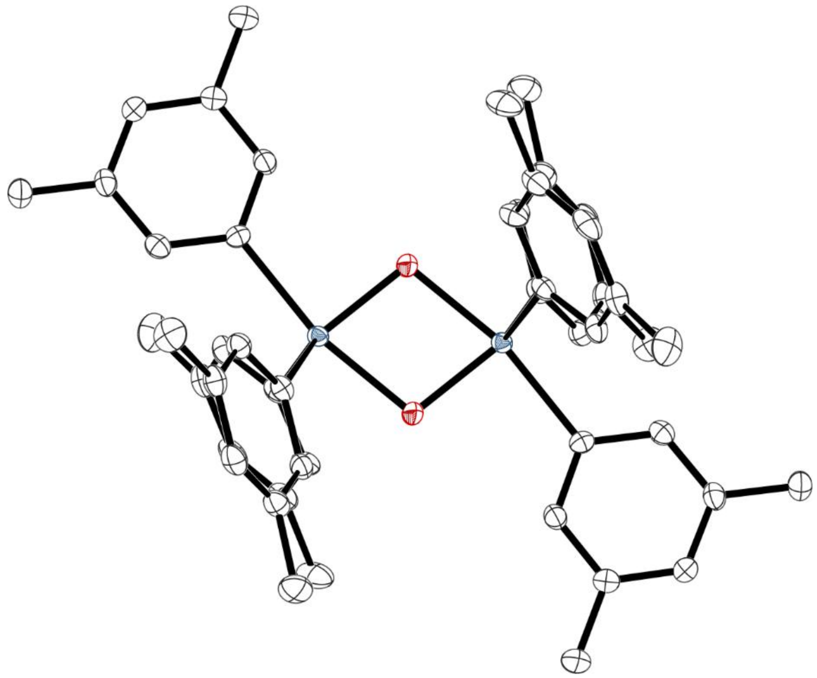

Figure S54. Thermal ellipsoid plot (50% probability) of $((3,5\text{-}Me_2Ph)_3SbO)_2$. Color code: Sb navy, O red, and C black. Hydrogen atoms are omitted for clarity.

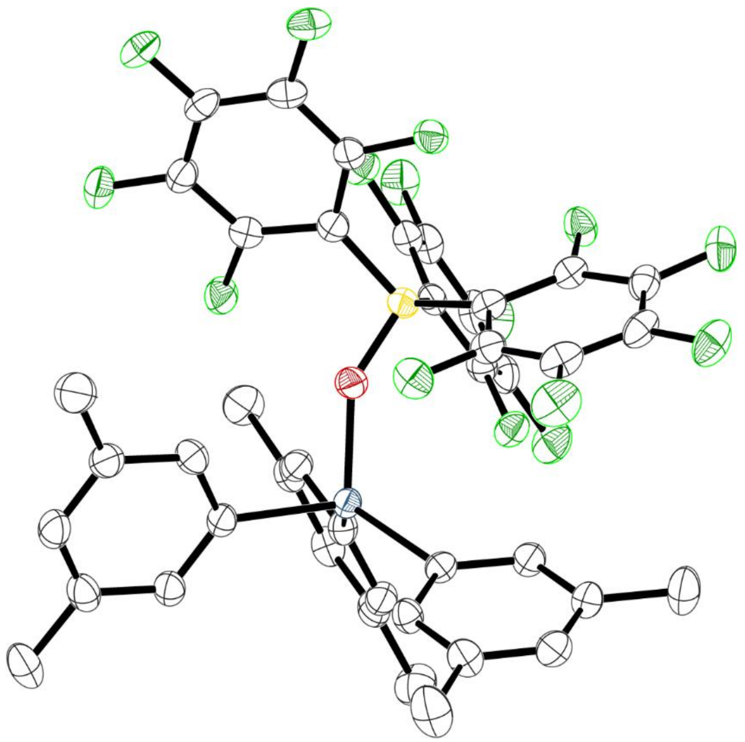

Figure S55. Thermal ellipsoid plot (50% probability) of $(3,5\text{-}Me_2Ph)_3SbO{\cdot}B(C_6F_5)_3$. Color code: Sb navy, B yellow, F green, O red, and C black. Hydrogen atoms are omitted for clarity.

Table S1. Crystallographic details for $Mes_3SbO{\cdot}Al(OEt)_3$, $Mes_3SbO{\cdot}BPh_3$, $Mes_3SbO{\cdot}B(C_6F_5)_3{\cdot}(CHCl_3)$, and $Dipp_3SbO{\cdot}B(C_6F_5)_3$.

Compound	$Mes_3SbO{\cdot}Al(OEt)_3$	$Mes_3SbO{\cdot}BPh_3$	$Mes_3SbO{\cdot}B(C_6F_5)_3{\cdot}(CHCl_3)$	$Dipp_3SbO{\cdot}B(C_6F_5)_3$
Empirical formula	$C_{33}H_{48}O_4AlSb$	$C_{45}H_{48}BOSb$	$C_{46}H_{34}BCl_3F_{15}OSb$	$C_{54}H_{51}BOF_{15}Sb$
Formula Weight	657.44	737.39	1126.64	1133.51
Temperature (K)	100.5(10)	105(7)	108(2)	100.0(3)
Wavelength (Å)	1.54184	1.54184	1.54184	1.54184
Crystal system	Trigonal	Trigonal	Orthorhombic	Monoclinic
Space group	$P\bar{3}c1$	$P\bar{3}$	$P2_12_12_1$	$P2_1/n$
a (Å)	14.46380(10)	14.2645(2)	15.81490(10)	12.54580(10)
b (Å)			16.33360(10)	17.7300(2)
c (Å)	18.0532(2)	10.4374(2)	17.34800(10)	22.0529(2)
β (°)				94.4050(10)
Volume (Å^3)	3270.77(6)	1839.23(6)	4481.24(5)	4890.89(8)
Z	4	2	4	4
ρ_{calc} (Mg/m^3)	1.335	1.332	1.670	1.539
Crystal size (mm^3)	0.11 × 0.07 ×	0.09 × 0.02 × 0.02	0.27 × 0.21 × 0.10	0.12 × 0.06 × 0.04
θ range (°)	3.529 to 70.072	3.578 to 68.245	3.717 to 68.249	3.202 to 68.249
Total reflections	40038	23453	63600	50621
Unique reflections	2088	2260	8213	8814
Parameters	133	148	651	661
Completeness	100.0	100.0	100.0	98.7
R_{int}	0.0378	0.0610	0.0489	0.0429
R_1 ($I > 2\sigma$)	0.0771	0.0241	0.0253	0.0250
R_1 (all data)	0.0803	0.0271	0.0258	0.0292
wR_2 ($I > 2\sigma$)	0.2079	0.0560	0.0692	0.0548
wR_2 (all data)	0.2099	0.0568	0.0697	0.0562
Goodness of fit, *S*	1.121	1.043	1.049	1.033

Table S2. Crystallographic details for $((3,5\text{-}Me_2Ph)_3SbO)_2$, $(3,5\text{-}Me_2Ph)_3SbO{\cdot}B(C_6F_5)_3$, Mes_3AsO, and $Mes_3AsO{\cdot}B(C_6F_5)_3{\cdot}(CH_2Cl_2)$.

Compound	$((3,5\text{-}Me_2Ph)_3SbO)_2$	$(3,5\text{-}Me_2Ph)_3SbO{\cdot}B(C_6F_5)_3$	Mes_3AsO	$Mes_3AsO{\cdot}B(C_6F_5)_3{\cdot}(CH_2Cl_2)$
Empirical formula	$C_{24}H_{27}OSb$	$C_{42}H_{27}BOF_{15}Sb$	$C_{27}H_{33}AsO$	$C_{46}H_{35}AsBCl_2F_{15}O$
Formula Weight	453.20	965.19	448.45	1045.37
Temperature (K)	102(3)	100.0(8)	104.15	105(8)
Wavelength (Å)	1.54184	1.54184	1.54184	1.54184
Crystal system	Monoclinic	Monoclinic	Monoclinic	Monoclinic
Space group	$P2_1/n$	$P2_1/c$	$P2_1/c$	$P2_1/c$
a (Å)	9.69870(10)	17.4502(2)	17.8409(3)	19.5837(2)
b (Å)	16.3757(2)	14.58350(10)	8.45770(10)	21.8721(2)
c (Å)	13.5494(2)	16.1000(2)	16.2241(2)	20.0306(2)
β (°)	98.8560(10)	108.1130(10)	106.368(2)	98.2490(10)
Volume ($Å^3$)	2126.30(5)	3894.17(7)	2348.88(6)	8491.07(15)
Z	4	4	4	8
ρ_{calc} (Mg/m^3)	1.416	1.646	1.268	1.635
Crystal size (mm^3)	0.07 × 0.04 × 0.04	0.12 × 0.10 × 0.05	0.1 × 0.07 × 0.04	0.14 × 0.08 × 0.06
θ range (°)	4.265 to 68.251	2.664 to 68.244	2.581 to 68.232	2.28 to 68.249
Total reflections	14500	26809	27661	150852
Unique reflections	3883	7106	4298	15522
Parameters	241	547	271	1235
Completeness	99.7	99.7	100	99.9
R_{int}	0.0286	0.0414	0.0365	0.0501
R_1 ($I > 2\sigma$)	0.0230	0.0311	0.0469	0.0385
R_1 (all data)	0.0276	0.0370	0.0519	0.0428
wR_2 ($I > 2\sigma$)	0.0522	0.0785	0.1281	0.0964
wR_2 (all data)	0.0536	0.0815	0.1326	0.0988
Goodness of fit, *S*	1.042	1.035	1.075	1.032
